# Identification of Benzothiazoles Bearing 1,3,4-Thiadiazole as Antiproliferative Hybrids Targeting VEGFR-2 and BRAF Kinase: Design, Synthesis, BIO Evaluation and In Silico Study

**DOI:** 10.3390/molecules29133186

**Published:** 2024-07-04

**Authors:** Wafaa A. Ewes, Samar S. Tawfik, Aya M. Almatary, Mashooq Ahmad Bhat, Hamed W. El-Shafey, Ahmed A. B. Mohamed, Abdullah Haikal, Mohammed A. El-Magd, Abdullah A. Elgazar, Marwa Balaha, Abdelrahman Hamdi

**Affiliations:** 1 Department of Pharmaceutical Organic Chemistry, Faculty of Pharmacy, Mansoura University, Mansoura 35516, Egypt; wafaa_ewes@mans.edu.eg (W.A.E.); drsamarelmasry@mans.edu.eg (S.S.T.); hamedelshafey@mans.edu.eg (H.W.E.-S.); 2 Department of Pharmaceutical Organic Chemistry, Faculty of Pharmacy, Horus University-Egypt, New Damietta 34518, Egypt; aelmatary@horus.edu.eg; 3 Department of Pharmaceutical Chemistry, College of Pharmacy, King Saud University, P.O. Box 2457, Riyadh 11451, Saudi Arabia; mabhat@ksu.edu.sa; 4 Department of Medicinal Chemistry, Faculty of Pharmacy, Mansoura University, Mansoura 35516, Egypt; ahmed_bakr@mans.edu.eg; 5 Department of Pharmacognosy, Faculty of Pharmacy, Mansoura University, Mansoura 35516, Egypt; abdullahhaikal@mans.edu.eg; 6 Department of Anatomy, Faculty of Veterinary Medicine, Kafrelsheikh University, Kafrelsheikh 33516, Egypt; mohamed.abouelmagd@vet.kfs.edu.eg; 7 Department of Pharmacognosy, Faculty of Pharmacy, Kafrelsheikh University, Kafrelsheikh 33516, Egypt; abdulah.elgazar@phr.mans.edu.eg; 8 Department of Medical, Oral and Biotechnological Sciences, “G. d’Annunzio” University of Chieti-Pescara, Via dei Vestini, 31, 66100 Chieti, Italy; marwa.balaha@unich.it

**Keywords:** benzothiazoles, antitumor activity, VEGFR-2 inhibition, BRAF inhibition, cell cycle analysis, apoptosis, molecular docking

## Abstract

Cancer remains a leading cause of death worldwide, often resulting from uncontrolled growth in various organs. Protein kinase inhibitors represent an important class of targeted cancer therapies. Recently, the kinases BRAF and VEGFR-2 have shown synergistic effects on tumor progression. Seeking to develop dual BRAF/VEGFR-2 inhibitors, we synthesized 18 amino-benzothiazole derivatives with structural similarities to reported dual inhibitors. Four compounds—**4a**, **4f**, **4l**, and **4r**—demonstrated remarkable cytotoxicity, with IC_50_ values ranging from 3.58 to 15.36 μM, against three cancer cell lines. Furthermore, these compounds showed IC_50_ values of 38.77–66.22 μM in the case of a normal cell line, which was significantly safer than the reference, sorafenib. Subsequent investigation revealed that compound **4f** exhibited the capacity to inhibit the BRAF and VEGFR-2 enzymes, with IC_50_ values similar to sorafenib (0.071 and 0.194 μM, respectively). Moreover, compound **4f** caused G2-M- and S-phase cycle arrest. Molecular modeling demonstrated binding patterns compatible with inhibition for both targets, where **4f** exerted the critical interactions in the BRAF site and interacted in the VEGFR-2 site in a manner akin to sorafenib, demonstrating affinity similar to dabrafenib.

## 1. Introduction

Targeted therapeutics is one of the most promising approaches for achieving such a goal. Protein kinase inhibitors represent an important and emerging class of targeted therapeutic agents [1]. Among the different types of kinases, tyrosine kinases are vital in regulating various physiological and biochemical responses in the human body, such as cell growth, differentiation, and death, and in regulating normal cellular processes [2,3]. In addition, uncontrolled receptor tyrosine kinase (RTK) signaling controls the development and progression of numerous cancers [2,4].

Vascular endothelial growth factors (VEGFs) are considered one of the most significant categories of tyrosine kinases. They engage with the kinase domains of three VEGF receptors (VEGFRs 1–3) in a manner that overlaps [5]. VEGFR-2 typically resides on the endothelial layer of blood vessels. It is often regarded as the most crucial regulator in the process of angiogenesis [6]. Stimulation of VEGFR-2 enhances tumor proliferation and metastasis via activation of a downstream signaling pathway, leading to tumor angiogenesis [7,8]. Accordingly, preventing or down-regulating VEGFR-2 signaling is a very successful strategy for inhibiting the angiogenesis of a tumor and, as a result, impeding tumor proliferation [9,10,11]. 

Conversely, the rapidly accelerated fibrosarcoma (RAF) kinases are protein kinases that specifically phosphorylate serine and threonine residues. They facilitate the RAS-RAF-MEK-ERK pathway, resulting in the stimulation of several transcription factors [12]. This RAS signal transduction promotes cellular growth, differentiation, and proliferation of many human tumors [13]. BRAF is the most prevalent RAF kinase among the three known isoforms, primarily because it is more easily activated than the other RAF isoforms. The substitution of valine (V) at position 600 with a glutamic acid (E) is the most prevalent oncogenic mutation in BRAF, known as V600E [14,15]. It was reported that BRAF^V600E^ has a vital role in increasing constitutive kinase activity, inducing the production of VEGF and subsequently activating its receptor (VEGFR-2) [16,17]. The inhibition of BRAF has excellent potential in developing and identifying useful substances for cancer treatment [18].

Recent studies have demonstrated that the combination of BRAF and VEGFR-2 has a mutually reinforcing impact on the proliferation and progression of cancer [19]. Therefore, multi-kinase inhibition of BRAF and VEGFR-2 is a potential cancer therapy approach [14,18,20]. 

**Figure 1:**
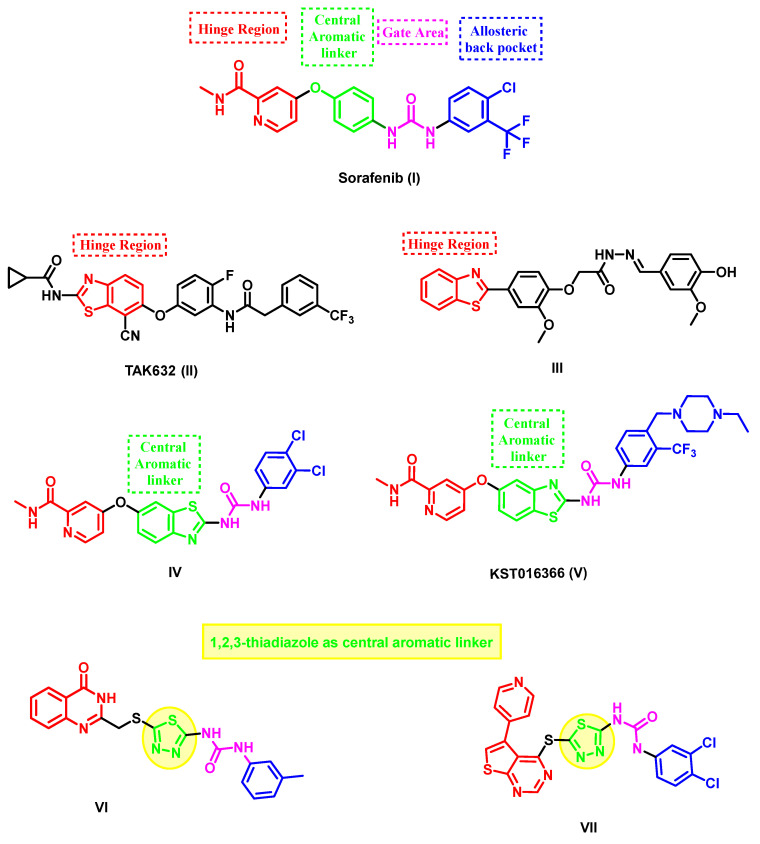
Structural features of some VEGFR-2 and BRAF inhibitors: sorafenib, benzothiazole, and thiadiazole-based inhibitors.

Sorafenib (Nexavar^®^, **I**) (Figure 1) has been reported to be an effective VEGFR-2 inhibitor. Additionally, it is the first approved RAF inhibitor [21]. Unfortunately, many drawbacks are associated with its use as an accepted anticancer agent, for example, its poor multi-kinase selectivity [22]. Therefore, sorafenib’s structural optimization has received significant attention from medicinal chemists [23,24,25,26].

Benzothiazole is a privileged molecular skeleton that is widely used in the development of a variety of anticancer agents [27,28]. Various benzothiazole derivatives have been developed with unique and promising multi-kinase inhibition profiles, especially against VEGFR-2 and BRAF [29,30]. In several studies, the benzothiazole core served as a sorafenib congener by replacing either the picolinamide hinge binder or the central phenyl linker of sorafenib [27]. As an example, TAK-632 (**II**), a compound derived from 2-amino-benzothiazole, effectively inhibits the activity of BRAF^V600E^ (with an IC_50_ value of 2.4 nM) and has a high level of selectivity toward BRAF [31]. Compound **III**, which contains a 2-phenylbenzothiazole moiety, was found to have a strong inhibitory effect on several kinases. It specifically targets the receptor tyrosine kinase (RTK)-binding site in the hinge region. The compound had an IC_50_ value of 0.17 µM, 0.19 µM, and 0.08 µM against VEGFR-2, FGFR-1, and PDGFR-β, respectively. The study demonstrated an 83% inhibitory effect on VEGFR-2 in MCF-7 cells compared to sorafenib (**III**), which showed 88% inhibition [32]. A novel line of 2-amino benzothiazole congeners with dual inhibitory activity against BRAFV600E and CRAF was created by replacing the central phenyl ring with a benzothiazole core in the structure of sorafenib. Compound **IV** had the highest inhibitory activity, with IC_50_ values of 95 nM and 15 nM against BRAFV600 and CRAF, respectively [33]. Similarly, compound KST016366 (**V**) was found to inhibit two essential angiogenic kinases: VEGFR2 and Tie2 (Figure 1) [34]. 

## 2. Results and Discussion

### 2.1. Design Rational

**Figure 2:**
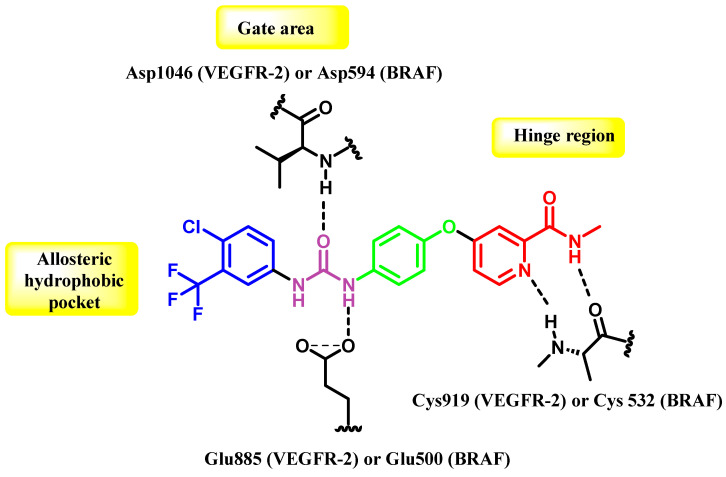
The interactions between sorafenib (**I**) and the specific active regions of VEGFR-2 and BRAF.

The co-crystal structure of VEGFR-2 and BRAF demonstrates that they possess overlapping areas in their active sites, namely the front cleft, gate area, and hydrophobic back cleft located beyond the gatekeeper residue [35,36]. Therefore, dual VEGFR-2/BRAF inhibitors like sorafenib possess important interaction characteristics that can fulfill the binding needs of both kinases’ active sites, even though there may be variations in other parts of their active sites. The compound possesses a planar heteroaromatic core, which establishes hydrogen bonds with the hinge area residues Cys919 (VEGFR-2) and Cys532 (BRAF). A hydrophobic linker ring is present in the following allosteric site. In addition, a urea or amide functional group serves as both a hydrogen bond donor and an acceptor, engaging with specific residues such as Glu885 (VEGFR-2) and Glu500 (BRAF), as well as the aspartates Asp1046 and Asp594 in the DFG motif for VEGFR-2 and BRAF, respectively. Ultimately, a fragrant component protrudes into both enzymes’ shared hydrophobic rear pocket (Figure 2) [37,38,39]. 

**Figure 3:**
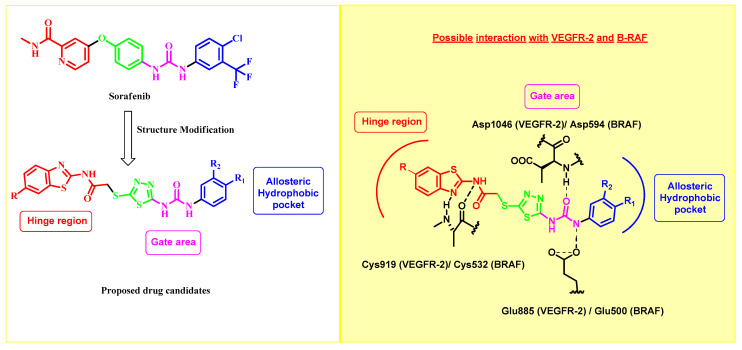
Design strategy for 2-amino-benzothiazole derivatives as dual VEGFR-2/BRAF inhibitors.

Based on the information above, our study aimed to include the benzothiazole-2-amide molecule in the hinge region (front pocket). The presence of the methylene group in the acetamide structure is crucial for enhancing the binding affinity toward both VEGFR-2 and B-RAF via increasing the flexibility. Furthermore, the 1,3,4-thiadiazole motif was incorporated as a bioisostere for the central phenyl ring of sorafenib. This motif links the hinge binder with the urea motif, which occupies the central gate region of the inactive DFG-out conformation of both enzymes. It plays a crucial role in forming the necessary hydrogen bonds. As seen in the sorafenib congener **VI**, the 1,3,4-thiadiazole motif has been documented as an effective isostere for the central phenyl ring [40] and **VII** [41] (Figure 1)**,** demonstrating strong inhibitory efficacy against VEGFR-2. Ultimately, the hydrophobic substituent located on the urea moiety will fill the allosteric hydrophobic back pocket (Figure 3).

### 2.2. Chemistry

**Scheme 1:**
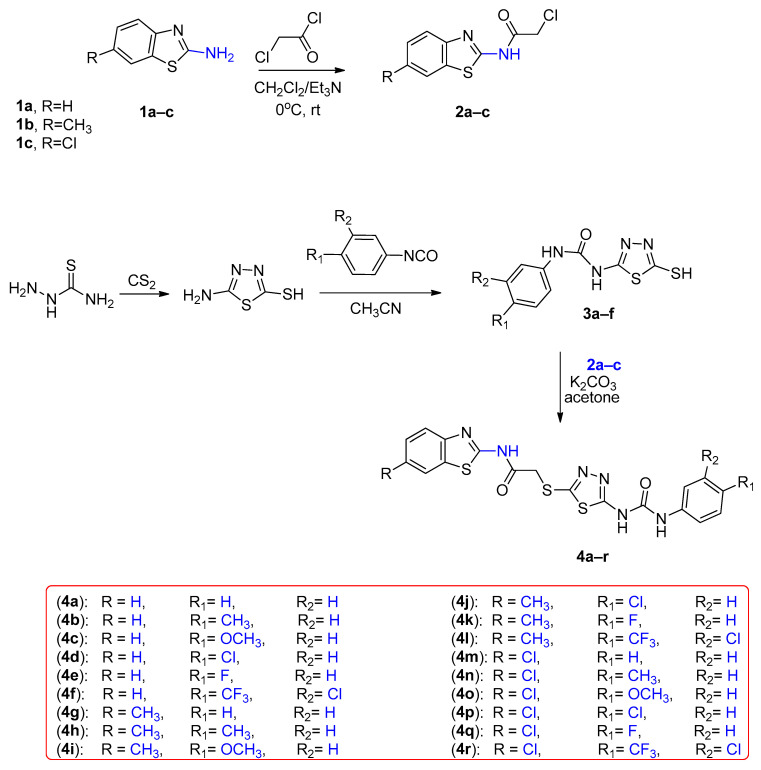
Synthesis of benzothiazole hybrids **4a**–**r**.

The process of creating the novel hybrids, specifically thiadiazole thioacetamide **4a**–**r**, is illustrated in Scheme 1. By initiating the process using benzo[d]thiazol-2-amine derivatives **1a**–**c**, we subjected them to acylation using chloroacetyl chloride in the presence of a base, specifically triethylamine. As a result, we successfully obtained the chloroacetamide derivatives **2a**–**c** [42,43]. 

On the other hand, the 5-amino-1,3,4-thiadiazole-2-thiol was furnished via cyclisation of thiosemicarbazide with carbon disulfide. Upon reaction of the thiadiazole derivative with various phenyl isocyanates, **3a**–**f** were acquired [42]. Our target compounds were obtained in a good yield on the reaction of the chloroacetamide derivatives **2a**–**c** with the thiadiazole-2-thiol derivatives **3a**–**f** in acetone and potassium carbonate as a base.

The structures of the final compounds were proved by NMR (see Supplementary Materials). The ^1^H-NMR spectra of the new compounds exhibited a distinct singlet peak between 4.30 and 4.35 ppm, corresponding to the methylene protons (-SCH_2_CO-). This peak confirms the creation of our novel hybrids. Furthermore, the ^13^C-NMR spectral analysis revealed the presence of two signals within the 160–175 ppm range, thereby verifying the presence of two carbonyl groups. 

### 2.3. Biological Evaluation

#### 2.3.1. In Vitro Antitumor Activity and SAR Correlation 

The in vitro activity of compounds **4a**–**r** was evaluated against three human cancer cell lines, namely hepatocellular carcinoma (HePG-2), breast cancer (MCF-7), and colorectal carcinoma (HCT-116), using the MTT standard assay. Sorafenib was used as a positive control medication. The IC_50_ values obtained from the in vitro anticancer activities are displayed in Table 1.

The findings indicated that compounds **4a**, **4f**, **4l**, and **4r** exhibited the most inhibitory effects and had wide-ranging anticancer activity against the three tumor cell lines. These compounds displayed IC_50_ values ranging from 3.58 to 15.36 µM. Concerning the IC_50_ values against HePG-2, compounds **4f** and **4r**, with IC_50_ of 5.05 and 8.10 µM, in turn, conveyed solid cytotoxic activity, which is higher than that of the reference drug sorafenib with IC_50_ of 9.18 µM. In addition, the candidates **4a** and **4l** displayed substantial anticancer potencies against HePG-2, with IC_50_ values of 12.88 and 15.36 µM, respectively. 

Regarding the HCT-116 cell lines, compound **4f** demonstrated the highest level of inhibitory action, with an IC_50_ of 6.21 µM, almost equivalent to that of the standard medicine sorafenib (IC_50_ of 5.47 µM). In addition, compounds **4a** and **4r** exhibited highly potent cytotoxic effects against HCT-116, with IC_50_ values of 9.94 and 7.81 µM, respectively. In addition, compounds **4g** and **4l** exhibited significant cytotoxicity, with IC_50_ values of 19.60 and 13.54 µM, respectively. The IC_50_ values of compounds **4a**, **4f**, **4g**, **4l**, and **4r** against the breast MCF-7 cell lines revealed their remarkable cytotoxic activity, with IC_50_ values of 5.91, 2.74, 16.46 9.46, and 3.85 µM, respectively. Meanwhile, compounds **4a**, **4f,** and **4r** possessed superior activity compared to the other tested compounds and the positive control. 

The cytotoxic activity of the produced derivatives was assessed by evaluating their IC_50_ values to establish the structure–activity relationship (SAR). The tested compounds were categorized into three groups depending on the substitution at position 6 of the benzothiazole core: 6-unsubstituted analogues 4a-f, 6-methyl analogues 4g-l, and 6-chloro analogues **4m**–**4r**. In general, the SAR study pointed out two significant characteristics. Firstly, the 6-unsubstituted benzothiazole analogues exerted an overall better cytotoxic effect than the other substituted analogues. Secondly, the cytotoxic activity was affected by different substitutions on the aromatic ring attached to the urea moiety. For example, incorporating the hydrophobic tail from sorafenib (3-chloro-4-trifluoromethyl phenyl moiety) into the structures of the target compounds, regardless of their group, yielded optimal activity and resulted in highly potent anticancer candidates. For instance, 6-unsubstituted analogue **4f** with an IC_50_ range of 2.74–5.05 µM, 6-methyl analogue 4l with an IC_50_ range of 9.46–15.36 µM, and 6-chloro analogue **4r** with an IC_50_ range of 3.85–8.10 µM. 

Regarding the 6-unsubstituted analogues **4a**–**f**, incorporating the unsubstituted phenyl ring in an aryl urea moiety, as in compound **4a** (IC_50_ = 5.91–12.88 µM), highly enhanced the anticancer activity. The inclusion of an EWD group like 4-Chloro (**4d**, IC_50_ = 30.20–33.64 µM) negatively affected the anticancer activity, causing it to be moderate, while 4-fluoro substitution (**4e**, IC_50_ = 63.39–82.45 µM) dramatically decreased the anticancer activity. Similarly, the presence of EDG-like 4-methyl (**4b**, IC_50_ = 46.53–61.78 µM) and 4-methoxy (**4c**, IC_50_ = 42.22–65.48 µM) exerted a pattern of moderate to weak activity. 

For the 6-methyl analogues **4g**–**l,** a similar effect was observed where the presence of unsubstituted phenyl fragment, as in compound **4g** (IC_50_ = 16.46–22.93 µM), resulted in enhanced activity rather than other candidates with 4-methyl (**4h**, IC_50_ = 37.36–56.22 µM), 4-methoxy (**4i**, IC_50_ = 53.10–76.69 µM), 4-chloro (**4j**, IC_50_ = 78.13–>100 µM), and 4-fluoro (**4c**, IC_50_ = 64.23–92.02 µM). 

Concerning the 6-chloro analogues **4m**–**4r**, the incorporation of unsubstituted phenyl or 4-fluorophenyl fragments to a urea moiety, as in **4m** (IC_50_ = 28.93–34.39 µM) and **4q** (IC_50_ = 21.72–27.38 µM), respectively, improved the activity compared to other candidates with different aryl urea moieties. 

#### 2.3.2. In Vitro Cytotoxicity against Normal Human Cells 

The safety profile of all the newly developed derivatives was evaluated by examining their cytotoxic impact on WI-38 cells, which are normal lung cell lines. Based on the IC_50_ values in Table 1, all the drugs exhibited favorable selectivity by showing moderate to weak cytotoxicity against normal WI-38 cells. Specifically, our most potent cytotoxic substitutes, **4a**, **4f**, **4l**, and **4r**, exhibited significantly higher IC_50_ values in relation to WI-38 cells compared to cancer cell lines. The IC_50_ values of these substitutes were 66.22, 41.24, 47.54, and 38.77 μM, respectively. This indicates that these newly developed compounds have a similar level of selectivity and therapeutic safety to the reference sorafenib (IC_50_ = 10.65 μM).

#### 2.3.3. In Vitro VEGFR-2 and BRAF (V600E) Kinases Inhibitory Assay

Derivatives **4a**, **4f**, and **4r** exhibited encouraging anticancer efficacy, prompting a subsequent evaluation of their inhibitory impact on VEGFR-2 and BRAF. The IC_50_ was calculated from the dose–response curve for different concentrations of the compounds, as shown in Table 2, using sorafenib as a positive drug control. Compound **4f** potently inhibited BRAF, with an IC_50_ of 0.194 μM, compared to sorafenib (IC_50_ of 0.171 μM). Simultaneously, it suppressed VEGFR-2, with an IC_50_ value of 0.071 μM, similar to sorafenib’s IC_50_ value of 0.069 μM. On the other hand, derivatives **4a** and **4r** revealed a lower inhibitory activity against VEGR-2 and BRAF, with IC_50_ values of 0.342 and 0.677 for compound **4a** and 0.794 and 1.359 for compound **4r**, respectively. 

#### 2.3.4. Cell Cycle Analysis

**Figure 4:**
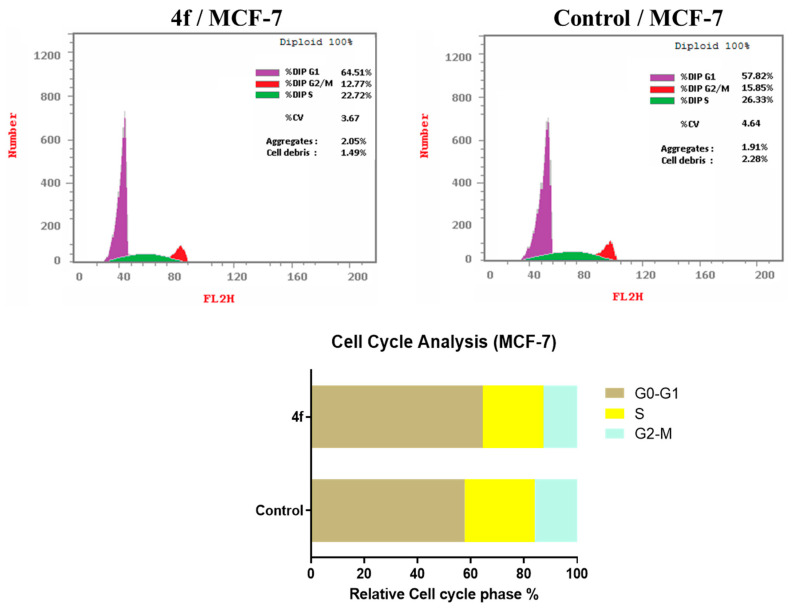
Compound **4f** affects the cell cycle of MCF-7, as shown by flow cytometric analysis.

To ascertain the precise cell cycle phase at which our highly promising chemical **4f** causes the MCF7 breast cancer cells to cease their progression, we conducted cell cycle analysis and apoptotic experiments utilizing DNA flow cytometry. Analysis of the findings (Table 3, Figure 4) revealed that compound **4f** induced cell cycle arrest in the G0-G1 phase of MCF-7 cells. This was demonstrated by an observed rise in the cell population in G0-G1 from 57.82% to 64.51% compared to untreated cells. Simultaneously, compound **4f** decreased the number of cells in the S and G2-M phases from 26.33% and 15.85% in untreated cells to 22.72% and 12.77% in treated cells, respectively. 

#### 2.3.5. Detection of Apoptosis 

**Figure 5:**
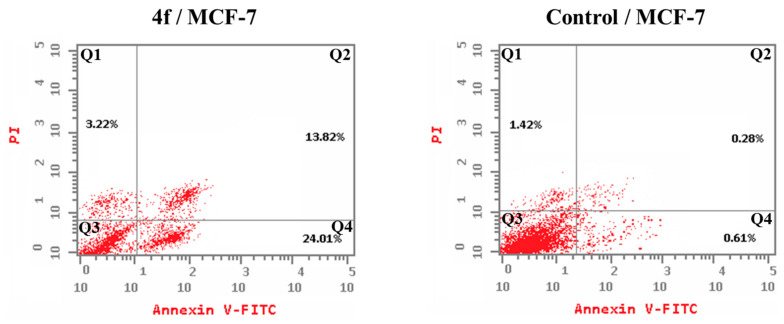
The impact of compound **4f** on the % annexin V-FITC-positive stained MCF-7 cells. Quadrant 1 indicates necrotic cells; Quadrant 2 denotes late apoptosis; Quadrant 3 shows live cells; and Quadrant 4 indicates early apoptosis.

Exposure of MCF-7 breast cancer cells to compound **4f** for 24 h resulted in the initiation of both early and late apoptosis, as well as necrosis. Figure 5 shows a noticeable increase in apoptotic cells (both early and late stages) compared to the untreated control cells. Compound **4f** resulted in a significant rise in the proportion of apoptotic cells to 37.83%, in contrast to only 0.89% in the untreated control cells. This demonstrates its capacity to suppress cell growth by triggering programmed cell death, known as apoptosis.

### 2.4. Molecular-Docking Study

Compounds **4a**, **4f**, and **4r** exhibited the most potent cytotoxic activity among the synthesized compounds, Moreover, they achieved remarkable enzyme inhibition against VEGFR-2. Previous studies indicated the ability of in silico tools to identify the binding mode of small molecules in biological systems [44,45,46,47,48]. Therefore, molecular docking was used to determine the molecular interaction between the most active drugs and the targets being studied. The binding energy of the three compounds was lower than that of the conventional inhibitor sorafenib, which is consistent with the experimental enzyme inhibition assay results. Still, compounds **4a** and **4f** demonstrated better affinity than **4r,** as presented in Table 4.

To validate our molecular-modeling methodology, the RMSD value of the docked pose of the co-crystallized ligand was assessed to be less than 1.5 Å. In the VEGFR-2 case, PyRX 0.8 software could reproduce a pose that closely matched the experimentally determined crystal structure, with an RMSD of 0.5 Å. This redocked pose maintained the fundamental interactions observed in the crystal structure, including hydrogen bonding between the core heteroaromatic system and the catalytic Cys919 residue of the hinge region. 

Proper positioning within this ATP-binding pocket, enabled by hydrogen bonds with the catalytic dyad residues, is critical for kinase inhibitory activity. Additionally, the terminal aryl moiety of sorafenib projected into the hydrophobic back pocket, enabling favorable hydrophobic contact. By reproducing these known binding interactions, our computational approach demonstrated its ability to reliably predict inhibitor–kinase binding modes. This supports its use in elucidating the structural basis for the activity of our amino-benzothiazole derivatives as novel dual VEGFR-2/BRAF inhibitors.

In this context, compound **4f** showed a similar interaction profile as sorafenib, interacting with VAL848, ALA866, VAL916, LEU1035, CYS1045, and PHE1047 in the ATP active site. Also, it maintained the interaction with LYS868, GLU885, and ASP1046 by forming a hydrogen bond in the hinge region. Finally, the aryl moiety of **4f** was able to interact with ARG1027, ASP1028, and PRO1068 in the hydrophobic pocket, which explains the better enzyme inhibition effect of this compound over the other derivatives. 

In contrast to sorafenib, our derivatives **4a** and **4r** did not fully replicate these fundamental binding interactions. Compound **4a** maintained hydrogen bonding with GLU885 and ASP1046 through its amino-benzothiazole core. However, it adopted an inverted orientation that positioned this moiety in the hydrophobic pocket rather than the ATP site. This flipped pose would disrupt the critical hinge region binding, explaining the reduced affinity of **4a**. Similarly, compound **4r** could only engage in hydrophobic contact with residues Val898, Val899, Leu1019, and Ile1044 through its benzothiazole ring. 

**Figure 6:**
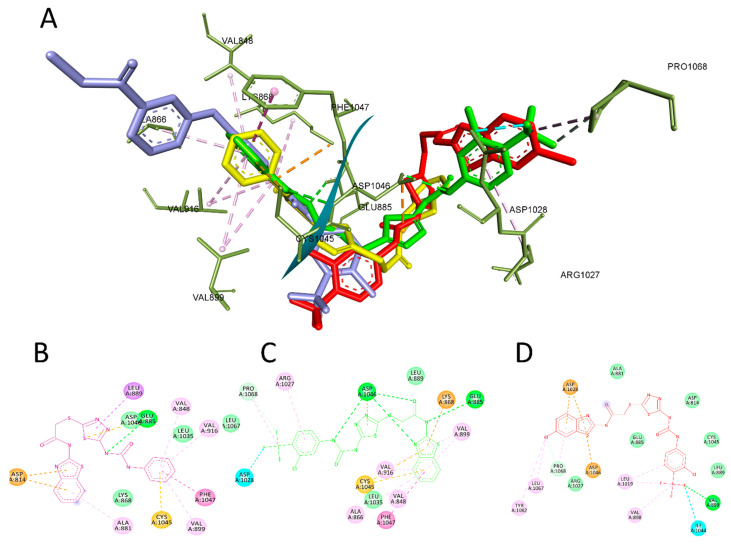
The most active compounds docked in the binding site of VEGFR-2 PDB:4ASD. (**A**) Three-dimensional interaction of compound **4a** (yellow), **4f** (green), **4r** (red), and sorafenib(cyan) with the binding site of VEFR-2. (**B**) Two-dimensional presentation of the interaction of compound **4a**. (**C**) Two-dimensional presentation of the interaction of compound **4f**. (**D**) Two-dimensional presentation of the interaction of compound **4r**.

The lack of hydrogen-bonding interactions with the hinge region residues likely contributed to its decreased potency. While both inhibitors retained some favorable protein contacts, their altered binding modes could not effectively mimic sorafenib’s interaction with the ATP site. This highlights the importance of proper positioning to achieve potent VEGFR-2 inhibition through anchoring to the catalytic cysteine and neighboring residues. Still, this was compensated for by the ability of the aryl moiety to interact with ARG1027, ASP1028, ASP1046, LEU1067, PRO1068, and TYR1082, but it failed to interact with GLU885 and ASP1046, which is critical to achieving potent enzyme inhibition activity. The interaction between compounds **4a**, **4f,** and **4r** with VEGFR-2 is depicted in Figure 6.

In the case of BRAF, the docked pose of the co-crystallized ligand showed RMSD = 0.8. The most highlighted interactions with the active site were with PHE583, CYS532, LYS483, PHE595, and ASP594 through hydrogen bonding and with VAL471, LEU514, ALA481, LEU505, and THR529 through hydrophobic interactions.

Again, compound **4f** achieved the best affinity compared to dabrafenib, as shown in Table 4. This could be attributed to its binding with ASN580 through hydrogen bonding and with CYS532, TRP531, PHE583, THR529, VAL471, ALA481, and LEU514 through hydrophobic interactions, indicating that compound **4f** has a similar binding mode to dabrafenib. In the case of compound **4r**, it interacted with SER536 through hydrogen bonding and GLY534, TYR538, PHE583, CYS532, LYS483, LEU505, ILE527, and ASP594 through hydrophobic interactions.

**Figure 7:**
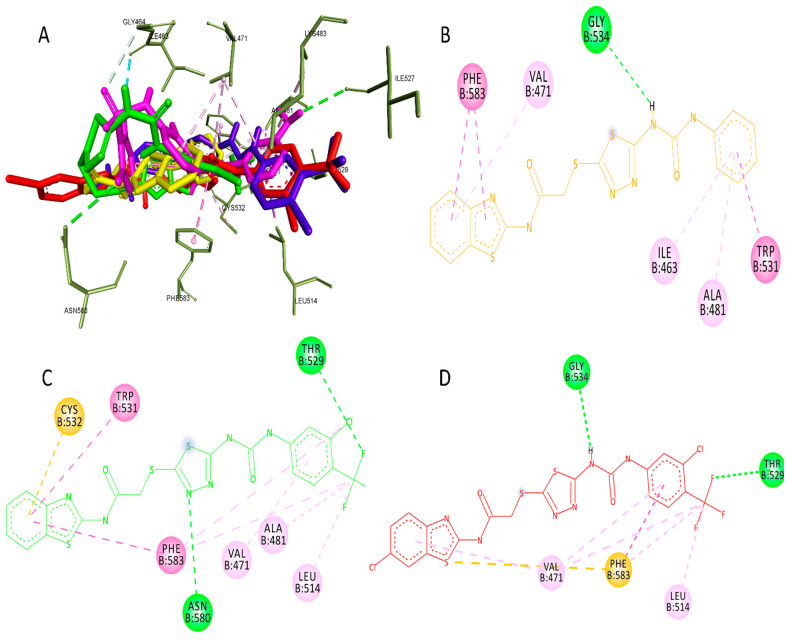
The most active compounds docked in the binding site of BRAF PDB:4XV2. (**A**) Three-dimensional interaction of compound **4a** (Yellow), **4f** (Green), **4r** (Red), sorafenib (Cyan), and dabrafenib (Magentas) with the binding site of BRAF (**B**) Two-dimensional presentation of the interaction of compound **4a**. (**C**) Two-dimensional presentation of the interaction of compound **4f**. (**D**) Two-dimensional presentation of the interaction of compound **4r**.

On the other hand, compound **4a** exerted interaction with GLY534 through hydrogen bonding and with VAL471, LEU514, THR529, and PHE583 through hydrophobic interactions, but it did not interact with CYS532, which is essential to achieve suitable inhibitory concentrations [49]. Figure 7 shows the binding of compounds **4a**, **4f,** and **4r** in the active site of BRAF. 

## 3. Experimental 

### 3.1. Chemistry

The compounds’ melting points (°C) were calculated using the Stuart apparatus (SMP 30) (Cole-Parmer, Cambridgeshire, UK). FT-IR spectroscopy was performed on KBr samples using an FT-IR 200 spectrophotometer (Thermo Fisher Scientific, Waltham, MA, USA) (in units of reciprocal centimeters) at the Faculty of Pharmacy, Mansoura University, Egypt. The ^1^H-NMR (400 MHz) and ^13^C-NMR (100 MHz) spectroscopy experiments (Bruker Avance III 400 spectrometer, Billerica, MA, USA) were conducted at the NMR Unit, Faculty of Pharmacy, Mansoura University, Egypt. TMS was used as an internal standard, and DMSO-*d*_6_ was used as the solvent. The mass spectra were obtained using a Thermo Scientific GC/MS model ISQ (Thermo Fisher Scientific, Waltham, MA, USA) at the Regional Center for Mycology and Biotechnology, Al-Azhar University, Egypt. For the Gas Chromatography–Mass Spectrometry (GC/MS) analysis, Electron Ionization (EI) was employed in full-scan mode. The mass spectrometer operates over a mass-to-charge (*m*/*z*) range of 40–1000, with an electron energy set at 70 electron volts (eV). Microanalyses were performed at the University of Cairo on a PerkinElmer 240 elemental analyzer (PerkinElmer, Waltham, MA, USA) for elements C, H, and N, and the results were recorded within the accepted limits. The chemicals and reagents utilized were procured from Aldrich Chemicals Co, Milwaukee, WI, USA, and other commercial suppliers. The reaction durations were determined using thin-layer chromatography (TLC) on a silica gel plate 60F245 E. (Merck KGaA, Darmstadt, Germany) The eluting system used was a mixture of hexane and ethyl acetate at a ratio of 2:1. The spots were seen using ultraviolet (UV) light with a wavelength range of 366–245nm. The essential precursors, namely thiadiazole chlorides and thiol derivatives (**3a**–**f**), can be readily synthesized using the established methods reported in the literature [42].

#### 3.1.1. General Procedure for Synthesis of *N*-Substituted-2-chloroacetamides (**2a**–**c**)

To a stirred solution of an appropriate amine (20 mmol, 1 eq.) in dichloromethane (50 mL) was added triethylamine (24 mmol, 1.2 eq.), and the solution was stirred in an ice-water bath for 5 min. After chloroacetyl chloride (24 mmol, 1.2 eq.) was added dropwise at 0–5 °C, the reaction mixture was stirred at room temperature for 5 h. Then, the reaction was evaporated under reduced pressure and washed with water, and a precipitate was obtained after filtering. Recrystallisation from acetone gave the *N*-substituted-2-chloroacetamides.

#### 3.1.2. General Procedure for Synthesis of Intermediates (**3a**–**f**)

*N*-substituted-2-chloroacetamide (**2a**–**c**) (200 mg, 1.5 mmol) was dissolved in acetonitrile and stirred at rt. Then, an appropriate phenyl isocyanate derivative (1.5 mmol) was added, and the stirring was allowed to continue overnight. The white cake formed in the vial was kept in a vacuum oven for 2 h to provide the corresponding urea (**3a**–**d**). The product was used for the next step without any further purification.

#### 3.1.3. General Procedure for Synthesis of 1,3,4-Thiadiazol-2-yl)thio)acetamides (**4a**–**r**)

A solution containing benzothiazole chloroacetamide derivatives **2a**–**c** (0.2 mmol), thiols **3a**–**f** (1.2 equivalents), and K_2_CO_3_ in anhydrous acetone (2 mL) was agitated at 40 °C for the duration of one night. Subsequently, the reaction mixture was transferred into a mix of ice and water, forming precipitates. These residues were then separated by filtration, washed with water, and purified through ethyl acetate recrystallisation. As a result, the desired products, specifically **4a**–**r**, were obtained.

*N-(Benzo[d]thiazol-2-yl)-2-((5-(3-phenylureido)-1,3,4-thiadiazol-2-yl)thio)acetamide* (**4a**)
White solid (0.07 g, 79%). M.p. 273–275 °C. IR (*ν*max/cm^−1^): 3345, 3170, 3050, 2920, 1683, 1603, 1572, 1240. ^1^H NMR (400 MHz, DMSO-*d*_6_) δ 12.71 (s, 1H, NH; D_2_O exchangeable), 11.18 (s, 1H, NH; D_2_O exchangeable), 9.18 (s, 1H, NH; D_2_O exchangeable), 8.00 (d, *J* = 7.8 Hz, 1H, Ar-H), 7.79 (d, *J* = 7.8 Hz, 1H, Ar-H), 7.51–7.44 (m, 3H, Ar-H), 7.40–7.28 (m, 3H, Ar-H), 7.06 (t, *J* = 7.3 Hz, 1H, Ar-H), 4.35 (s, 2H, SCH_2_).^13^C NMR (101 MHz, DMSO-*d*_6_) δ 172.8 (CO), 167.6 (CO), 161.5, 158.2, 154.3, 148.9, 138.7, 131.9, 129.4, 126.7, 124.2, 123.6, 122.3, 121.2, 119.4, 37.4 (CH_2_). Gas Chromatography–Mass Spectrometry Electron Ionization (EI) *m*/*z* calculated for [M^+^] C_18_H_14_N_6_O_2_S_3_ = 442.54, found 442.49 (mass error ∆m = −0.05 ppm. Anal. Calcd for C_18_H_14_N_6_O_2_S_3_ (442.54): C, 48.85; H, 3.19; N, 18.99. Found: C, 48.80; H, 3.05; N, 19.06%.
*N-(Benzo[d]thiazol-2-yl)-2-((5-(3-(p-tolyl)ureido)-1,3,4-thiadiazol-2-yl)thio)acetamide* (**4b**)
White solid (0.073 g, 80%). M.p. 270–272 °C. IR (*ν*max/cm^−1^): 3340, 3180, 3050, 2930, 1682, 1605, 1540, 1170. ^1^H NMR (400 MHz, DMSO-*d*_6_) δ 12.69 (s, 1H, NH; D_2_O exchangeable), 11.15 (s, 1H, NH; D_2_O exchangeable), 9.13 (s, 1H, NH; D_2_O exchangeable), 8.16–7.72 (m, 2H, Ar-H), 7.61–6.96 (m, 6H, Ar-H), 4.35 (s, 2H, SCH_2_), 2.26 (s, 3H, CH_3_).^13^C NMR (101 MHz, DMSO-*d*_6_) δ 172.5 (CO), 167.7 (CO), 161.7, 158.3, 151.3, 149.0, 139.3, 136.3, 132.0, 129.8, 126.7, 124.2, 122.3, 121.2, 119.4, 37.6 (CH_2_), 21.0 (CH_3_). GC-MS EI *m*/*z* calculated for [M^+^] C_19_H_16_N_6_O_2_S_3_ = 456.56, found 456.66 (mass error ∆m = −0.1 ppm. Anal. Calcd for C_19_H_16_N_6_O_2_S_3_ (456.56): C, 49.98; H, 3.53; N, 18.41. Found: C, 49.91; H, 3.55; N, 18.33%.
*N-(Benzo[d]thiazol-2-yl)-2-((5-(3-(4-methoxyphenyl)ureido)-1,3,4-thiadiazol-2-yl)thio)acetamide* (**4c**)
White solid (0.068 g, 72%). M.p. 262–264 °C. IR (*ν*max/cm^−1^): 3345, 3182, 3050, 2930, 1680, 1605, 1540, 1175. ^1^H NMR (400 MHz, DMSO-*d*_6_) δ 12.71 (s, 1H, NH; D_2_O exchangeable), 11.17 (s, 1H, NH; D_2_O exchangeable), 9.02 (s, 1H, NH; D_2_O exchangeable), 8.00 (d, *J* = 7.8 Hz, 1H, Ar-H), 7.79 (d, *J* = 7.8 Hz, 1H, Ar-H), 7.47 (t, *J* = 7.7 Hz, 1H, Ar-H), 7.40 (d, *J* = 8.8 Hz, 2H, Ar-H), 7.34 (t, *J* = 7.7 Hz, 1H, Ar-H), 6.90 (d, *J* = 8.8 Hz, 2H, Ar-H), 4.34 (s, 2H, SCH_2_), 3.73 (s, 3H, OCH_3_).^13^C NMR (101 MHz, DMSO-*d*_6_) δ 174.3 (CO), 167.0 (CO), 158.3, 156.4, 152.3, 149.3, 139.3, 131.8, 131.6, 126.8, 124.3, 122.3, 121.4, 121.1, 114.6, 55.7 (OCH_3_), 37.0 (CH_2_). GC-MS EI *m*/*z* calculated for [M^+^] C_19_H_16_N_6_O_3_S_3_ = 472.56, found 472.43 (mass error ∆m = −0.13 ppm. Anal. Calcd for C_19_H_16_N_6_O_3_S_3_ (472.56): C, 48.29; H, 3.41; N, 17.78. Found: C, 48.35; H, 3.44; N, 17.80%.
*N-(Benzo[d]thiazol-2-yl)-2-((5-(3-(4-chlorophenyl)ureido)-1,3,4-thiadiazol-2-yl)thio)acetamide* (**4d**)
White solid (0.062 g, 65%). M.p. 275–277 °C. IR (*ν*max/cm^−1^): 3340, 3180, 3050, 2930, 1680, 1605, 1540, 1170. ^1^H NMR (400 MHz, DMSO-*d*_6_) δ 12.70 (s, 1H, NH; D_2_O exchangeable), 11.34 (s, 1H, NH; D_2_O exchangeable), 9.47 (s, 1H, NH; D_2_O exchangeable), 8.00 (d, *J* = 7.6 Hz, 1H, Ar-H), 7.78 (d, *J* = 7.6 Hz, 1H, Ar-H), 7.55 (d, *J* = 7.5 Hz, 2H, Ar-H), 7.50–7.43 (m, 1H, Ar-H), 7.39–7.27 (m, 3H, Ar-H), 4.34 (s, 2H, SCH_2_).^13^C NMR (101 MHz, DMSO-*d*_6_) δ 174.1 (CO), 167.3 (CO), 158.3, 156.6, 152.0, 148.7, 137.9, 131.9, 129.2, 127.1, 126.7, 124.2, 122.3, 121.4, 121.0, 37.6 (CH_2_). GC-MS EI *m*/*z* calculated for [M^+^] C_18_H_13_ClN_6_O_2_S_3_ = 476.98, found 476.97 (mass error ∆m = −0.01 ppm. Anal. Calcd for C_18_H_13_ClN_6_O_2_S_3_ (476.98): C, 45.33; H, 2.75; N, 17.62. Found: C, 45.32; H, 2.79; N, 17.47%.
*N-(Benzo[d]thiazol-2-yl)-2-((5-(3-(4-fluorophenyl)ureido)-1,3,4-thiadiazol-2-yl)thio)acetamide* (**4e**)
White solid (0.06 g, 65%). M.p. 268–270 °C. IR (*ν*max/cm^−1^): 3340, 3185, 3050, 2930, 1690, 1605, 1545, 1172. ^1^H NMR (400 MHz, DMSO-*d*_6_) δ 12.64 (s, 1H, NH; D_2_O exchangeable), 11.46 (s, 1H, NH; D_2_O exchangeable), 9.40 (s, 1H, NH; D_2_O exchangeable), 7.99 (d, *J* = 6.9 Hz, 1H, Ar-H), 7.78 (d, *J* = 6.9 Hz, 1H, Ar-H), 7.61–7.41 (m, 3H, Ar-H), 7.36–7.23 (m, 1H, Ar-H), 7.21–7.06 (m, 2H, Ar-H), 4.35 (s, 2H, SCH_2_).^13^C NMR (101 MHz, DMSO-*d*_6_) δ 167.7 (CO), 159.6 (CO), 158.3, 157.6 *J*(F,C) = 238 Hz, 152.3, 148.9, 136.0, 131.9, 126.7, 124.2, 122.3, 121.3 *J*(F,C) = 7 Hz, 121.1, 116.0, 115.8 *J*(F,C) = 22 Hz, 36.6 (CH_2_). GC-MS EI *m*/*z* calculated for [M^+^] C_18_H_13_FN_6_O_2_S_3_ = 460.53, found 460.44 (mass error ∆m = −0.09 ppm. Anal. Calcd for C_18_H_13_FN_6_O_2_S_3_ (460.53): C, 46.94; H, 2.85; N, 18.25. Found: C, 46.98; H, 2.80; N, 18.12%.
*N-(Benzo[d]thiazol-2-yl)-2-((5-(3-(3-chloro-4-(trifluoromethyl)phenyl)ureido)-1,3,4-thiadiazol-2-yl)thio)acetamide* (**4f**)
White solid (0.069 g, 63%). M.p. 259–261 °C. IR (*ν*max/cm^−1^): 3345, 3180, 3050, 2930, 1695, 1605, 1545, 1170.^1^H NMR (400 MHz, DMSO-*d*_6_) δ 12.72 (s, 1H, NH; D_2_O exchangeable), 11.77 (s, 1H, NH; D_2_O exchangeable), 9.88 (s, 1H, NH; D_2_O exchangeable), 8.16 (s, 1H, Ar-H), 8.00 (d, *J* = 8.1 Hz, 1H, Ar-H), 7.78 (d, *J* = 7.8 Hz, 2H, Ar-H), 7.65 (d, *J* = 7.7 Hz, 1H, Ar-H), 7.46 (d, *J* = 7.7 Hz, 1H, Ar-H), 7.34 (d, *J* = 7.8 Hz, 1H, Ar-H), 4.35 (s, 2H, SCH_2_).^13^C NMR (101 MHz, DMSO-*d*_6_) δ 168.0 (CO), 163.0 (CO), 157.0, 154.6, 152.3, 149.3, 136.9, 132.3, 127.9, 124.2, 122.3, 120.9, 118.9, 118.0, 117.8, 36.6 (CH_2_). GC-MS EI *m*/*z* calculated for [M^+^] C_19_H_12_ClF_3_N_6_O_2_S_3_ = 544.98, found 544.85 (mass error ∆m = −0.13 ppm. Anal. Calcd for C_19_H_12_ClF_3_N_6_O_2_S_3_ (544.98): C, 41.87; H, 2.22; N, 15.42. Found: C, 41.72; H, 2.20; N, 15.48%.
*N-(6-Methylbenzo[d]thiazol-2-yl)-2-((5-(3-phenylureido)-1,3,4-thiadiazol-2-yl)thio)acetamide* (**4g**)
White solid (0.074 g, 81%). M.p. 263–265 °C. IR (*ν*max/cm^−1^): 3301, 3113, 3042, 2920, 1684, 1605, 1540, 1240. ^1^H NMR (400 MHz, DMSO-*d*_6_) δ 12.62 (s, 1H, NH; D_2_O exchangeable), 11.16 (s, 1H, NH; D_2_O exchangeable), 9.16 (s, 1H, NH; D_2_O exchangeable), 7.78 (s, 1H, Ar-H), 7.66 (d, *J* = 7.3 Hz, 1H, Ar-H), 7.57–7.43 (m, 2H, Ar-H), 7.39–7.24 (m, 3H, Ar-H), 7.12–7.01 (m, 1H, Ar-H), 4.33 (s, 2H, SCH_2_), 2.42 (s, 3H, CH_3_).^13^C NMR (101 MHz, DMSO-*d*_6_) δ 167.7 (CO), 157.3 (CO), 156.8, 154.3, 152.0, 147.2, 138.7, 133.7, 132.1, 129.4, 128.0, 123.6, 122.1, 121.3, 119.4, 37.4 (CH_2_), 21.5 (CH_3_). GC-MS EI *m*/*z* calculated for [M^+^] C_19_H_16_N_6_O_2_S_3_ = 456.56, found 456.44 (mass error ∆m = −0.12 ppm. Anal. Calcd for C_19_H_16_N_6_O_2_S_3_ (456.56): C, 49.98; H, 3.53; N, 18.41. Found: C, 49.88; H, 3.50; N, 18.49%.
*N-(6-Methylbenzo[d]thiazol-2-yl)-2-((5-(3-(p-tolyl)ureido)-1,3,4-thiadiazol-2-yl)thio)acetamide* (**4h**)
White solid (0.071 g, 79%). M.p. 265–267 °C. IR (*ν*max/cm^−1^): 3345, 3182, 3050, 2935, 1680, 1605, 1545, 1170. ^1^H NMR (400 MHz, DMSO-*d*_6_) δ 12.62 (s, 1H, NH; D_2_O exchangeable), 11.12 (s, 1H, NH; D_2_O exchangeable), 9.09 (s, 1H, NH; D_2_O exchangeable), 7.78 (s, 1H, Ar-H), 7.66 (d, *J* = 8.0 Hz, 1H, Ar-H), 7.37 (d, *J* = 7.4 Hz, 2H, Ar-H), 7.27 (d, *J* = 7.5 Hz, 1H, Ar-H), 7.12 (d, *J* = 7.4 Hz, 2H, Ar-H), 4.32 (s, 2H, SCH_2_), 2.42 (s, 3H, CH_3_), 2.25 (s, 3H, CH_3_).^13^C NMR (101 MHz, DMSO-*d*_6_) δ 167.6 (CO), 158.1 (CO), 157.5, 154.7, 152.5, 147.2, 138.2, 133.7, 132.8, 132.3, 129.8, 128.0, 121.8, 120.8, 119.4, 37.4 (CH_2_), 21.5 (CH_3_), 20.8. GC-MS EI *m*/*z* calculated for [M^+^] C_20_H_18_N_6_O_2_S_3_ = 470.59, found 470.62 (mass error ∆m = −0.03 ppm. Anal. Calcd for C_20_H_18_N_6_O_2_S_3_ (470.59): C, 51.05; H, 3.86; N, 17.86. Found: C, 51.14; H, 3.81; N, 17.96%.
*2-((5-(3-(4-Methoxyphenyl)ureido)-1,3,4-thiadiazol-2-yl)thio)-N-(6-methylbenzo[d]thiazol-2-yl)acetamide* (**4i**)
White solid (0.068 g, 70%). M.p. 264–266 °C. IR (*ν*max/cm^−1^): 3300, 3180, 3050, 2930, 1681, 1605, 1550, 1170. ^1^H NMR (400 MHz, DMSO-*d*_6_) δ 12.61 (s, 1H, NH; D_2_O exchangeable), 11.07 (s, 1H, NH; D_2_O exchangeable), 8.95 (s, 1H, NH; D_2_O exchangeable), 7.78 (s, 1H, Ar-H), 7.66 (d, *J* = 8.0 Hz, 1H, Ar-H), 7.38 (d, *J* = 8.4 Hz, 2H, Ar-H), 7.28 (d, *J* = 8.0 Hz, 1H, Ar-H), 6.90 (d, *J* = 8.4 Hz, 2H, Ar-H), 4.32 (s, 2H, SCH_2_), 3.73 (s, 3H, OCH_3_), 2.42 (s, 3H, CH_3_).^13^C NMR (101 MHz, DMSO-*d*_6_) δ 167.6 (CO), 161.5 (CO), 157.8, 155.8, 151.9, 147.2, 139.9, 133.7, 132.1, 131.6, 128.0, 121.8, 121.3, 120.8, 114.6, 55.7 (OCH_3_), 37.4 (CH_2_), 21.5. GC-MS EI *m*/*z* calculated for [M^+^] C_20_H_18_N_6_O_3_S_3_ = 486.59, found 486.38 (mass error ∆m = −0.21 ppm. Anal. Calcd for C_20_H_18_N_6_O_3_S_3_ (486.59): C, 49.37; H, 3.73; N, 17.27. Found: C, 49.51; H, 3.66; N, 17.20%.
*2-((5-(3-(4-Chlorophenyl)ureido)-1,3,4-thiadiazol-2-yl)thio)-N-(6-methylbenzo[d]thiazol-2-yl)acetamide* (**4j**)
White solid (0.07 g, 71%). M.p. 261–263 °C. IR (*ν*max/cm^−1^): 3340, 3175, 3050, 2925, 1684, 1610, 1570, 1170. ^1^H NMR (400 MHz, DMSO-*d*_6_) δ 12.64 (s, 1H, NH; D_2_O exchangeable), 11.18 (s, 1H, NH; D_2_O exchangeable), 9.49 (s, 1H, NH; D_2_O exchangeable), 7.78 (s, 1H, Ar-H), 7.66 (s, 1H, Ar-H), 7.55 (s, 1H, Ar-H), 7.45–7.17 (m, 4H, Ar-H), 4.34 (s, 2H, SCH_2_), 2.42 (s, 3H, CH_3_).^13^C NMR (101 MHz, DMSO-*d*_6_) δ 167.6 (CO), 157.3 (CO), 156.3, 152.5, 146.9, 139.7, 133.7, 132.1, 129.2, 128.9, 128.0, 127.6, 121.8, 120.9, 120.8, 37.3 (CH_2_), 21.6 (CH_3_). GC-MS EI *m*/*z* calculated for [M^+^] C_19_H_15_ClN_6_O_2_S_3_ = 491.01, found 490.92 (mass error ∆m = −0.09 ppm. Anal. Calcd for C_19_H_15_ClN_6_O_2_S_3_ (491.01): C, 46.48; H, 3.08; N, 17.12. Found: C, 46.43; H, 3.09; N, 17.05%.
*2-((5-(3-(4-Fluorophenyl)ureido)-1,3,4-thiadiazol-2-yl)thio)-N-(6-methylbenzo[d]thiazol-2-yl)acetamide* (**4k**)
White solid (0.071 g, 75%). M.p. 250–252 °C. IR (*ν*max/cm^−1^): 3340, 3180, 3050, 2930, 1680, 1605, 1545, 1170. ^1^H NMR (400 MHz, DMSO-*d*_6_) δ 12.60 (s, 1H, NH; D_2_O exchangeable), 12.40 (s, 1H, NH; D_2_O exchangeable), 10.56 (s, 1H, NH; D_2_O exchangeable), 7.77 (s, 1H, Ar-H), 7.72–6.62 (m, 3H, Ar-H), 7.27 (d, *J* = 7.9 Hz, 1H, Ar-H), 7.09 (t, *J* = 8.8 Hz, 2H, Ar-H), 4.30 (s, 2H, SCH_2_), 2.42 (s, 3H, CH_3_).^13^C NMR (101 MHz, DMSO-*d*_6_) δ 167.6 (CO), 159.2 (CO), 157.4, 156.7 *J*(F,C) = 244 Hz, 146.9, 136.5, 133.6, 132.1, 128.0, 121.8, 120.7, 120.5, 120.4 *J*(F,C) = 7 Hz, 115.8, 115.6 *J*(F,C) = 22 Hz, 37.4 (CH_2_), 21.8 (CH_3_). GC-MS EI *m*/*z* calculated for [M^+^] C_19_H_15_FN_6_O_2_S_3_ = 474.55, found 474.49 (mass error ∆m = −0.06 ppm. Anal. Calcd for C_19_H_15_FN_6_O_2_S_3_ (474.55): C, 48.09; H, 3.19; N, 17.71. Found: C, 48.17; H, 3.26; N, 17.68%.
*2-((5-(3-(3-Chloro-4-(trifluoromethyl)phenyl)ureido)-1,3,4-thiadiazol-2-yl)thio)-N-(6-methylbenzo[d]thiazol-2-yl)acetamide* (**4l**)
White solid (0.075 g, 67%). M.p. 266–268 °C. IR (*ν*max/cm^−1^): 3400, 3175, 3100, 2950, 1710, 1610, 1570, 1170. ^1^H NMR (400 MHz, DMSO-*d*_6_) δ 12.65 (s, 1H, NH; D_2_O exchangeable), 11.61 (s, 1H, NH; D_2_O exchangeable), 9.81 (s, 1H, NH; D_2_O exchangeable), 8.15 (s, 1H, Ar-H), 7.77 (d, *J* = 8.6 Hz, 2H, Ar-H), 7.66 (dd, *J* = 8.4, 3.5 Hz, 2H, Ar-H), 7.27 (d, *J* = 8.2 Hz, 1H, Ar-H), 4.34 (s, 2H, SCH_2_), 2.42 (s, 3H, CH_3_).^13^C NMR (101 MHz, DMSO-*d*_6_) δ 173.7 (CO), 167.4 (CO), 157.3, 153.8, 150.8, 146.9, 138.9, 133.7, 132.6, 132.1, 128.0, 124.2, 121.8, 120.8, 118.0, 37.3 (CH_2_), 32.4, 21.5 (CH_3_). GC-MS EI *m*/*z* calculated for [M^+^] C_20_H_14_ClF_3_N_6_O_2_S_3_ = 559.01, found 559.2 (mass error ∆m = −0.19 ppm. Anal. Calcd for C_20_H_14_ClF_3_N_6_O_2_S_3_ (559.01): C, 42.97; H, 2.52; N, 15.03. Found: C, 42.81; H, 2.56; N, 15.07%.
*N-(6-Chlorobenzo[d]thiazol-2-yl)-2-((5-(3-phenylureido)-1,3,4-thiadiazol-2-yl)thio)acetamide* (**4m**)
White solid; (0.067 g, 71%). M.p. 292–294 °C. IR (*ν*max/cm^−1^): 3345, 3185, 3050, 2930, 1685, 1605, 1545, 1170. ^1^H NMR (400 MHz, DMSO-*d*_6_) δ 12.55 (s, 1H, NH; D_2_O exchangeable), 11.50 (s, 1H, NH; D_2_O exchangeable), 9.25 (s, 1H, NH; D_2_O exchangeable), 8.15 (s, 1H, Ar-H), 7.78 (d, *J* = 8.4 Hz, 1H, Ar-H), 7.55–7.45 (m, 3H, Ar-H), 7.33 (d, *J* = 7.0 Hz, 2H, Ar-H), 7.05 (s, 1H, Ar-H), 4.34 (s, 2H, SCH_2_).^13^C NMR (101 MHz, DMSO-*d*_6_) δ 168.8 (CO), 166.7 (CO), 164.3, 157.8, 153.0, 151.1, 139.3, 133.9, 129.4, 128.2, 127.6, 123.4, 122.4, 122.0, 119.3, 37.3 (CH_2_). GC-MS EI *m*/*z* calculated for [M^+^] C_18_H_13_ClN_6_O_2_S_3_ = 476.98, found 476.75 (mass error ∆m = −0.23 ppm. Anal. Calcd for C_18_H_13_ClN_6_O_2_S_3_ (476.98): C, 45.33; H, 2.75; N, 17.62. Found: C, 45.41; H, 2.74; N, 17.49%.
*N-(6-Chlorobenzo[d]thiazol-2-yl)-2-((5-(3-(p-tolyl)ureido)-1,3,4-thiadiazol-2-yl)thio)acetamide* (**4n**)
White solid (0.072 g, 73%). M.p. 268–270 °C. IR (*ν*max/cm^−1^): 3340, 3185, 3050, 2930, 1680, 1605, 1545, 1180. ^1^H NMR (400 MHz, DMSO-*d*_6_) δ 12.85 (s, 1H, NH; D_2_O exchangeable), 11.01 (s, 1H, NH; D_2_O exchangeable), 9.01 (s, 1H, NH; D_2_O exchangeable), 8.16–8.13 (m, 1H, Ar-H), 7.78 (d, *J* = 8.6 Hz, 1H, Ar-H), 7.48 (dd, *J* = 8.6, 2.0 Hz, 1H, Ar-H), 7.36 (d, *J* = 8.1 Hz, 2H, Ar-H), 7.13 (d, *J* = 8.1 Hz, 2H, Ar-H), 4.34 (s, 2H, SCH_2_), 2.26 (s, 3H, CH_3_).^13^C NMR (101 MHz, DMSO-*d*_6_) δ 168.7 (CO), 165.3 (CO), 164.0, 159.3, 153.0, 151.9, 139.3, 133.9, 129.8, 128.2, 127.6, 123.4, 122.4, 122.0, 119.5, 37.3 (CH_2_), 20.9 (CH_3_). GC-MS EI *m*/*z* calculated for [M^+^] C_19_H_15_ClN_6_O_2_S_3_ = 491.01, found 490.88 (mass error ∆m = −0.13 ppm. Anal. Calcd for C_19_H_15_ClN_6_O_2_S_3_ (491.01): C, 46.48; H, 3.08; N, 17.12. Found: C, 46.59; H, 3.02; N, 17.17%.
*N-(6-Chlorobenzo[d]thiazol-2-yl)-2-((5-(3-(4-methoxyphenyl)ureido)-1,3,4-thiadiazol-2-yl)thio)acetamide* (**4o**)
White solid (0.075 g, 74%). M.p. 267–269 °C. IR (*ν*max/cm^−1^): 3345, 3180, 3050, 2930, 1680, 1605, 1545, 1170. ^1^H NMR (400 MHz, DMSO-*d*_6_) δ 12.68 (s, 1H, NH; D_2_O exchangeable), 11.31 (s, 1H, NH; D_2_O exchangeable), 9.01 (s, 1H, NH; D_2_O exchangeable), 8.15 (s, 1H, Ar-H), 7.78 (s, 1H, Ar-H), 7.50–7.39 (m, 3H, Ar-H), 7.02–6.98 (m, 2H, Ar-H), 4.34 (s, 2H, SCH_2_), 3.73 (s, 3H, OCH_3_).^13^C NMR (101 MHz, DMSO-*d*_6_) δ 168.7 (CO), 167.7 (CO), 159.0, 155.0, 152.7, 148.0, 139.1, 133.6, 128.2, 127.1, 122.4, 122.0, 121.3, 120.3, 114.5, 55.7 (OCH_3_), 37.3 (CH_2_). GC-MS EI *m*/*z* calculated for [M^+^] C_19_H_15_ClN_6_O_3_S_3_ = 507.01, found 507.1 (mass error ∆m = −0.09 ppm. Anal. Calcd for C_19_H_15_ClN_6_O_3_S_3_ (507.01): C, 45.01; H, 2.98; N, 16.58. Found: C, C, 45.13; H, 2.86; N, 16.61%.
*N-(6-Chlorobenzo[d]thiazol-2-yl)-2-((5-(3-(4-chlorophenyl)ureido)-1,3,4-thiadiazol-2-yl)thio)acetamide* (**4p**)
White solid (0.071 g, 70%). M.p. 275–277 °C. IR (*ν*max/cm^−1^): 3345, 3180, 3050, 2930, 1680, 1605, 1545, 1170. ^1^H NMR (400 MHz, DMSO-*d*_6_) δ 12.80 (s, 1H, NH; D_2_O exchangeable), 11.23 (s, 1H, NH; D_2_O exchangeable), 9.39 (s, 1H, NH; D_2_O exchangeable), 8.15 (d, *J* = 1.9 Hz, 1H, Ar-H), 7.78 (d, *J* = 8.6 Hz, 1H, Ar-H), 7.54 (d, *J* = 8.7 Hz, 2H, Ar-H), 7.48 (dd, *J* = 8.6, 2.1 Hz, 1H, Ar-H), 7.37 (d, *J* = 8.7 Hz, 2H, Ar-H), 4.35 (s, 2H, SCH_2_).^13^C NMR (101 MHz, DMSO-*d*_6_) δ 167.8 (CO), 161.2 (CO), 159.1, 153.4, 151.6, 147.9, 140.4, 137.8, 133.6, 129.3, 128.3, 127.1, 122.4, 122.0, 121.0, 37.6 (CH_2_). GC-MS EI *m*/*z* calculated for [M^+^] C_18_H_12_C_l2_N_6_O_2_S_3_ = 511.43, found 511.25 (mass error ∆m = −0.18 ppm. Anal. Calcd for C_18_H_12_C_l2_N_6_O_2_S_3_ (511.43): C, 42.27; H, 2.37; N, 16.43. Found: C, 42.25; H, 2.30; N, 16.49%.
*N-(6-Chlorobenzo[d]thiazol-2-yl)-2-((5-(3-(4-fluorophenyl)ureido)-1,3,4-thiadiazol-2-yl)thio)acetamide* (**4q**)
White solid (0.063 g, 64%). M.p. 259–261 °C. IR (*ν*max/cm^−1^): 3340, 3185, 3040, 2930, 1680, 1605, 1550, 1170. ^1^H NMR (400 MHz, DMSO-*d*_6_) δ 12.43 (s, 1H, NH; D_2_O exchangeable), 12.40 (s, 1H, NH; D_2_O exchangeable), 9.84 (s, 1H, NH; D_2_O exchangeable), 8.15 (s, 1H, Ar-H), 7.77 (d, *J* = 8.5 Hz, 1H, Ar-H), 7.56 (s, 2H, Ar-H), 7.48 (d, *J* = 8.3 Hz, 1H, Ar-H), 7.13 (t, *J* = 8.6 Hz, 2H, Ar-H), 4.34 (s, 2H, SCH_2_).^13^C NMR (101 MHz, DMSO-*d*_6_) δ 167.9 (CO), 159.2 (CO), 157.1 *J*(F,C) = 235 Hz, 153.9, 151.3, 147.9, 135.8, 133.6, 129.7, 128.2, 127.0, 122.3, 122.0, 121.0 *J*(F,C) = 7 Hz, 115.9 *J*(F,C) = 22 Hz, 37.6 (CH_2_). GC-MS EI *m*/*z* calculated for [M^+^] C_18_H_12_ClFN_6_O_2_S_3_ = 494.97, found 494.95 (mass error ∆m = −0.02 ppm. Anal. Calcd for C_18_H_12_ClFN_6_O_2_S_3_ (494.97): C, 43.68; H, 2.44; N, 16.98. Found: C, 43.74; H, 2.41; N, 16.84%.
*2-((5-(3-(3-Chloro-4-(trifluoromethyl)phenyl)ureido)-1,3,4-thiadiazol-2-yl)thio)-N-(6-chlorobenzo[d]thiazol-2-yl)acetamide* (**4r**)
White solid (0.08 g, 69%). M.p. 239–241 °C. IR (*ν*max/cm^−1^): 3270, 3180, 3050, 2920, 1685, 1600, 1550, 1172. ^1^H NMR (400 MHz, DMSO-*d*_6_) δ 12.87 (s, 1H, NH; D_2_O exchangeable), 10.26 (s, 1H, NH; D_2_O exchangeable), 8.34 (s, 1H, NH; D_2_O exchangeable), 8.07 (d, *J* = 1.6 Hz, 1H, Ar-H), 7.83 (d, *J* = 8.5 Hz, 1H, Ar-H), 7.71 (d, *J* = 8.6 Hz, 1H, Ar-H), 7.48 (d, *J* = 8.8 Hz, 1H, Ar-H), 7.43 (dd, *J* = 8.6, 1.9 Hz, 1H, Ar-H), 4.18 (s, 2H, SCH_2_). GC-MS EI *m*/*z* calculated for [M^+^] C_19_H_11_C_l2_F_3_N_6_O_2_S_3_ = 579.43, found 579.31 (mass error ∆m = −0.12 ppm. Anal. Calcd for C_19_H_11_C_l2_F_3_N_6_O_2_S_3_ (579.43): C, 39.38; H, 1.91; N, 14.50. Found: C, 39.31; H, 1.76; N, 14.55%.

### 3.2. Biological Evaluation

#### 3.2.1. Antiproliferative Screening 

As previously documented, the in vitro anticancer efficacy of the produced hybrids was evaluated using an MTT test [39,44]. The used cell lines was bought from Vacsera (Giza, Egypt).

#### 3.2.2. In Vitro VEGFR-2 and BRAF Inhibitory Test

The in vitro assays were carried out as previously described [50].

#### 3.2.3. Flow Cytometry Analysis of the Cell Cycle Distribution 

As previously documented, the cell cycle study was conducted on the MCF-7 cell lines, stained with PI and examined using a Calibur flow cytometer [50]. 

#### 3.2.4. Analysis of Cellular Apoptosis 

The level of apoptosis was quantified utilizing the MCF-7 cell lines and Annexin V-FITC/PI apoptosis detection kit, as documented in the literature [50]. 

### 3.3. Molecular Docking

Molecular docking was employed to examine the binding mechanism of compounds **4a**, **4f**, and **4r** regarding sorafenib. This analysis aimed to gain insights into how these compounds interact with the crucial amino acid residues in VEGFR-2 and BRAF. Hence, their crystal structures were downloaded from PDB using the codes 4ASD and 4XV2, respectively. The retrieved 3D structures were prepared using the Protein Repair and Analysis Server, where the bond orders, missing atoms, hydrogen bonds, and charges were optimized and corrected [51]. Then, water molecules and co-crystallized ligands were removed. The PDB file was loaded to PyRx software [52,53] to obtain pdbqt files and define the active site as the grid box size was 20 × 20 × 20 using this coordinate for VEGFR-2: X:−23.744, Y:−4.022, and Z: −9.684, while for BRAF, it was X: −1.784, Y: −1.287, and Z: 7.74.

The compounds (**4a**, **4f**, **4r**, sorafenib, and dabrafenib) were drawn using Marvin sketch version 21.17.0, a software developed by ChemAxon (https://www.chemaxon.com). The drawings were saved as mol files and imported into the PyRx interface. The molecular-docking study utilized Autodock Vina as the engine, employing default parameters with an exhaustiveness value of 12. Post-docking analysis involved the selection of 3 postures based on their binding score (ΔG). Finally, the docked poses were evaluated for their ability to bind with the active site using the Discovery studio visualizer, where 2D and 3D presentations of the ligand–protein complexes were generated [54,55]

## 4. Conclusions

A novel series of proposed VEGFER-2/BRAF dual inhibitors were synthesized by maintaining the pharmacophoric features of the previously reported dual inhibitors. Most of the compounds exhibited moderate to excellent cytotoxic activities toward the HePG-2, HCT-116, and MCF-7 cell lines. The most potent motifs were **4a**, **4f**, **4l**, and **4r**, which displayed promising antitumor potency. The SARs revealed that six unsubstituted benzothiazole analogues exerted an overall better cytotoxic effect than the other substituted analogues. Also, incorporating the hydrophobic tail from sorafenib (3-chloro-4-trifluoromethyl phenyl moiety) maintained the high cytotoxic activity. The four most active cytotoxic compounds, **4a**, **4f**, **4l**, and **4r**, were evaluated for their VEGFR-2/BRAF inhibitory activity. The results confirmed the capability of compound **4f** to inhibit both enzymes with a comparable IC_50_ with the reference compound. Further investigation indicated that compound **4f** arrested the cell cycle at the S and G2-M phases and induced cell apoptosis in the MCf-7 cell line. A molecular-docking study explained the high inhibitory activity of compound **4f** and the lower inhibitory activity of compounds **4a** and **4r**. Based on these findings, further structural modification of compound **4f** is proposed to optimize its VEGFER-2 and BRAF inhibitory activity.

## Figures and Tables

**Table 1:** Assessment of the antiproliferative activity (IC_50_) of the target compounds **4a**–**r** against the HepG-2, HCT-116, MCF-7, and WI-38 cell lines.

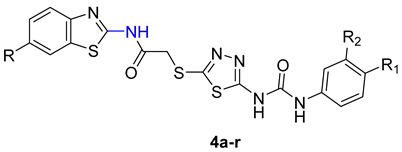							
Comp. No.	R	R_1_	R_2_	IC_50_ (µM)			
				HePG-2	HCT-116	MCF-7	WI-38
**4a**	H	H	H	12.88 ± 0.9	9.94 ± 0.8	5.91 ± 0.3	66.22 ± 3.5
**4b**	H	CH_3_	H	48.25 ± 2.5	61.78 ± 3.3	46.53 ± 2.6	80.95 ± 4.2
**4c**	H	OCH_3_	H	42.22 ± 2.6	65.48 ± 3.5	54.46 ± 2.9	91.31 ± 4.6
**4d**	H	Cl	H	30.20 ± 2.3	39.69 ± 2.3	33.64 ± 2.0	89.09 ± 4.3
**4e**	H	F	H	63.39 ± 3.4	82.45 ± 4.2	67.10 ± 3.4	>100
**4f**	H	CF_3_	Cl	5.05 ± 0.3	6.21 ± 0.4	2.74 ± 0.1	41.24 ± 2.4
**4g**	CH_3_	H	H	22.93 ± 1.8	19.60 ± 1.3	16.46 ± 1.2	79.42 ± 4.0
**4h**	CH_3_	CH_3_	H	37.36 ± 2.5	56.22 ± 3.0	40.62 ± 2.3	87.80 ± 4.4
**4i**	CH_3_	OCH_3_	H	53.10 ± 2.7	76.69 ± 3.9	59.53 ± 3.1	45.45 ± 2.6
**4j**	CH_3_	Cl	H	78.13 ± 3.9	>100	84.19 ± 4.2	>100
**4k**	CH_3_	F	H	64.23 ± 3.7	92.02 ± 4.6	69.52 ± 3.5	58.45 ± 3.2
**4l**	CH_3_	CF_3_	Cl	15.36 ± 1.2	13.54 ± 1.0	9.46 ± 0.6	47.54 ± 2.7
**4m**	Cl	H	H	29.84 ± 2.0	34.39 ± 2.1	28.93 ± 1.9	74.45 ± 3.8
**4n**	Cl	CH_3_	H	32.84 ± 2.4	44.84 ± 2.5	36.72 ± 2.1	>100
**4o**	Cl	OCH_3_	H	35.03 ± 2.4	51.63 ± 2.8	43.45 ± 2.4	26.36 ± 1.8
**4p**	Cl	Cl	H	57.03 ± 3.1	71.29 ± 3.7	62.57 ± 3.2	94.11 ± 4.9
**4q**	Cl	F	H	27.38 ± 2.2	25.39 ± 1.8	21.72 ± 1.5	49.73 ± 2.9
**4r**	Cl	CF_3_	Cl	8.10 ± 0.6	7.81 ± 0.6	3.85 ± 0.2	38.77 ± 2.2
SOR				9.18 ± 0.6	5.47 ± 0.3	7.26 ± 0.3	10.65 ± 0.8

**Table 2:** Inhibitory effects of compounds **4a, 4f,** and **4r** against VEGFR-2 and BRAF (V600E).

Comp. No.	IC_50_ (µM)	
	VEGFR-2	BRAF
**4a**	0.342	0.677
**4f**	0.071	0.194
**4r**	0.794	1.359
SOR	0.069	0.171

**Table 3:** Effect of compound **4f** on the cell cycle progression in MCF-7 cells.

Comp. No	Cell Cycle Distribution (%)		
	G0-G1	S	G2-M
**4f**	64.51	22.72	12.77
Control	57.82	26.33	15.85

**Table 4:** Binding score of the compounds under investigation and reference inhibitors.

Compound	VEGFR-2 Binding Energy (kcal/mol) RMSD = 0.5	BRAF Binding Energy (kcal/mol) RMSD = 0.8
**4a**	−9.4	−8.5
**4f**	−10.4	−9.3
**4r**	−8.9	−8.2
Sorafenib	−11.6	−10.2
Dabrafenib	---	−9.7

## Data Availability

The data supporting this study’s findings are available from the corresponding author, A.H., upon reasonable request.

## Supplementary Materials

The following supporting information can be downloaded at: https://www.mdpi.com/article/10.3390/molecules29133186/s1. ^1^H-NMR spectra for final compounds (Figures S1–S17); ^13^C-NMR spectra for final compounds (Figures S18–S24); Mass charts of final compounds (Figures S25–S34) and IR spectra for final compounds (Figures S35–S40).
