# Supplementary material for: Identification of Benzothiazoles Bearing 1,3,4-Thiadiazole as Antiproliferative Hybrids Targeting VEGFR-2 and BRAF Kinase: Design, Synthesis, BIO Evaluation and In Silico Study
Source: Molecules. 2024 Jul 4;29(13):3186. doi: 10.3390/molecules29133186 (PMC11243196; doi:10.3390/molecules29133186)
Supplement: Supplementary file 1 [file molecules-29-03186-s001.zip › molecules-3072727-supplementary.pdf]

Identification of Benzothiazoles Bearing 1,3,4-Thiadiazole as Antiproliferative Hybrids Targeting VEGFR-2 and BRAF Kinase: Design, Synthesis, BIO Evaluation and In Silico Study

Wafaa A. Ewes [1,] Samar S. Tawfik [1], Aya M. Almatary [2], Mashooq Ahmad Bhat [3], Hamed W. El-Shafey [1], Ahmed A. B. Mohamed [4], Abdullah Haikal [5], Mohammed A. El-Magd [6], Abdullah A. Elgazar [7], Marwa Balaha [8] and Abdelrahman Hamdi [1,*]

1 Department of Pharmaceutical Organic Chemistry, Faculty of Pharmacy, Mansoura University, Mansoura 35516, Egypt; wafaa_ewes@mans.edu.eg (W.A.E.); drsamarelmasry@mans.edu.eg (S.S.T.); hamedelshafey@mans.edu.eg (H.W.E.-S.)
2 Department of Pharmaceutical Organic Chemistry, Faculty of Pharmacy, Horus University-Egypt, New Damietta 34518, Egypt; aelmatary@horus.edu.eg
3 Department of Pharmaceutical Chemistry, College of Pharmacy, King Saud University, P.O Box 2457, Riyadh 11451, Saudi Arabia; mabhat@ksu.edu.sa
4 Department of Medicinal Chemistry, Faculty of Pharmacy, Mansoura University, Mansoura 35516, Egypt; ahmed_bakr@mans.edu.eg
5 Department of Pharmacognosy, Faculty of Pharmacy, Mansoura University, Mansoura 35516, Egypt; abdullahhaikal@mans.edu.eg
6 Department of Anatomy, Faculty of Veterinary Medicine, Kafrelsheikh University, Kafrelsheikh 33516, Egypt; mohamed.abouelmagd@vet.kfs.edu.eg
7 Department of Pharmacognosy, Faculty of Pharmacy, Kafrelsheikh University, Kafrelsheikh 33516, Egypt; abdulah.elgazar@phr.mans.edu.eg
8 Department of Medical, Oral and Biotechnological Sciences, “G. d’Annunzio” University of Chieti-Pescara, Via dei Vestini, 31, 66100 Chieti, Italy; marwa.balaha@unich.it
* Correspondence: abdelrahmanhamdi2012@yahoo.com or abdelrahmanhamdi2012@mans.edu.eg

Content

Fig S1. ^{1}H-NMR of **4a** …………………………………………………………………….…… 3

Fig S2. ^{1}H-NMR of **4b** ………………………………………………………………......… 4

Fig S3. ^{1}H-NMR of **4c** ……………………………………………………………..………5

Fig S4. ^{1}H-NMR of **4d** ………………………………………………………………….… 6

Fig S5. ^{1}H-NMR of **4e** ……………………………………………………………..……… 7

Fig S6. ^{1}H-NMR of **4f** ……………………………………………………………..……… 8

Fig S7. ^{1}H-NMR of **4h** …………………………………………………………….……… 9

Fig S8. ^{1}H-NMR of **4i** ……………….………………………………………….……….. 10

Fig S9. ^{1}H-NMR of **4g** ……………………………………………………….……….…. 11

Fig S10. ^{1}H-NMR of **4j** …………………………………………………….………….…. 12

Fig S11. ^{1}H-NMR of **4k** ……………………………………………...……..……… 13

Fig S12. ^{1}H-NMR of **4l** ……………………………………………...….………….... 14

Fig S13. ^{1}H-NMR of **4n** ……………………………………………...…………..…. 15

Fig S14. ^{1}H-NMR of **4m** …………………………………………….....……….…. 16

Fig S15. ^{1}H-NMR of **4o** ……………………………………………...…………….... 17

Fig S16. ^{1}H-NMR of **4p** ……………………………………………...……….……….. 18

Fig S17. ^{1}H-NMR of **4q** ……………………………………………...……….……..…. 19

Fig S18. ^{13}C-NMR of **4c** ……………………………………………...…………..…. 21

Fig S19. ^{13}C-NMR of **4e** ……………………………………………..…………..…. 22

Fig S20. ^{13}C-NMR of **4d** …………………………………………..………………..…. 23

Fig S21. ^{13}C-NMR of **4i** ………………………………………………………….....…. 24

Fig S22. ^{13}C-NMR of **4j** ……………………………………………………….…......…. 25

Fig S23. ^{13}C-NMR of **4k** ………………………………………………………..…..…. 26

Fig S24. ^{13}C-NMR of **4q** ………………………………………………………..…..…. 27

Fig S25. Mass spectra of **4a** ………………………………………....……….…. 29

Fig S26. Mass spectra of **4b** ……………………………………...……….…..…. 30

Fig S27. Mass spectra of **4c** ……………………………………...…..……..…..…. 31

Fig S28. Mass spectra of **4d** ……………………………………...……….……..…. 32

Fig S29. Mass spectra of **4e** ……………………………….…………...……..…. 33

Fig S30. Mass spectra of **4j** …………………………………………………,……..… 34

Fig S31. Mass spectra of **4o** …………………………………………………,……..… 35

Fig S32. Mass spectra of **4q** ……………………………………………,………..… 36

Fig S33. Mass spectra of **4k** ………………………………………,…………..… 37

Fig S34. Mass spectra of **4m** ……………………………………………………..… 38

Fig S35. IR spectra of **4g** ………………………………..…………………………..… 40

Fig S36. IR spectra of **4j** ………………………………..…………………...……..… 41

Fig S37. IR spectra of **4l** ………………………..………...…………………...……..… 42

Fig S38. IR spectra of **4r** ………………………..………...………………..………..… 43

Fig S39. IR spectra of **4a** ………………………..………..………………..………..… 44

Fig S40. IR spectra of **4f** ………………………..………...………………..………..… 45

^{1}H-NMR

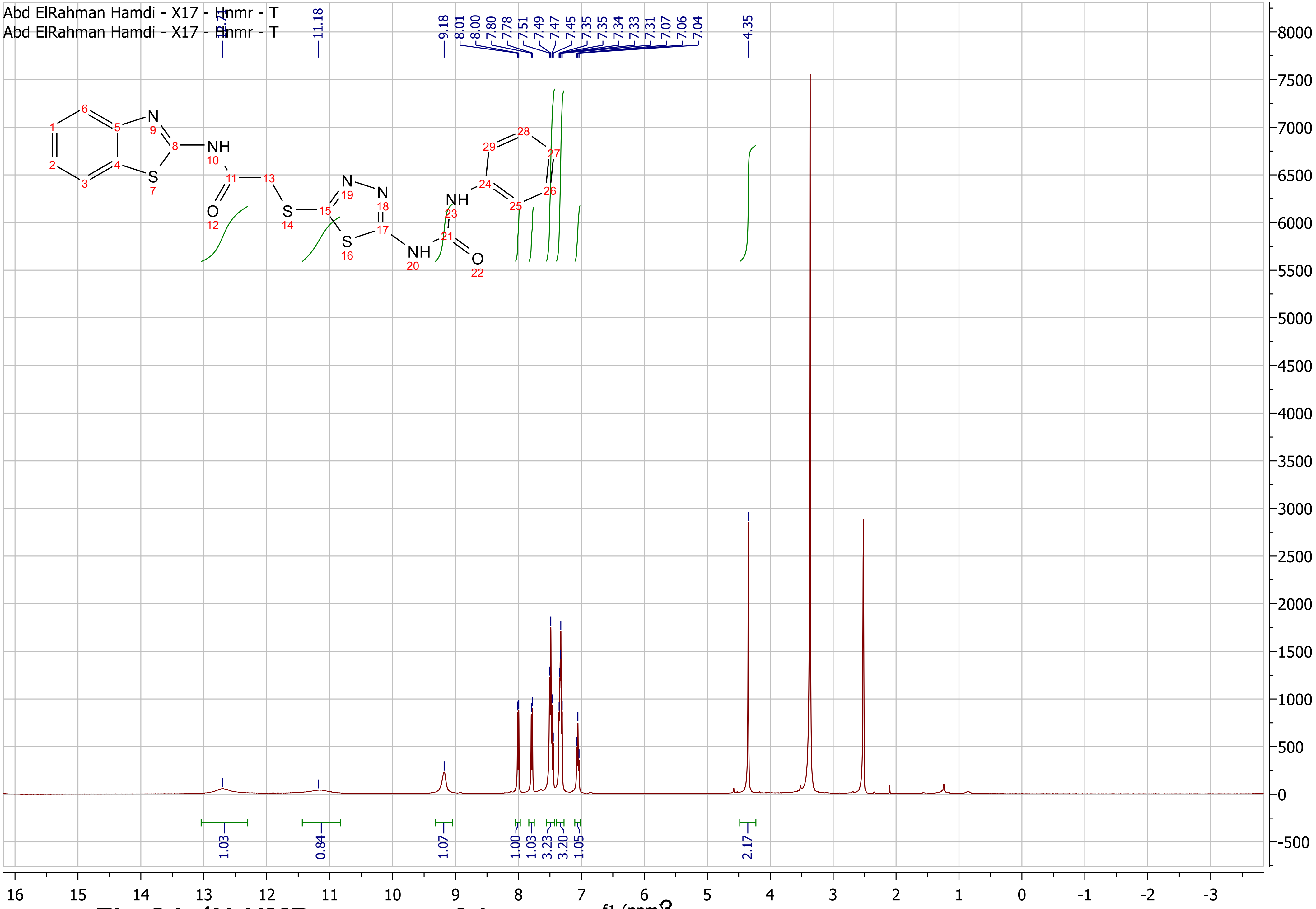

3

Fig S1. ^{1}H-NMR spectra of 4a

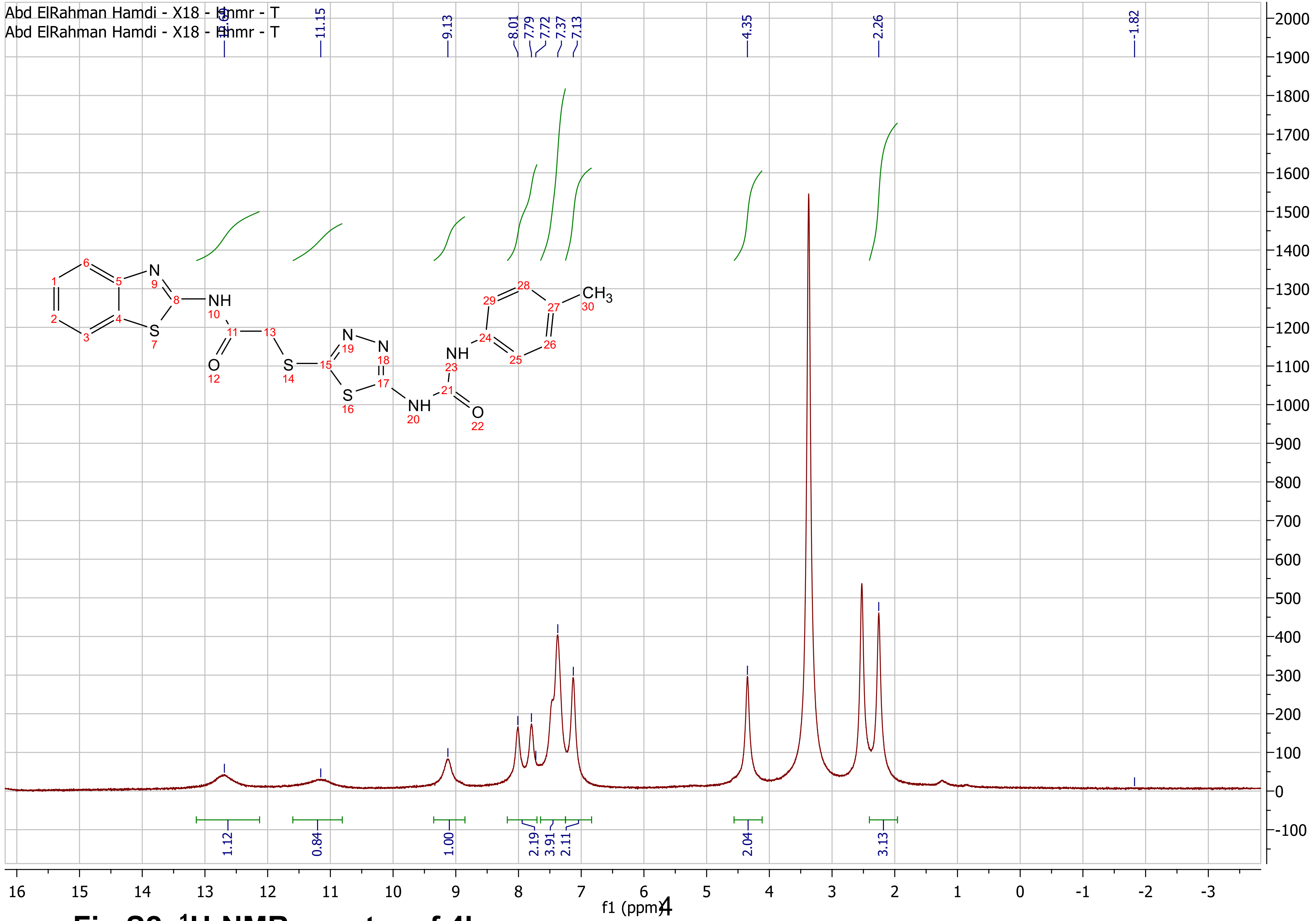

Fig S2. ^{1}H-NMR spectra of 4b

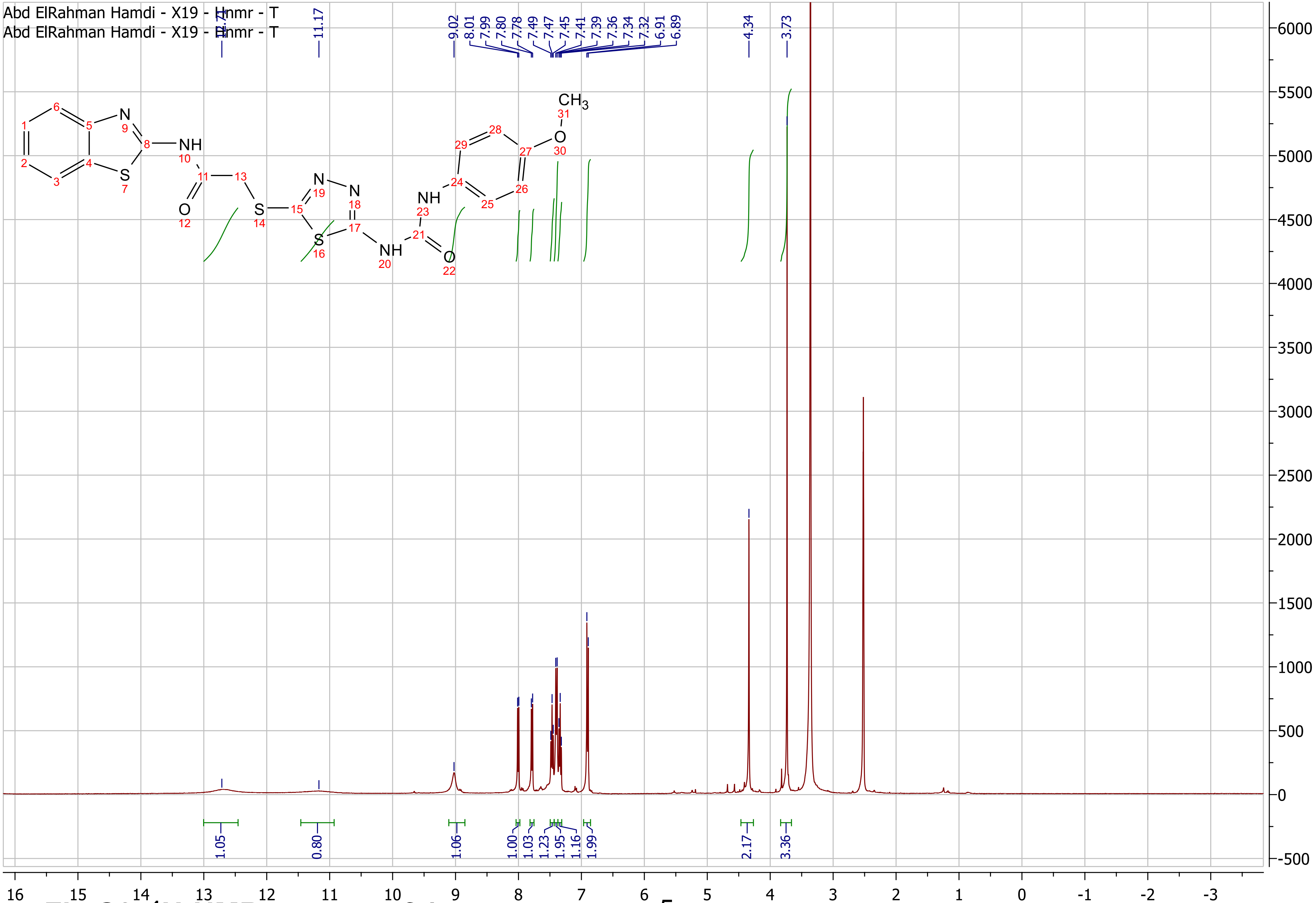

Fig S3. ¹H-NMR spectra of 4c

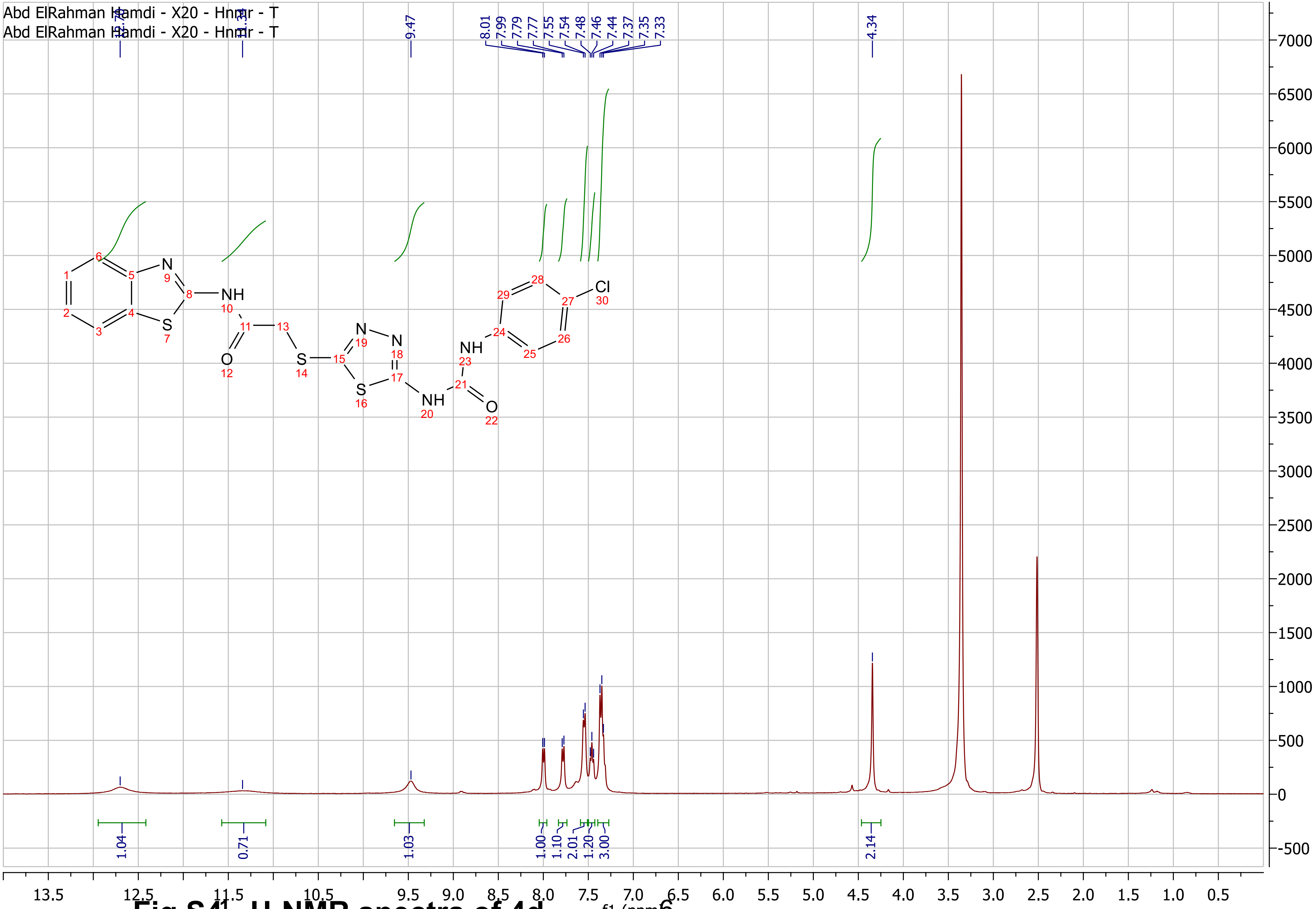

Fig S4: ^{1}H-NMR spectra of 4d

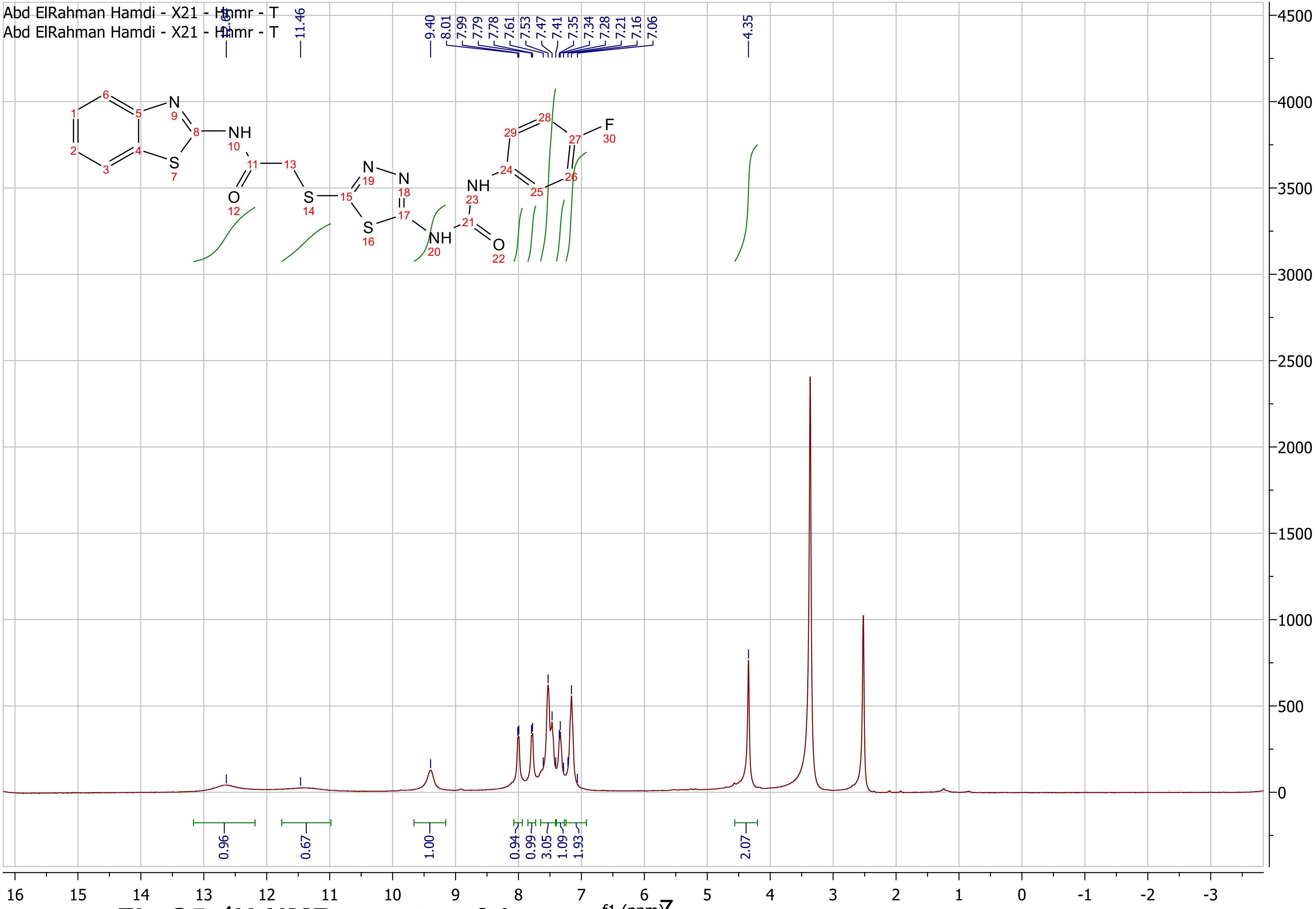

Fig S5. ^{1}H-NMR spectra of 4e

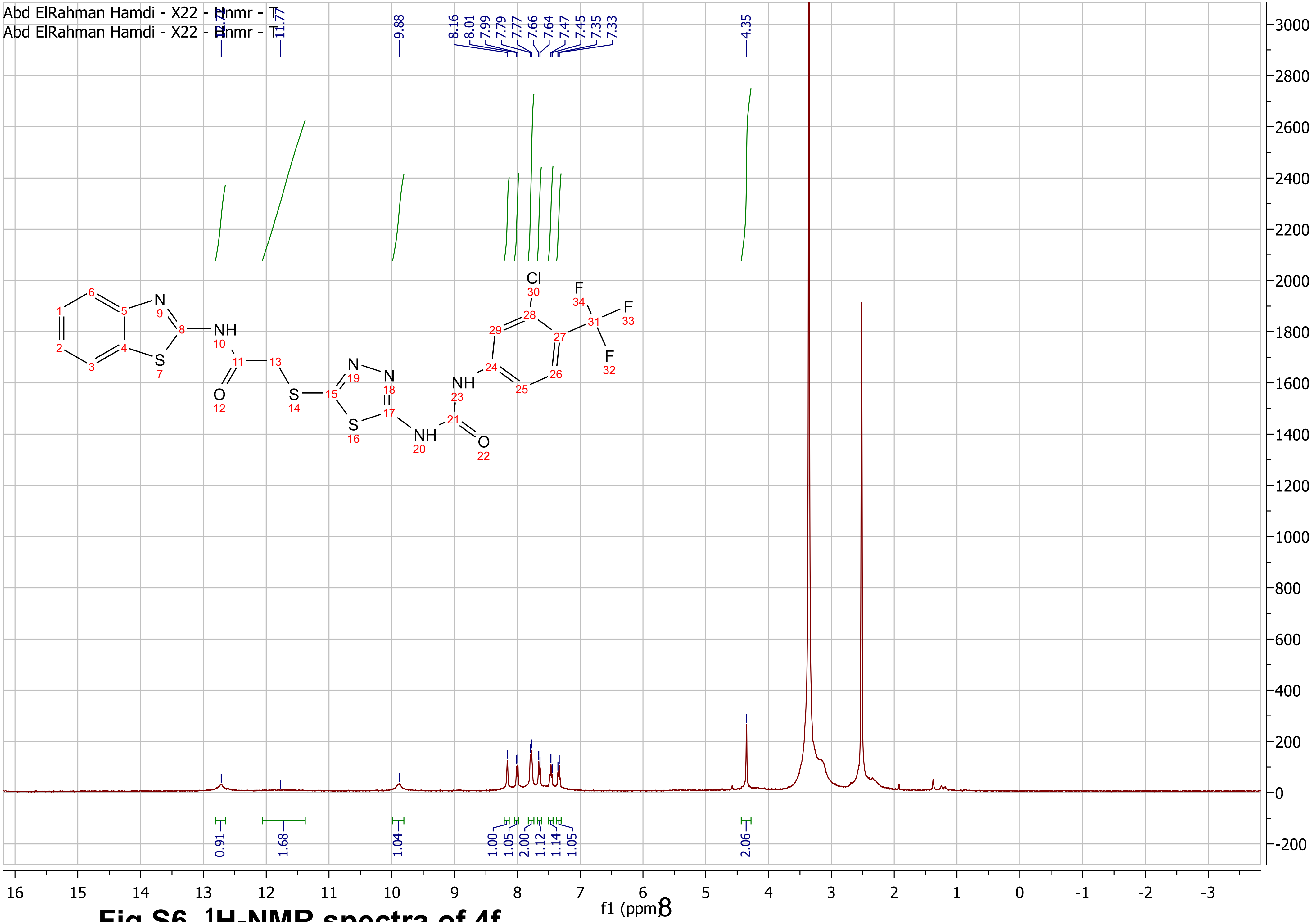

Fig S6. ¹H-NMR spectra of 4f

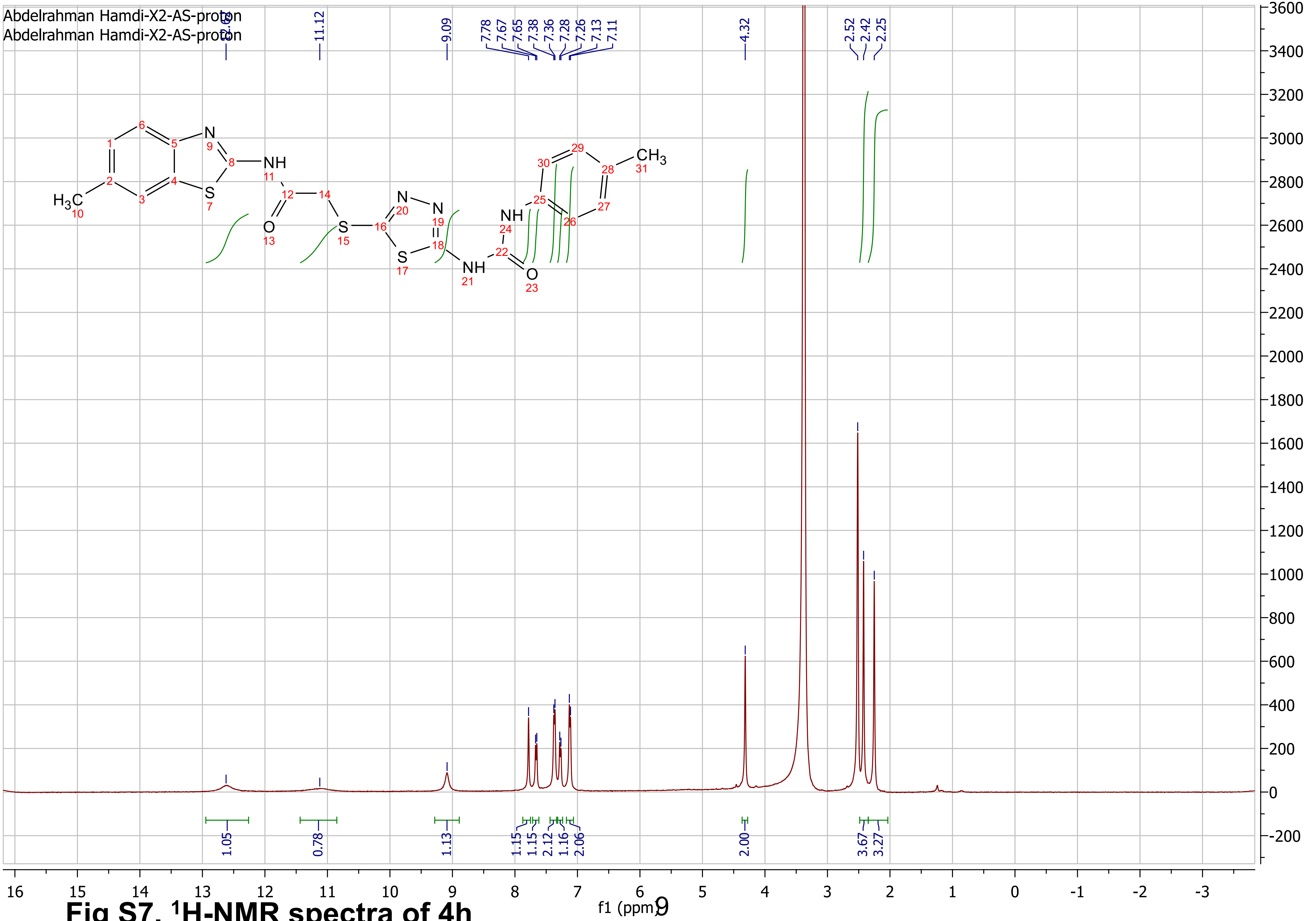

Fig S7. ^{1}H-NMR spectra of 4h

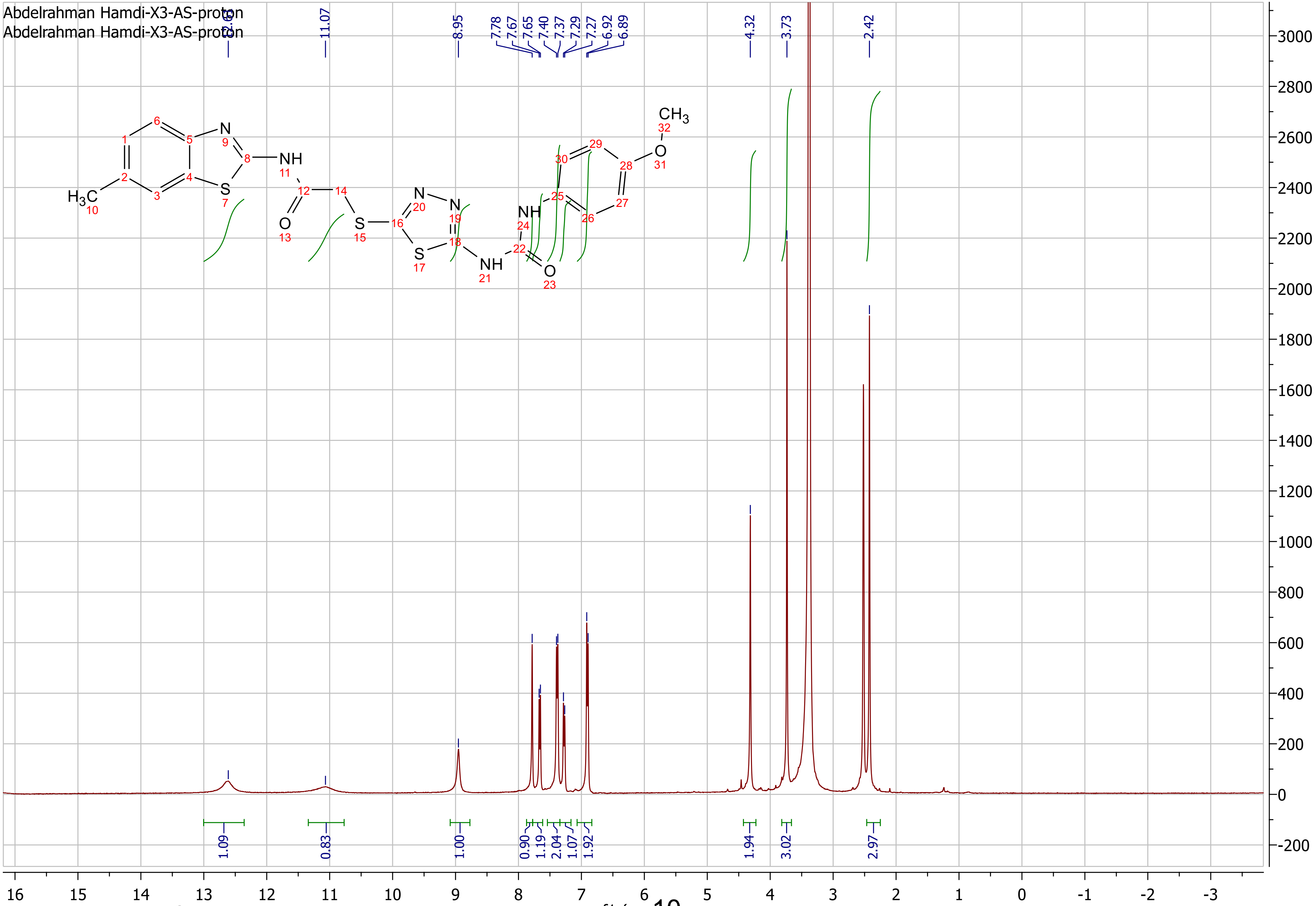

Fig S8. ^{1}H-NMR spectra of 4i

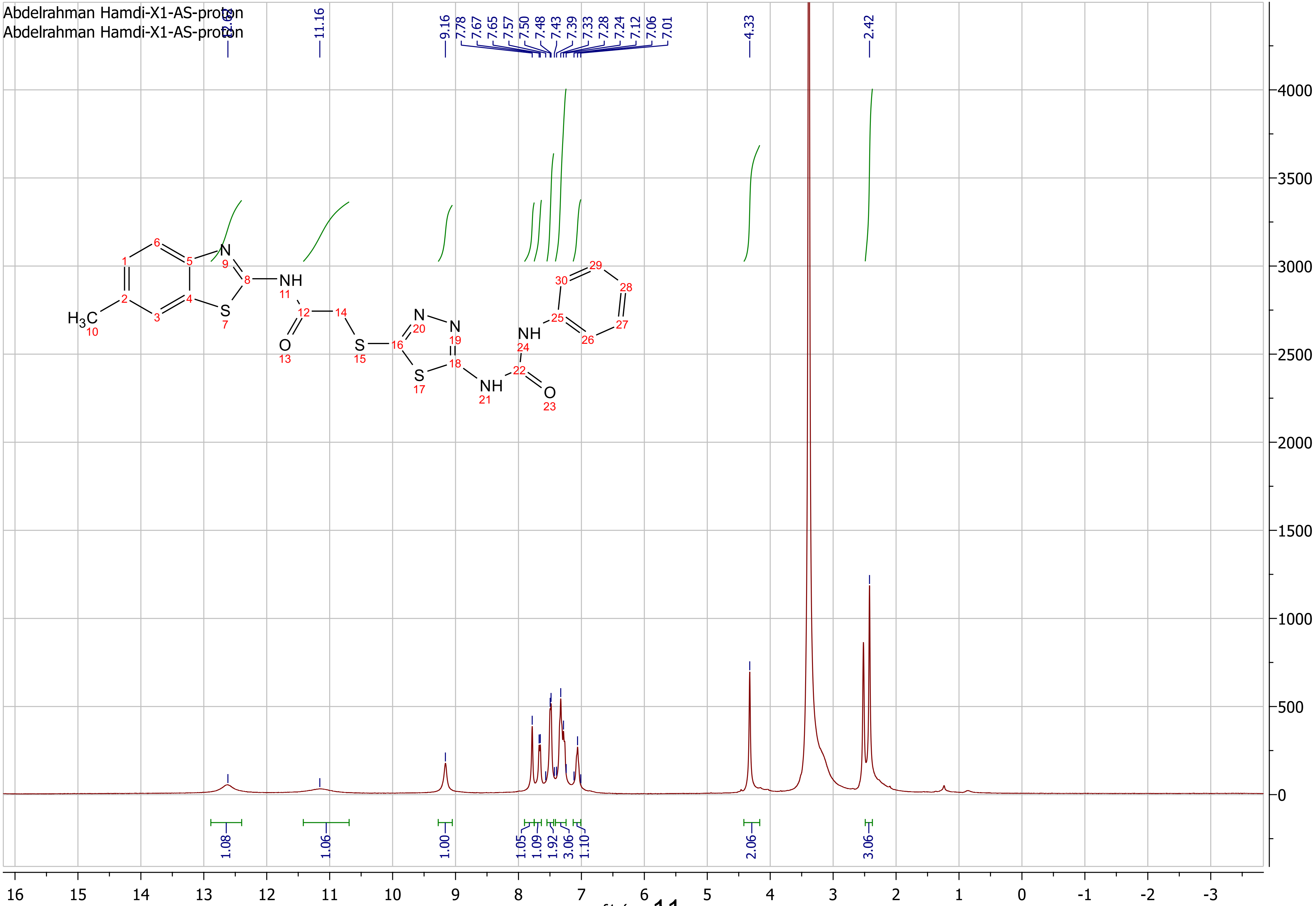

Fig S9. ^{1}H-NMR spectra of 4g

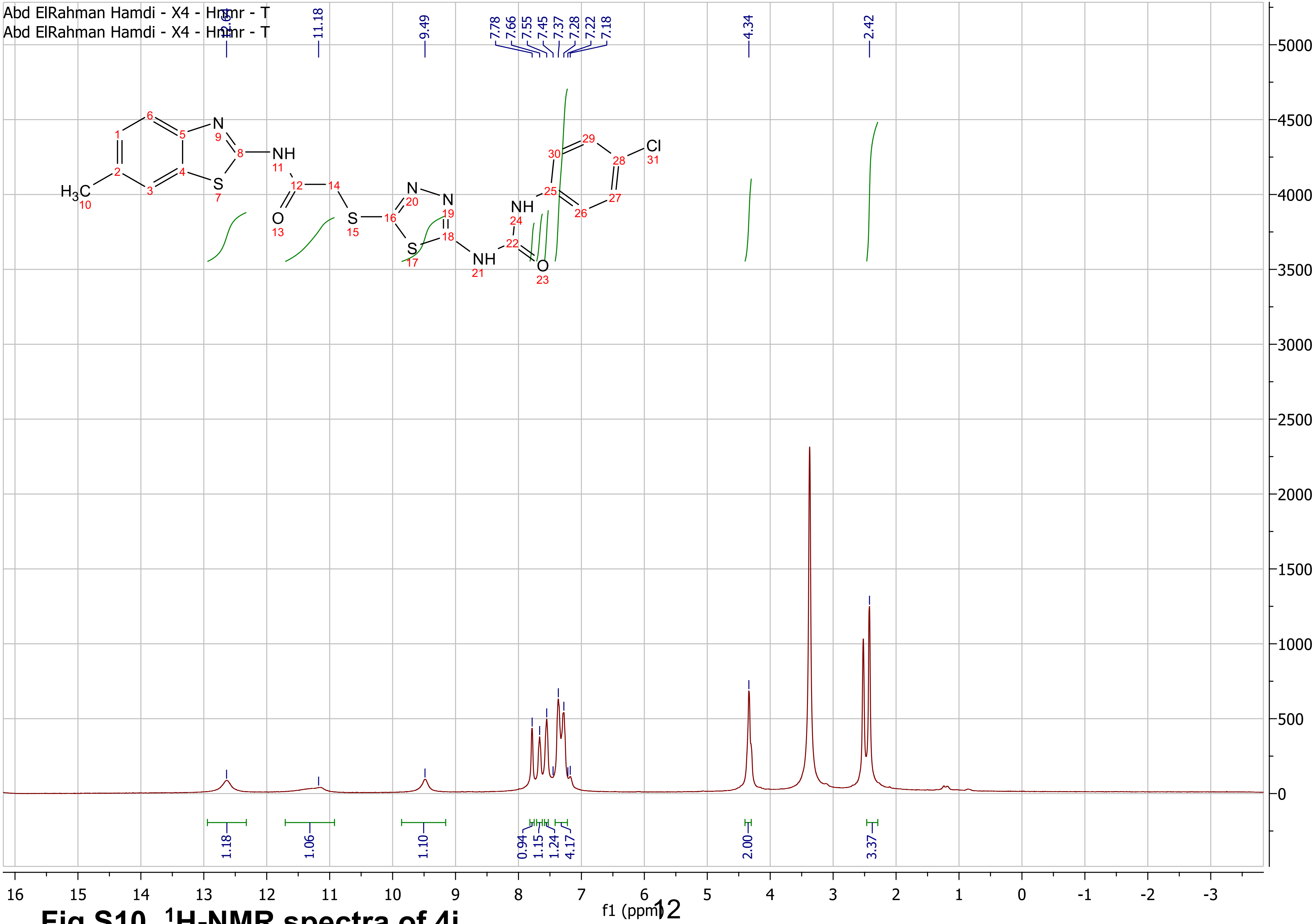

Fig S10. ^{1}H-NMR spectra of 4j

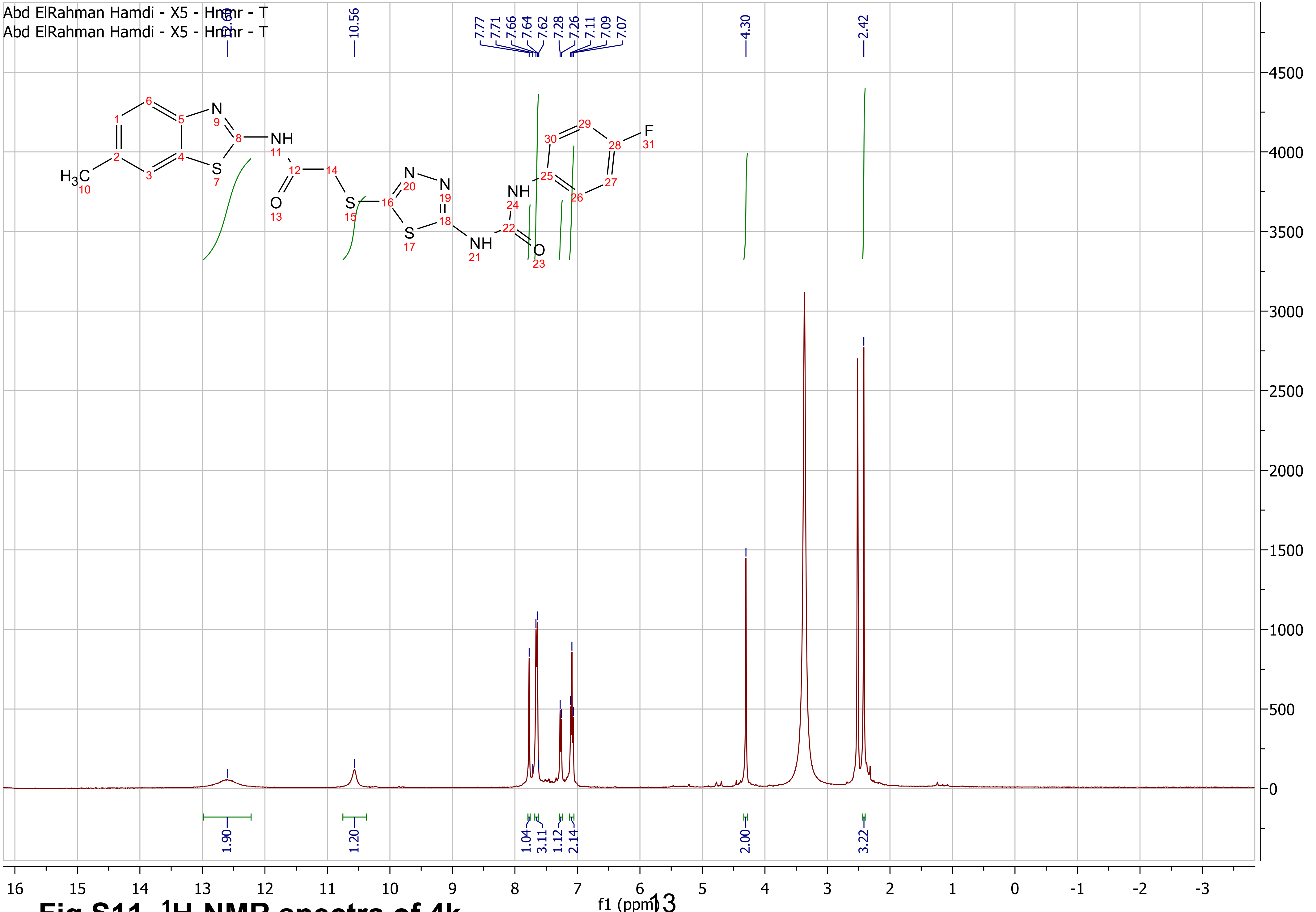

13

Fig S11. ^{1}H-NMR spectra of 4k

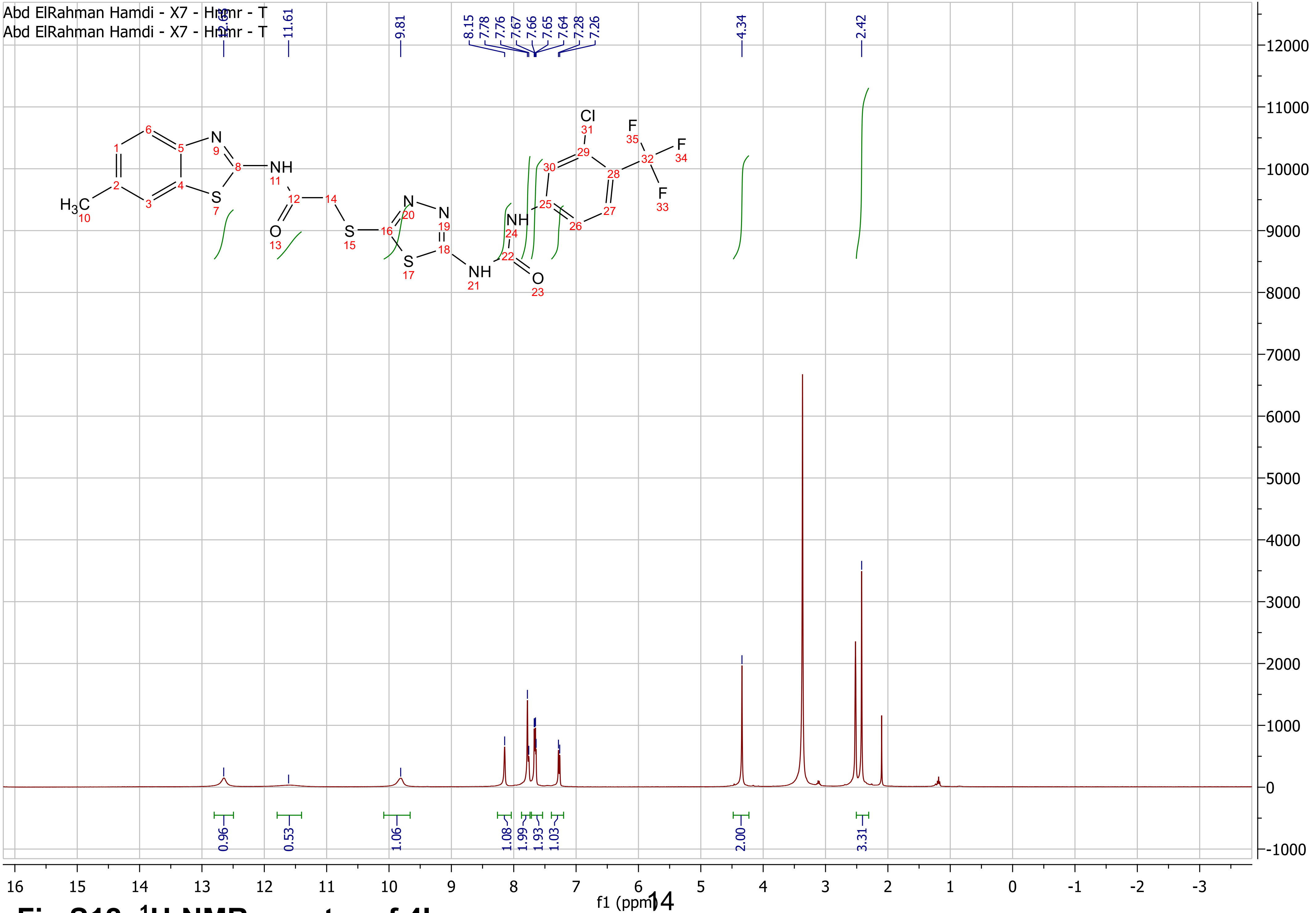

Fig S12. ^{1}H-NMR spectra of 4l

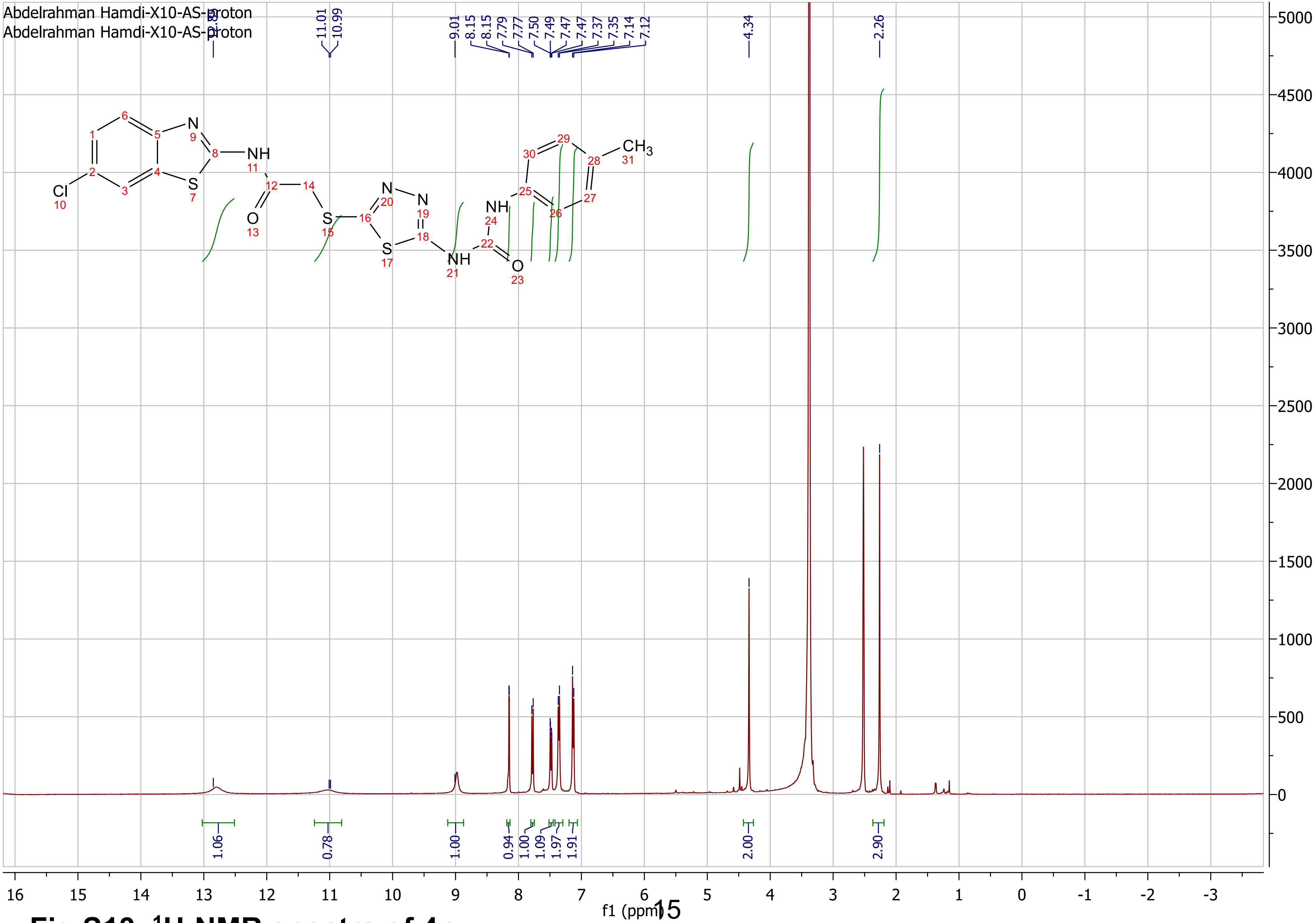

Fig S13. ^{1}H-NMR spectra of 4n

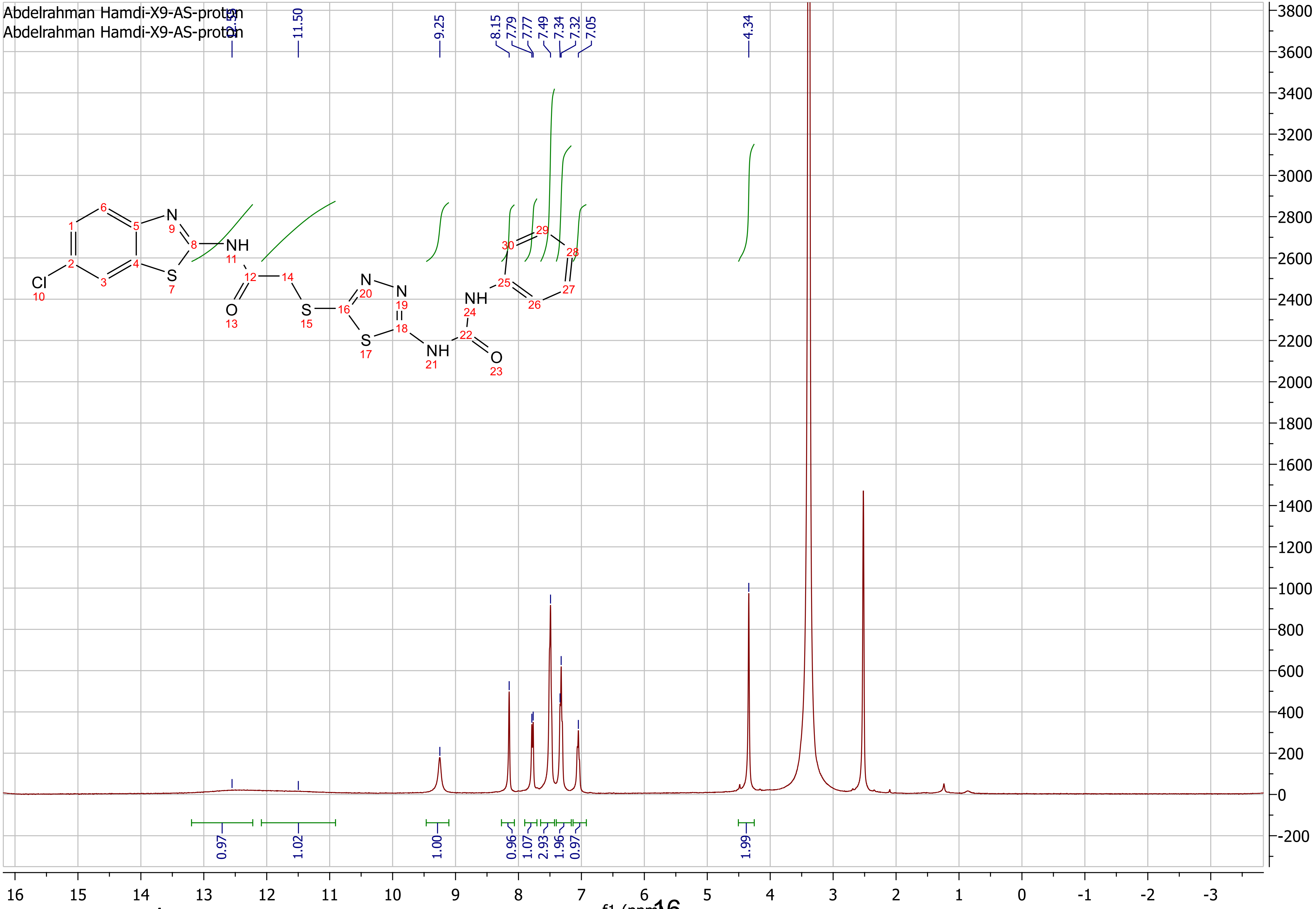

Fig S14. ^{1}H-NMR spectra of 4m

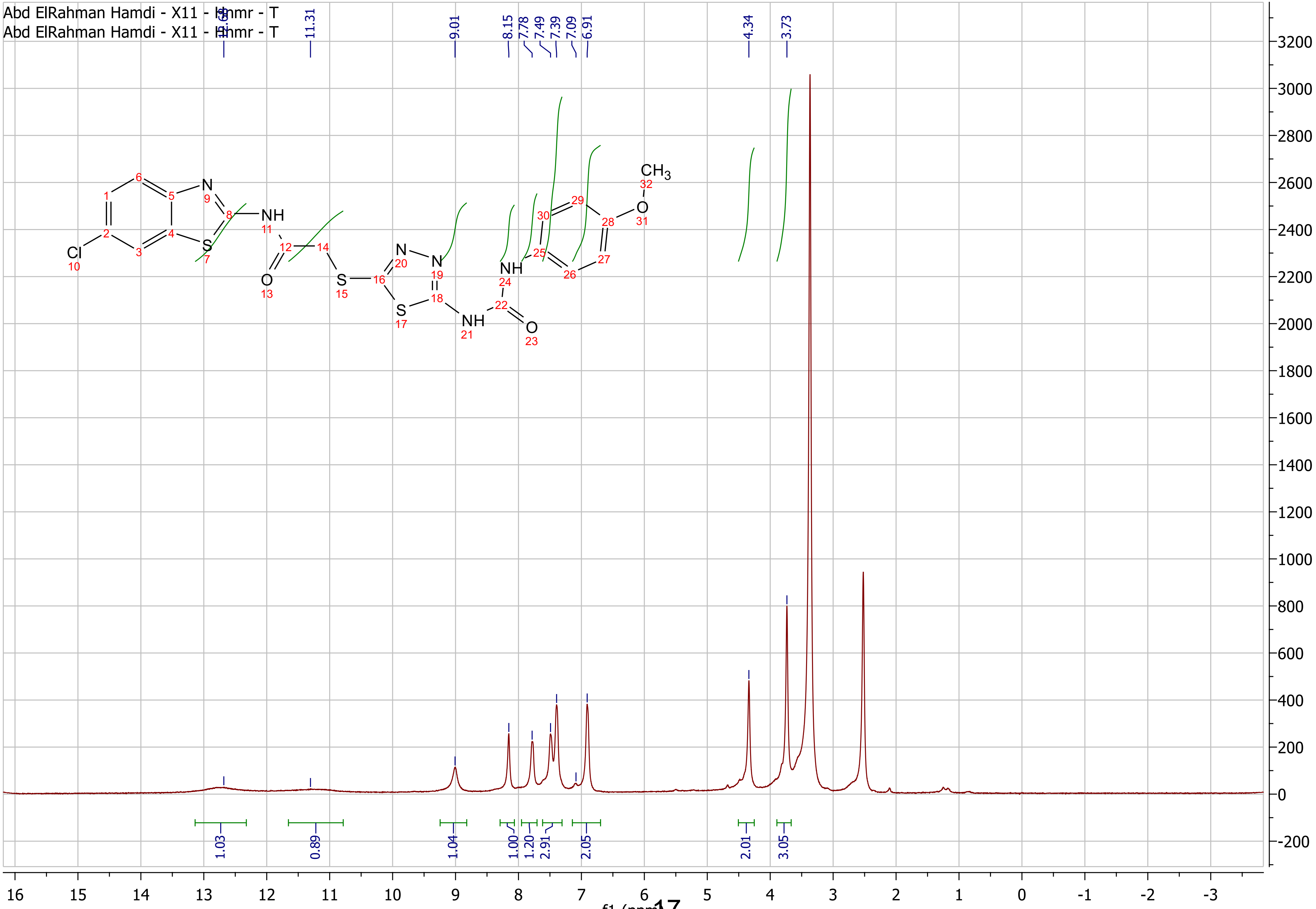

Fig S15. ^{1}H-NMR spectra of 4o

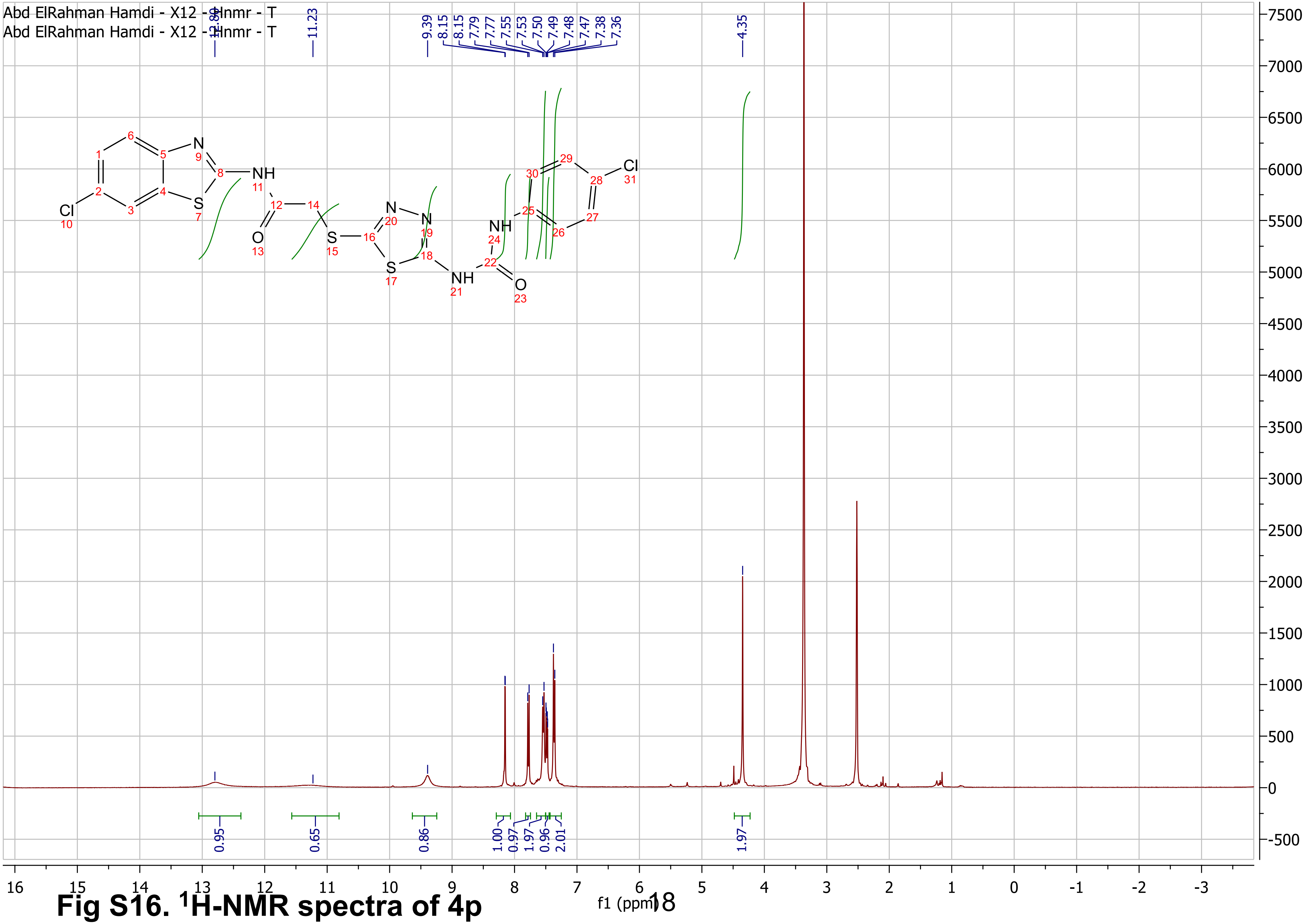

Fig S16. ^{1}H-NMR spectra of 4p

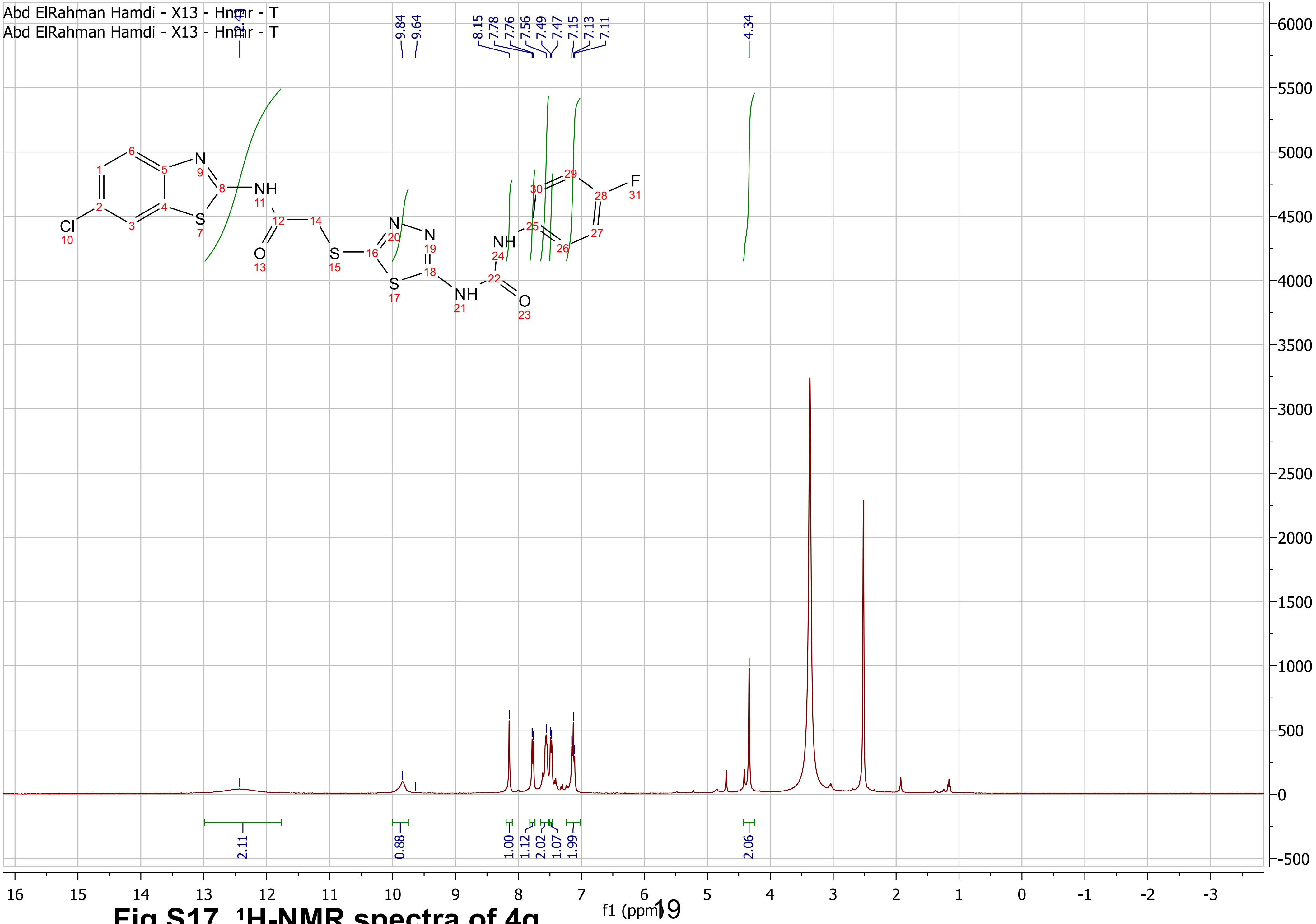

19

Fig S17. ¹H-NMR spectra of 4q

^{13}C-NMR

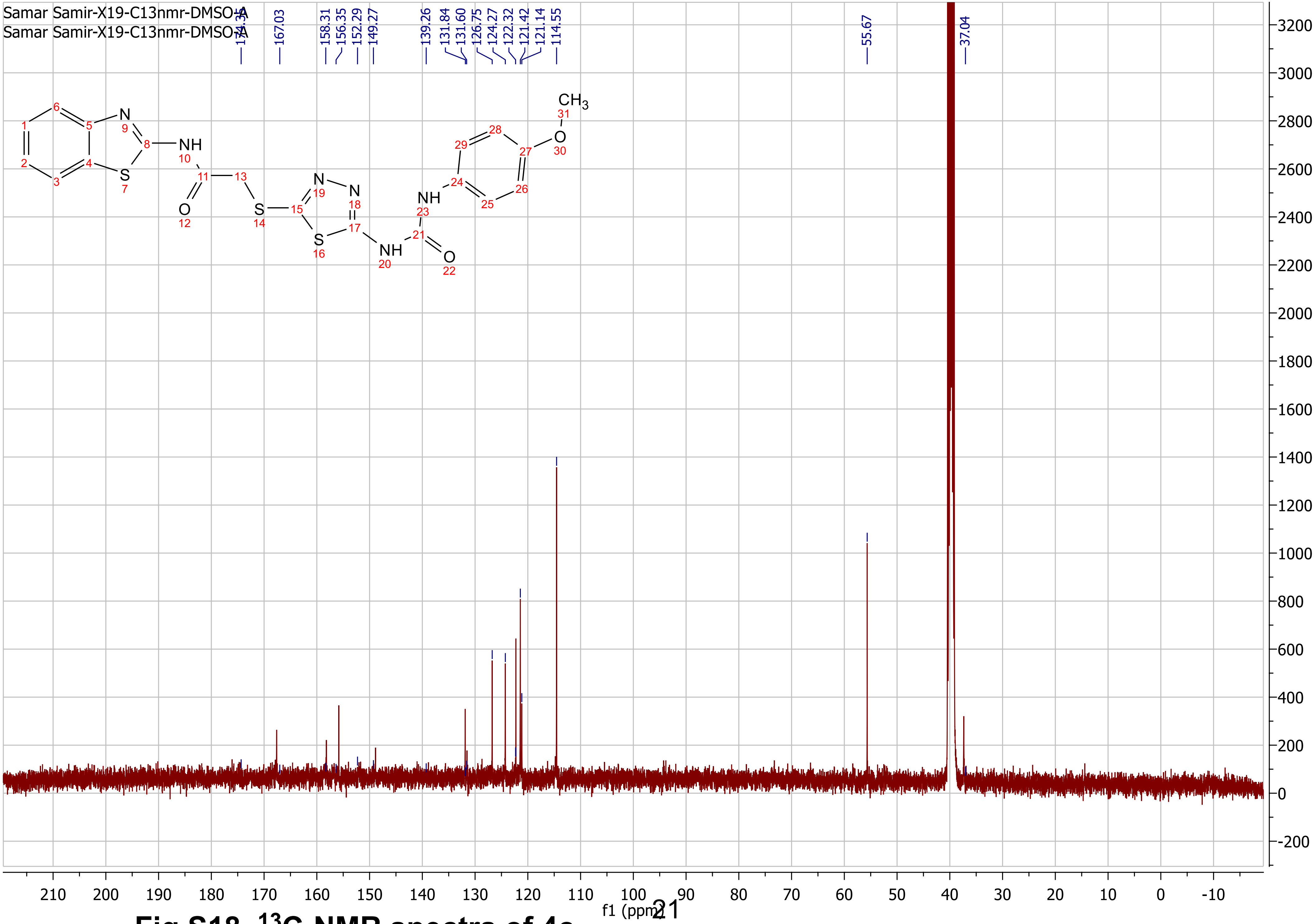

Fig S18. ^{13}C-NMR spectra of 4c

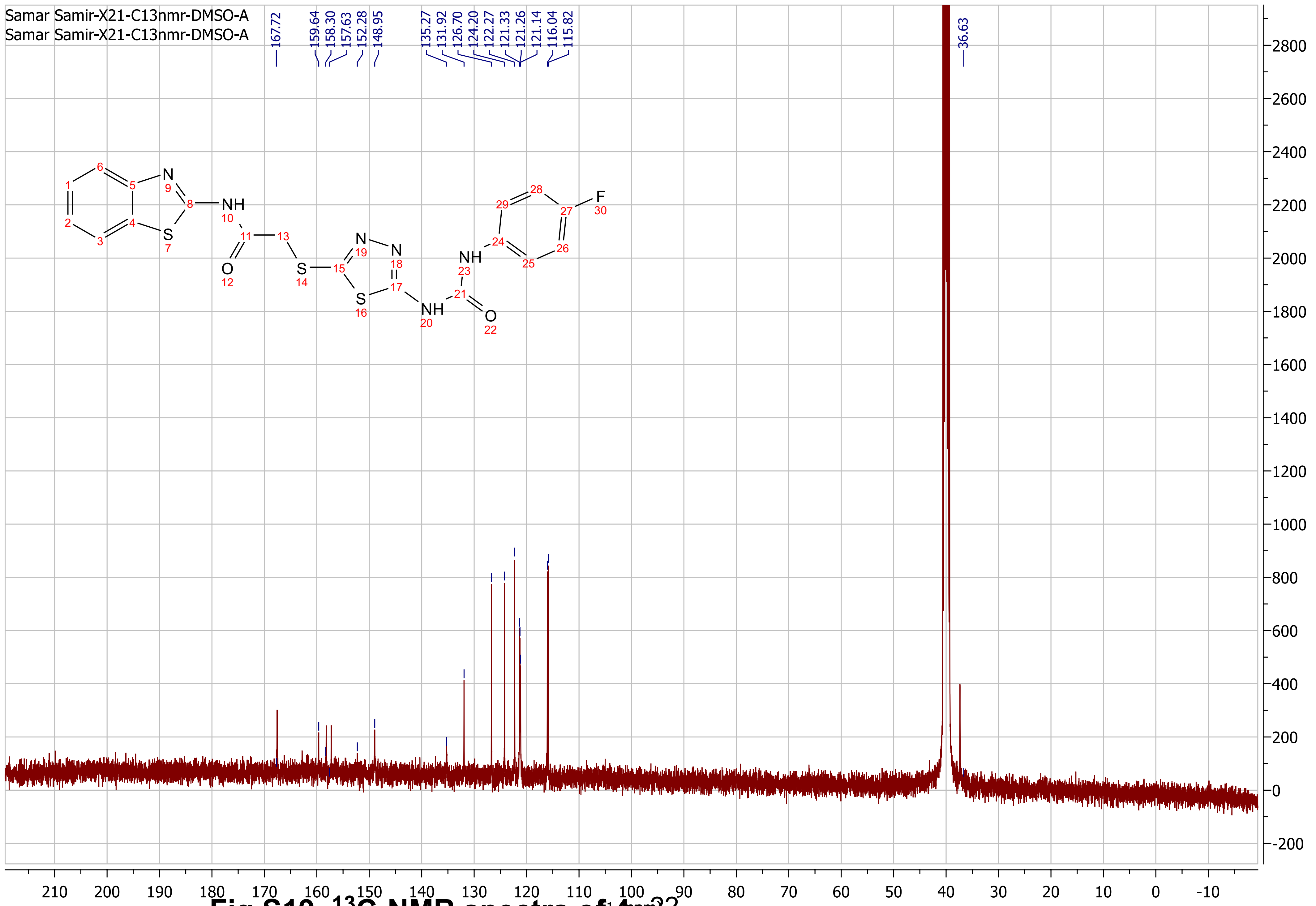

Fig S19. ^{13}C-NMR spectra of 4e

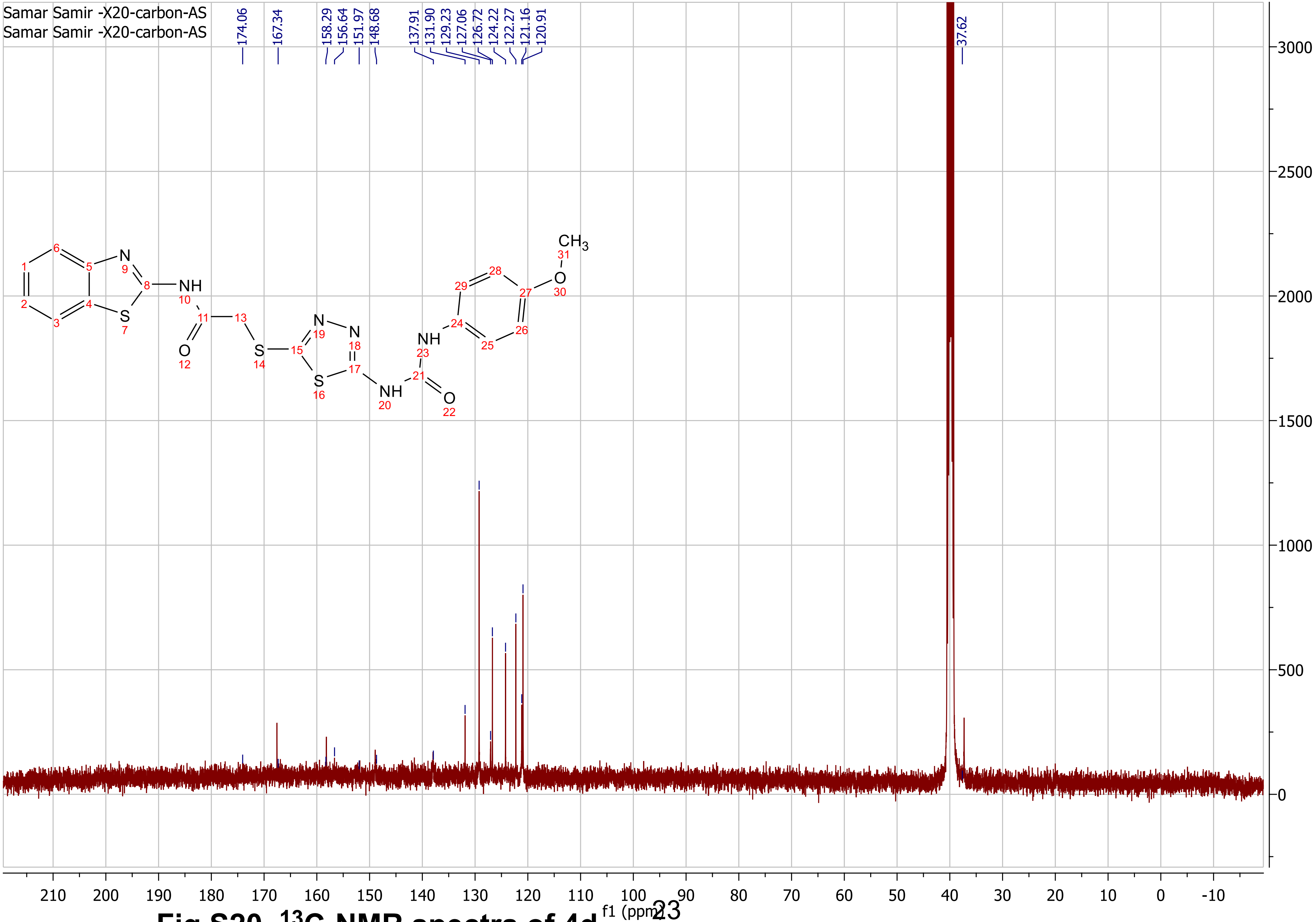

Fig S20. ^{13}C-NMR spectra of 4d

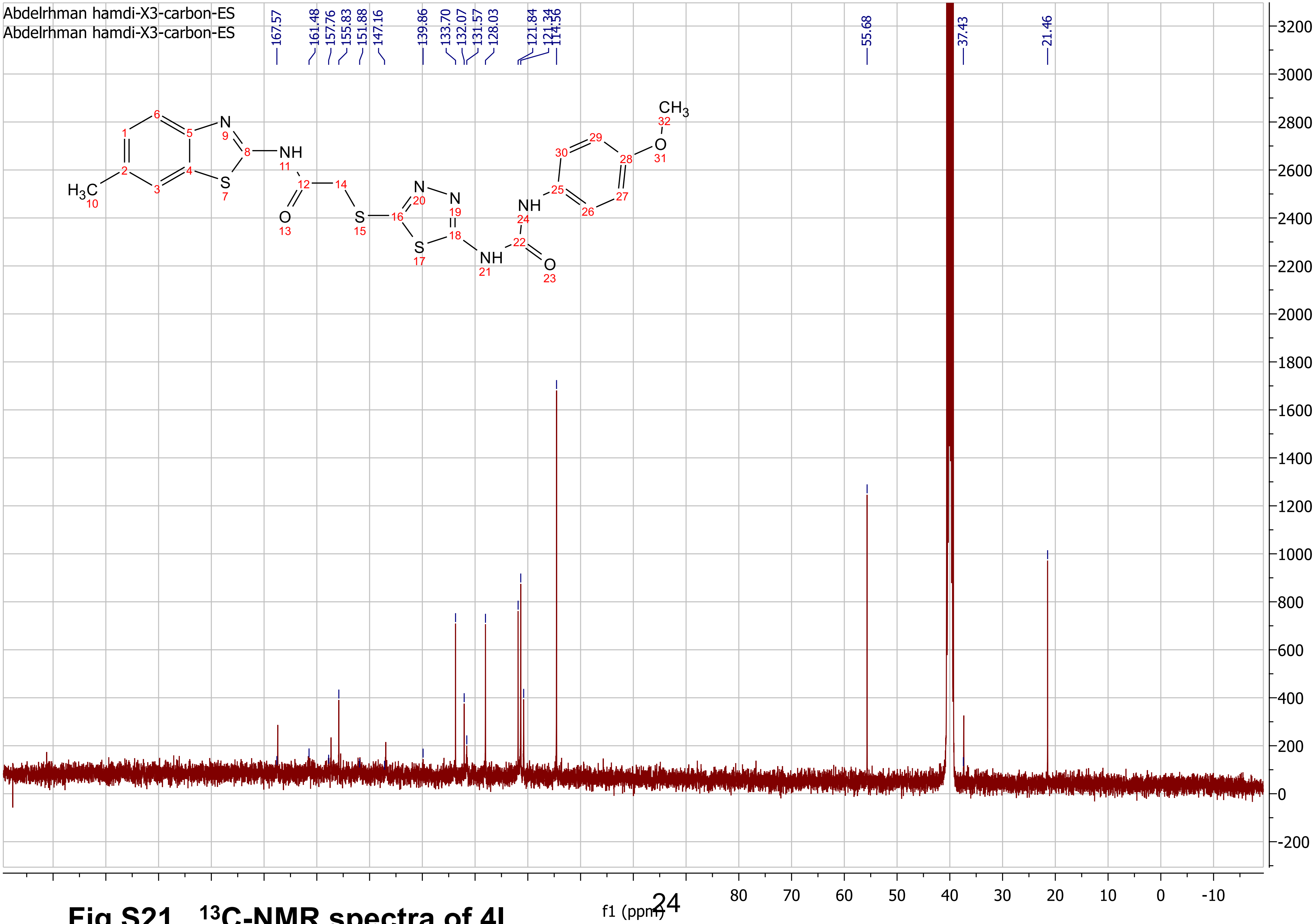

Fig S21. ^{13}**C-NMR spectra of 4l**

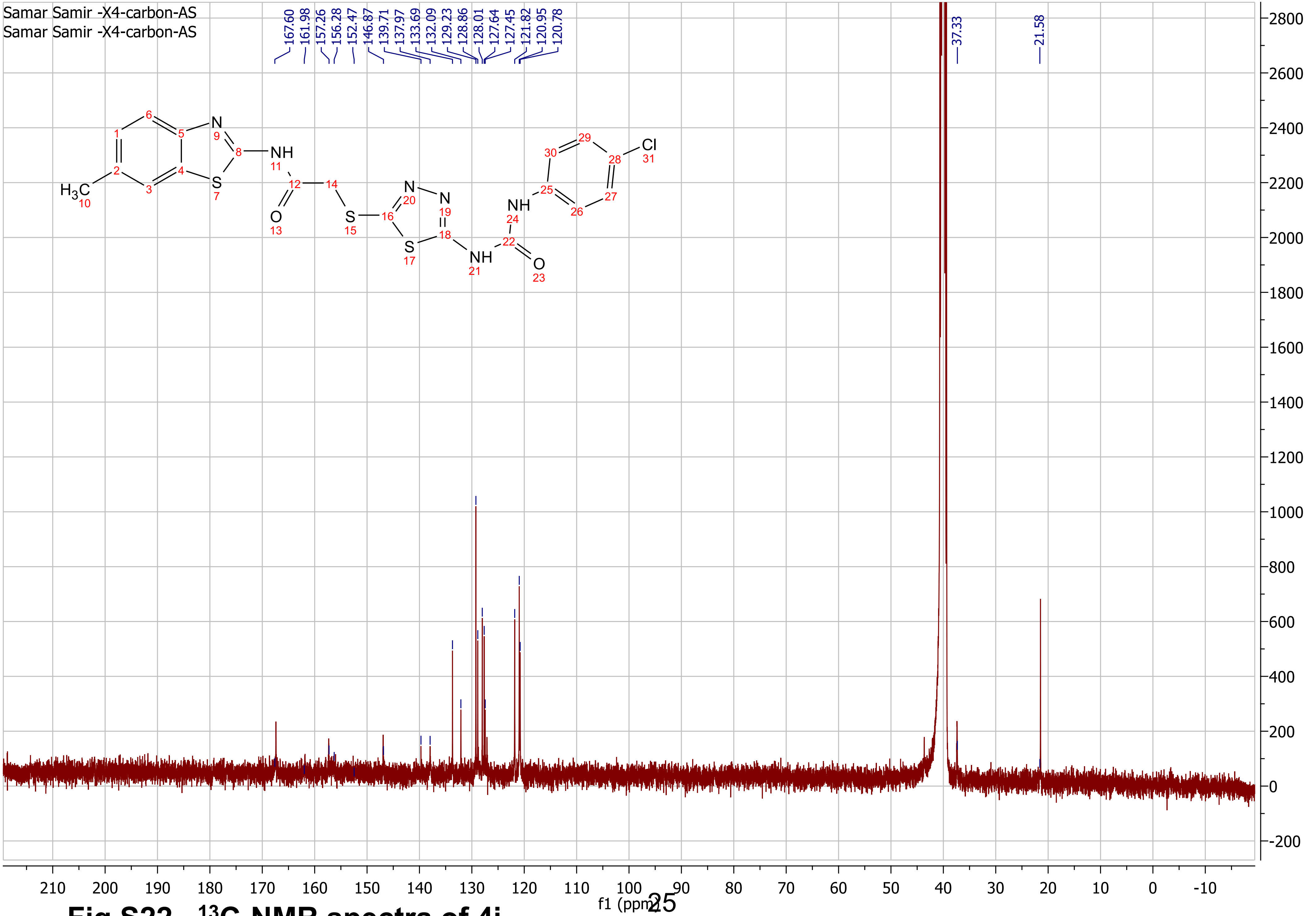

Fig S22. ^{13}C-NMR spectra of 4j

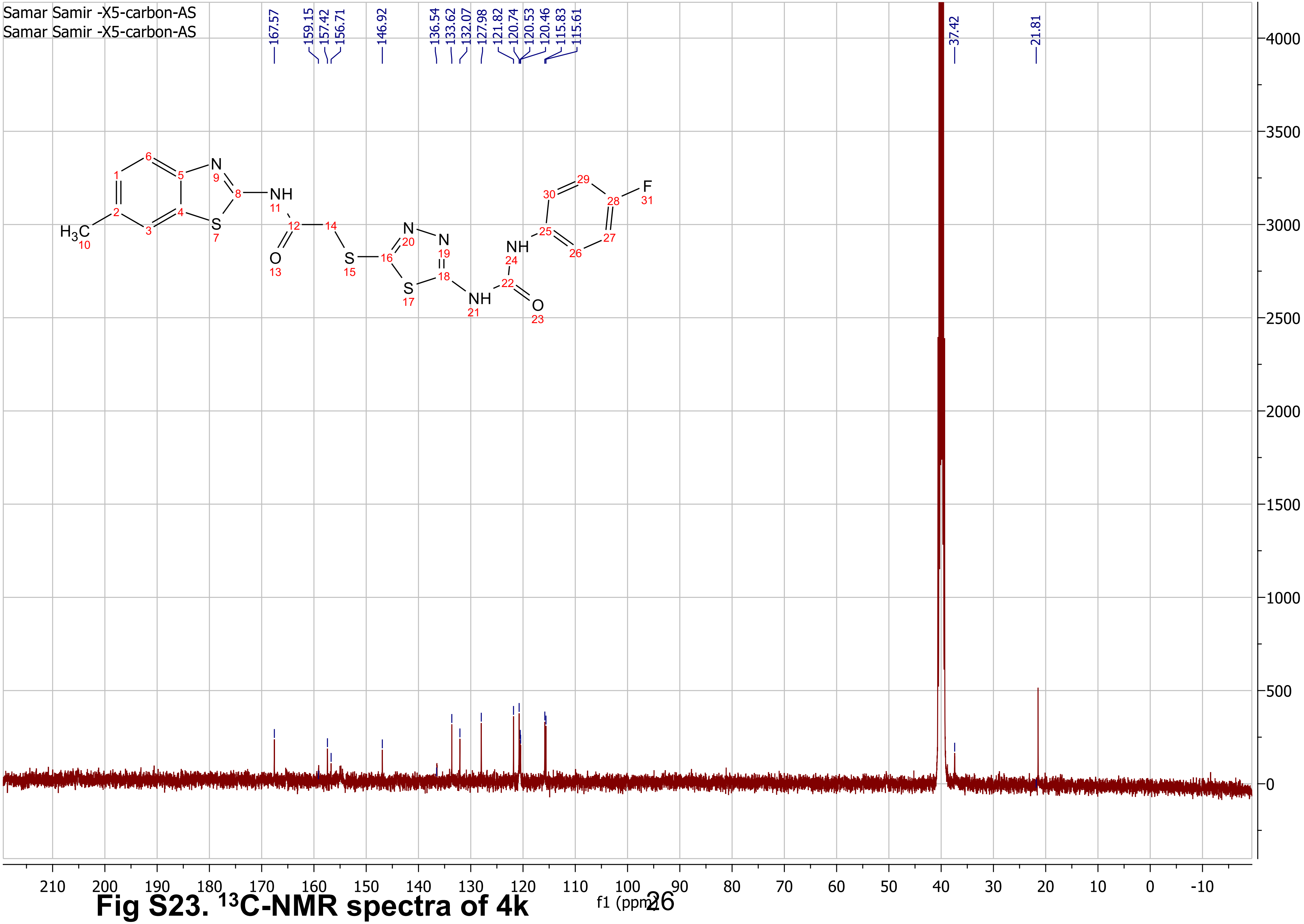

Fig S23. ^{13}C-NMR spectra of 4k

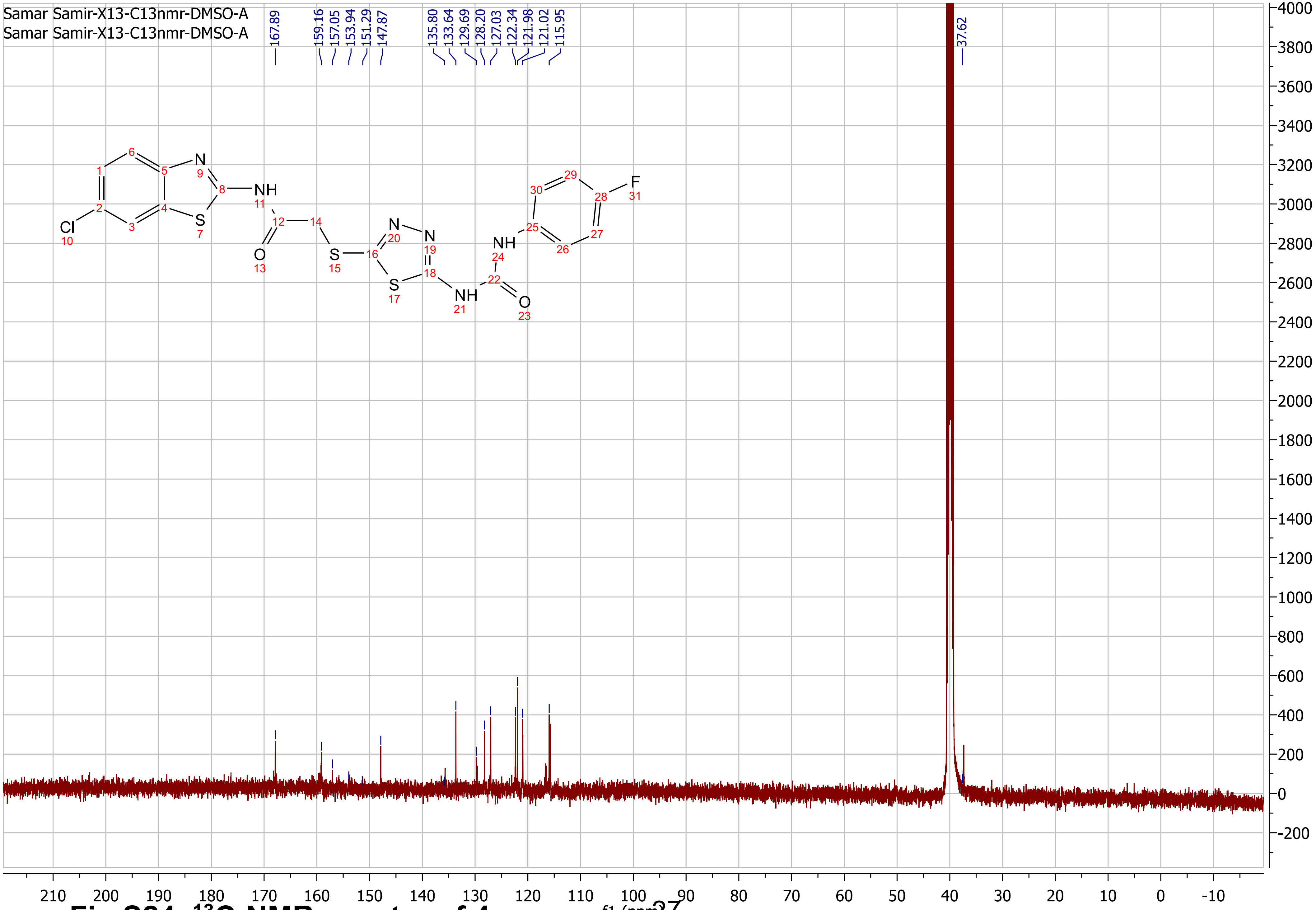

Fig S24. ^{13}C-NMR spectra of 4q

Mass spectra

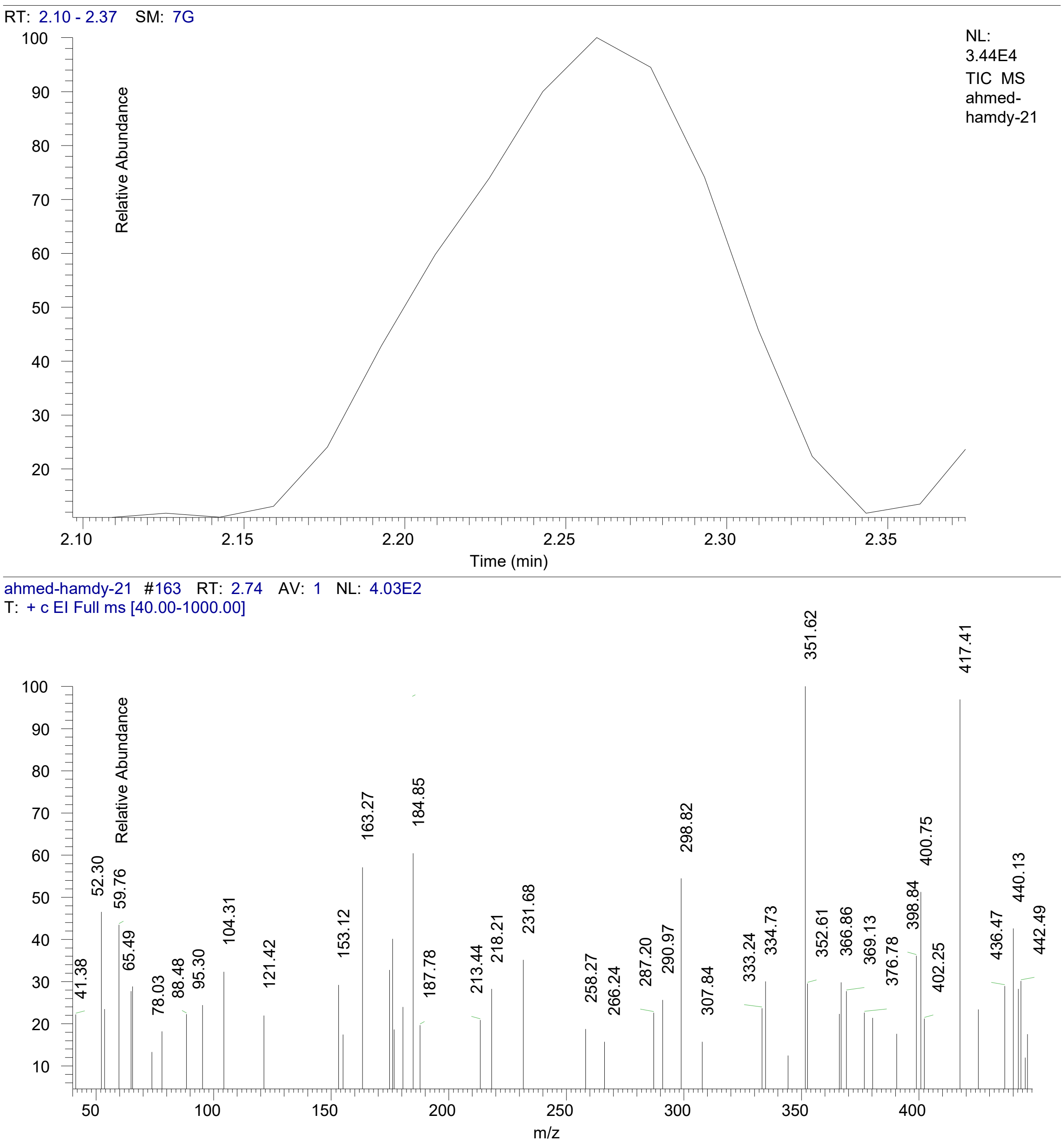

Fig S25. Mass spectra of 4a

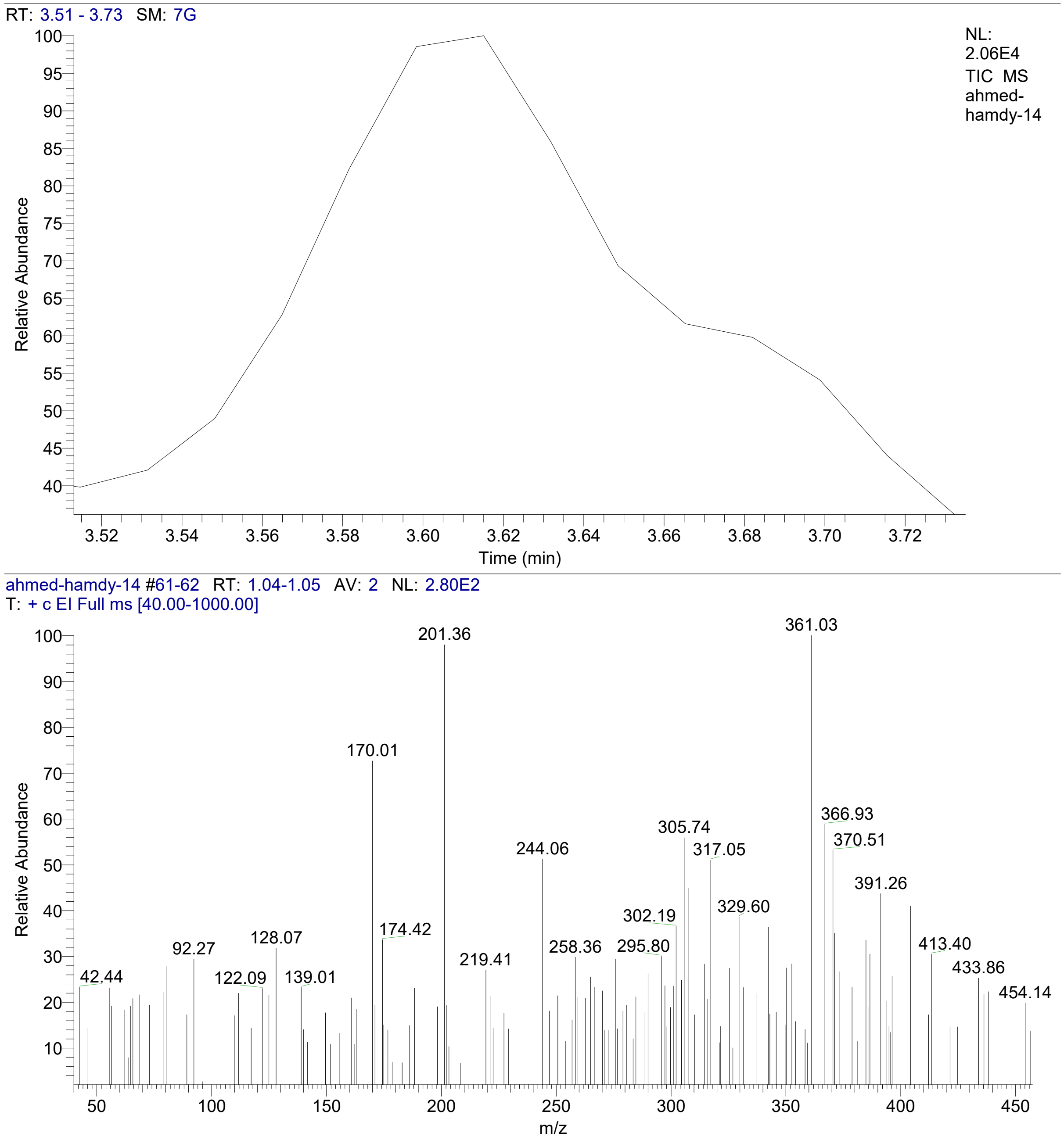

Fig S26. Mass spectra of 4b

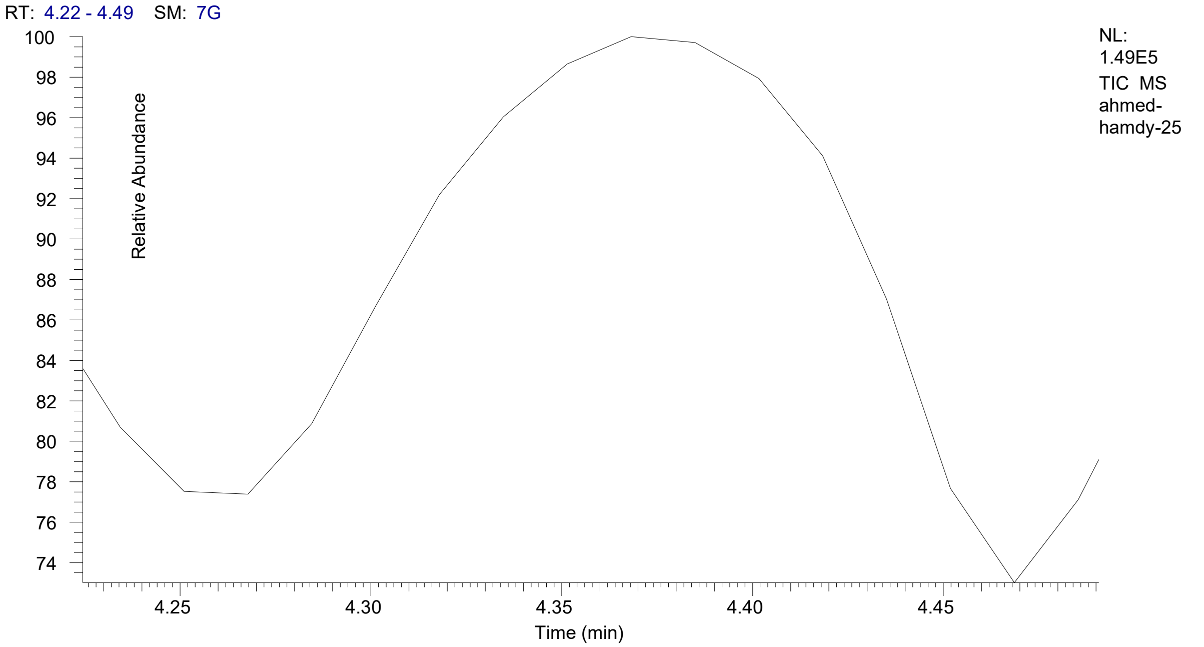

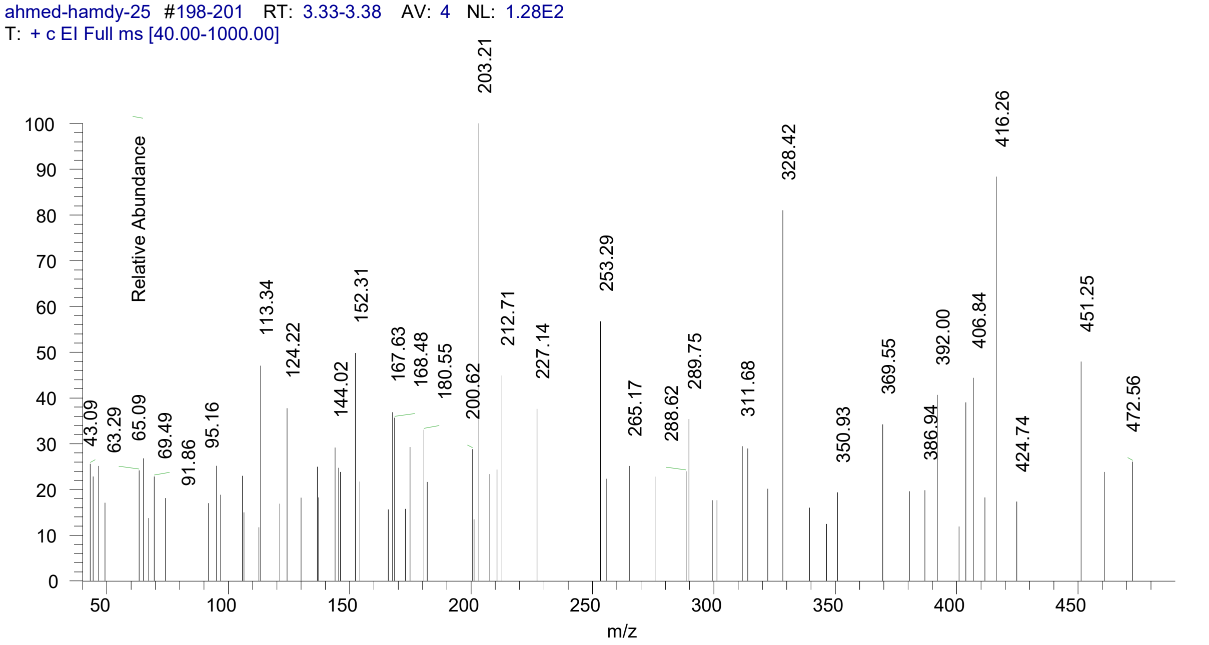

Fig S27. Mass spectra of 4c

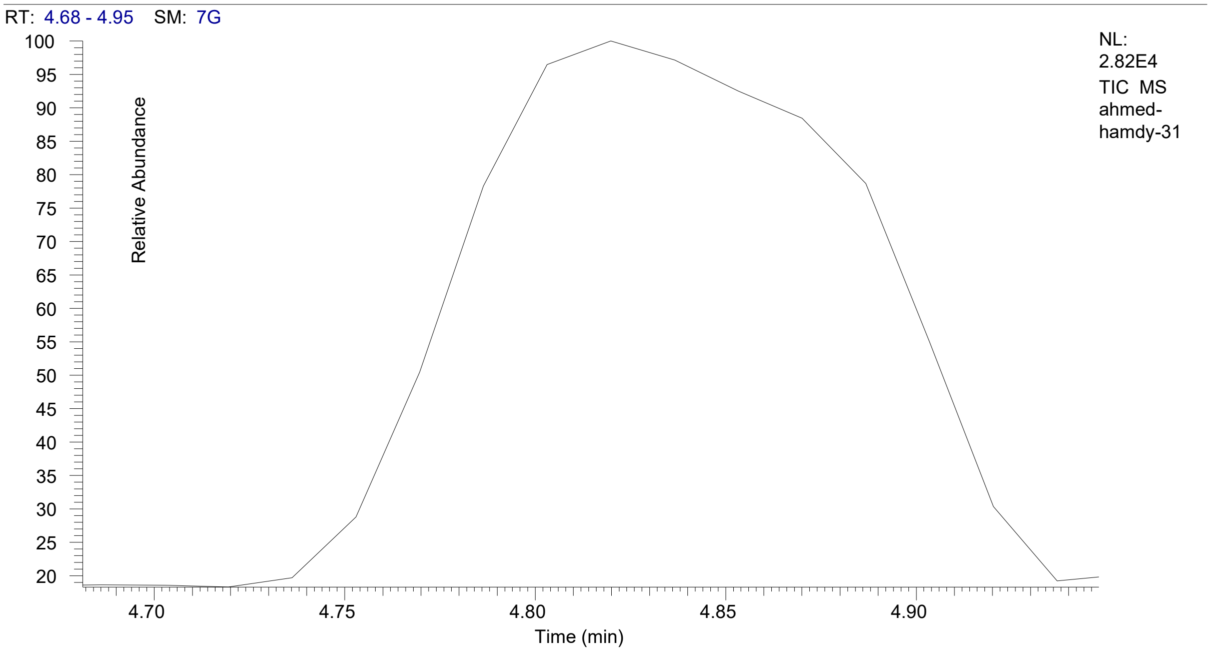

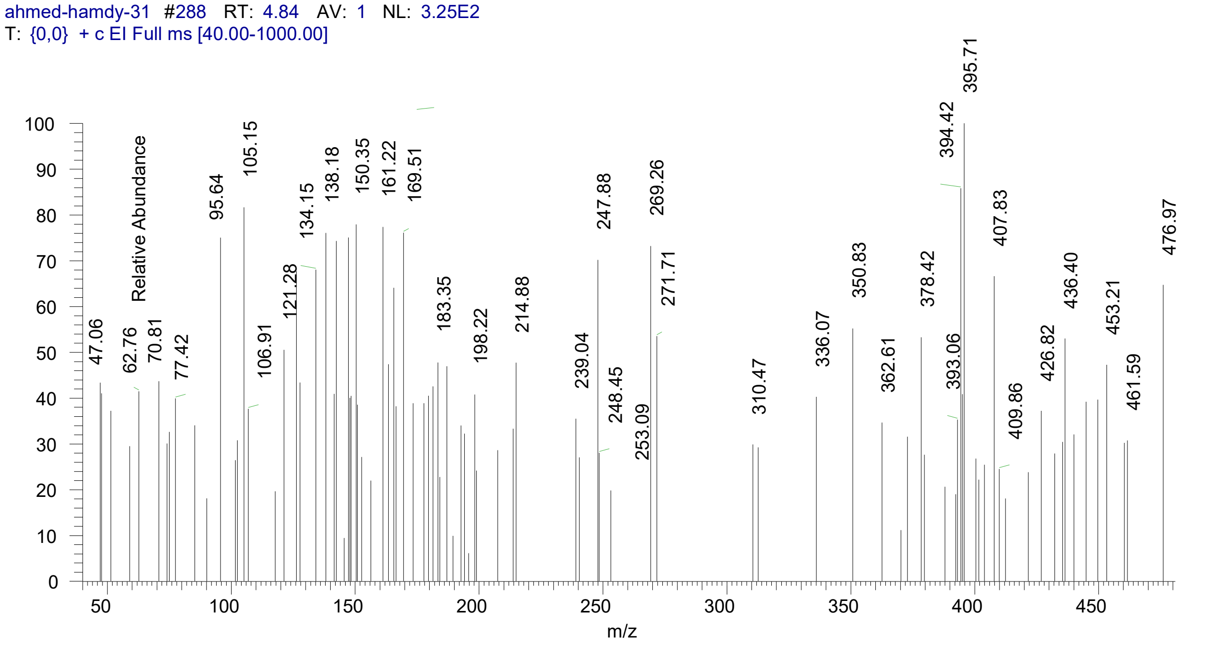

Fig S28. Mass spectra of 4d

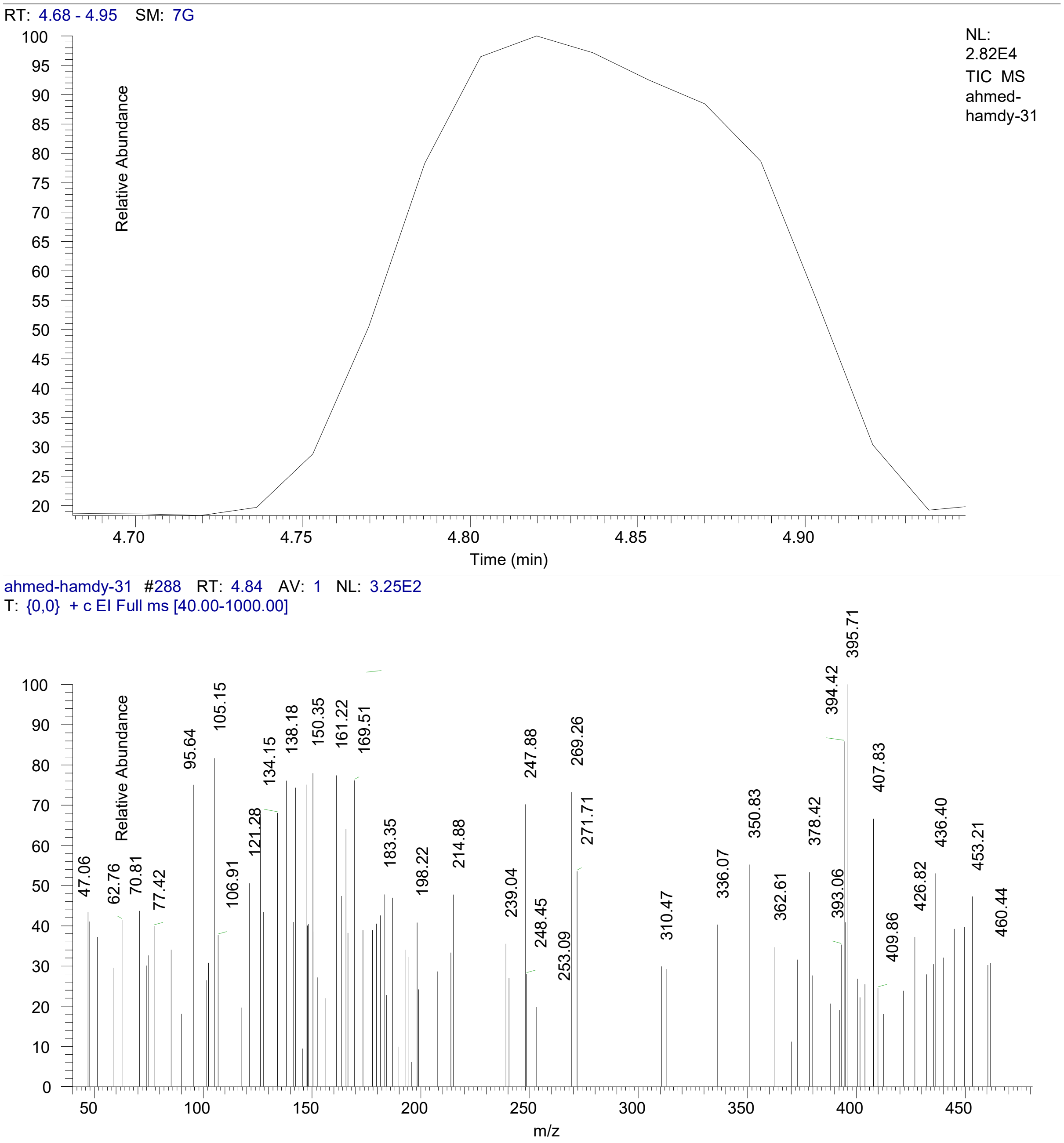

Fig S29. Mass spectra of 4e

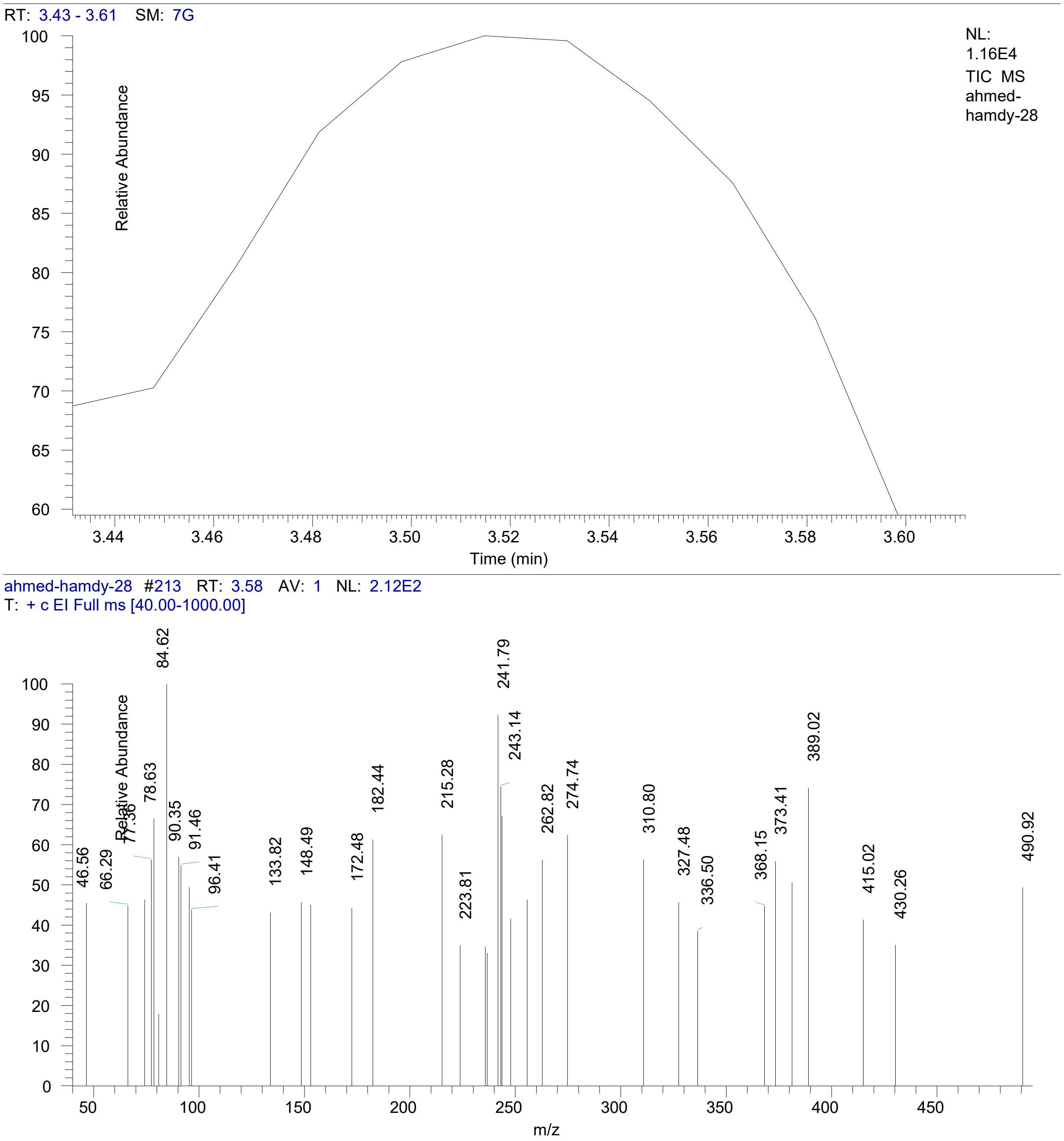

Fig S30. Mass spectra of 4j

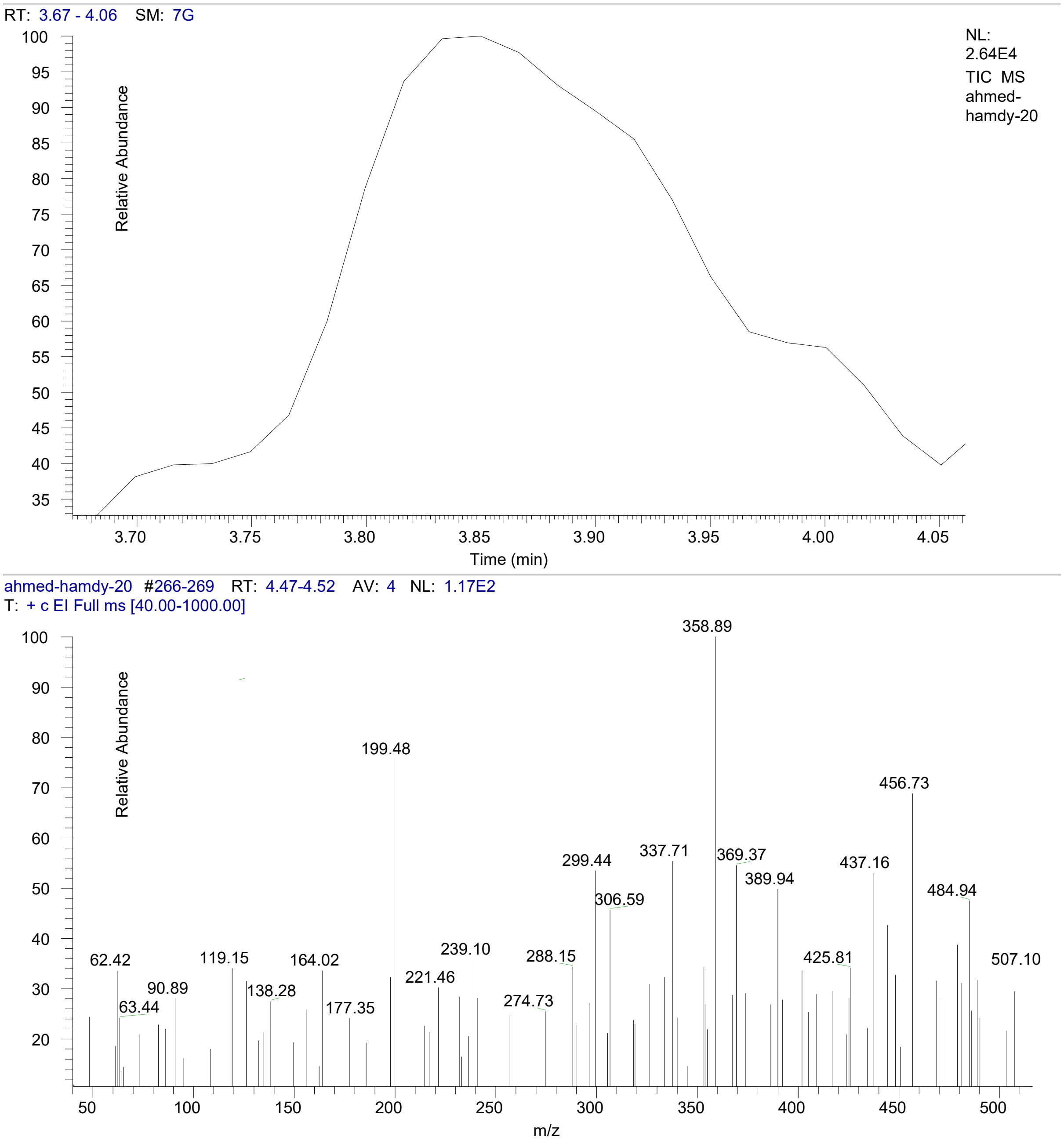

Fig S31. Mass spectra of 4o

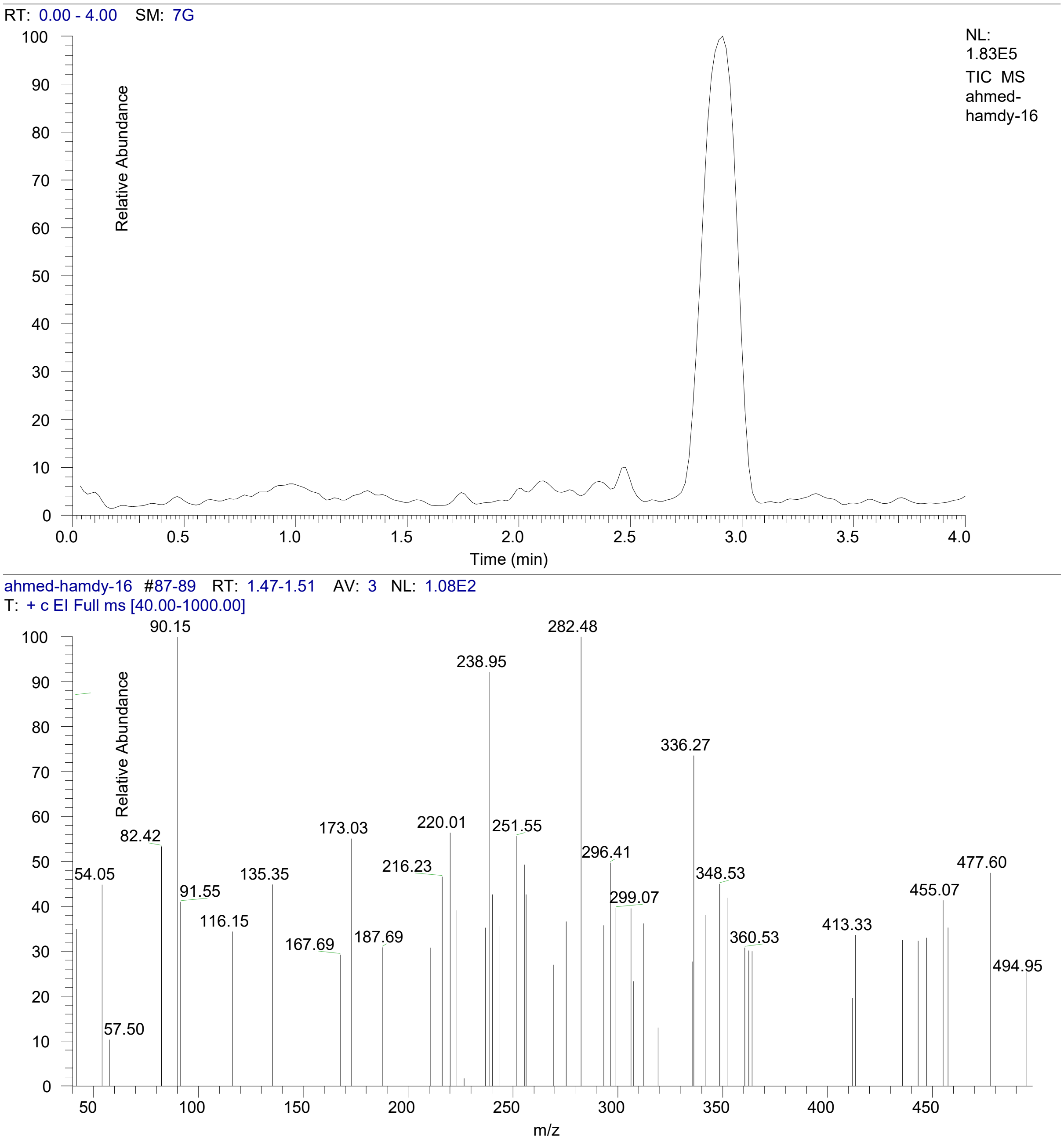

Fig S32. Mass spectra of 4q

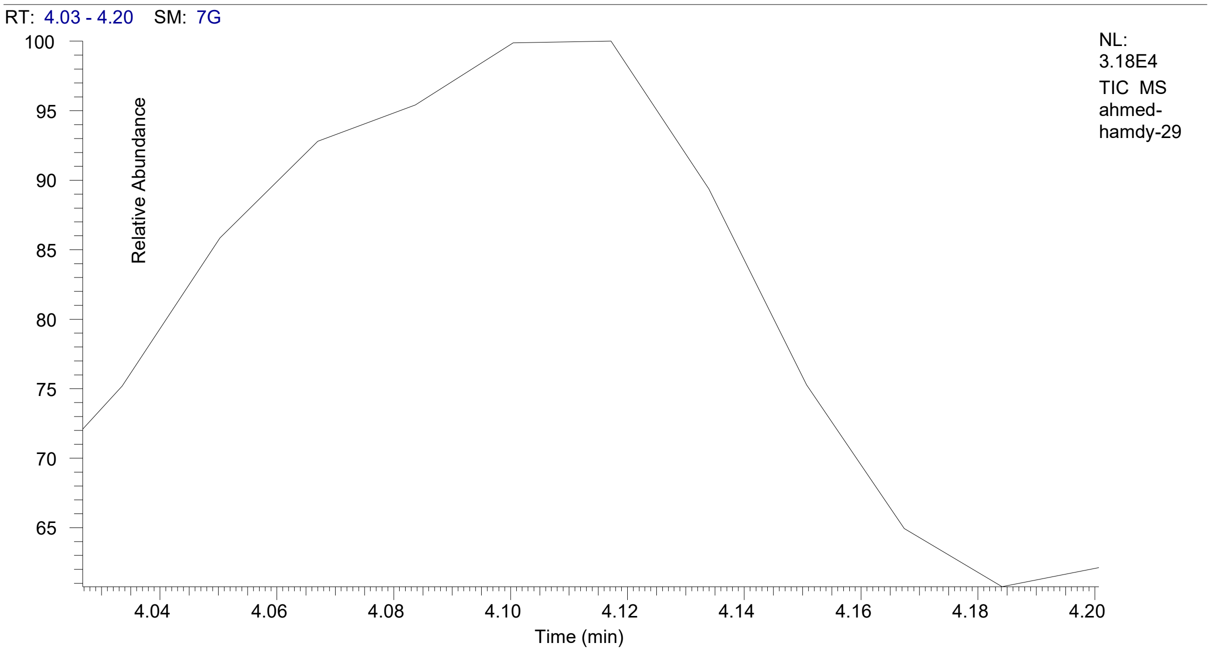

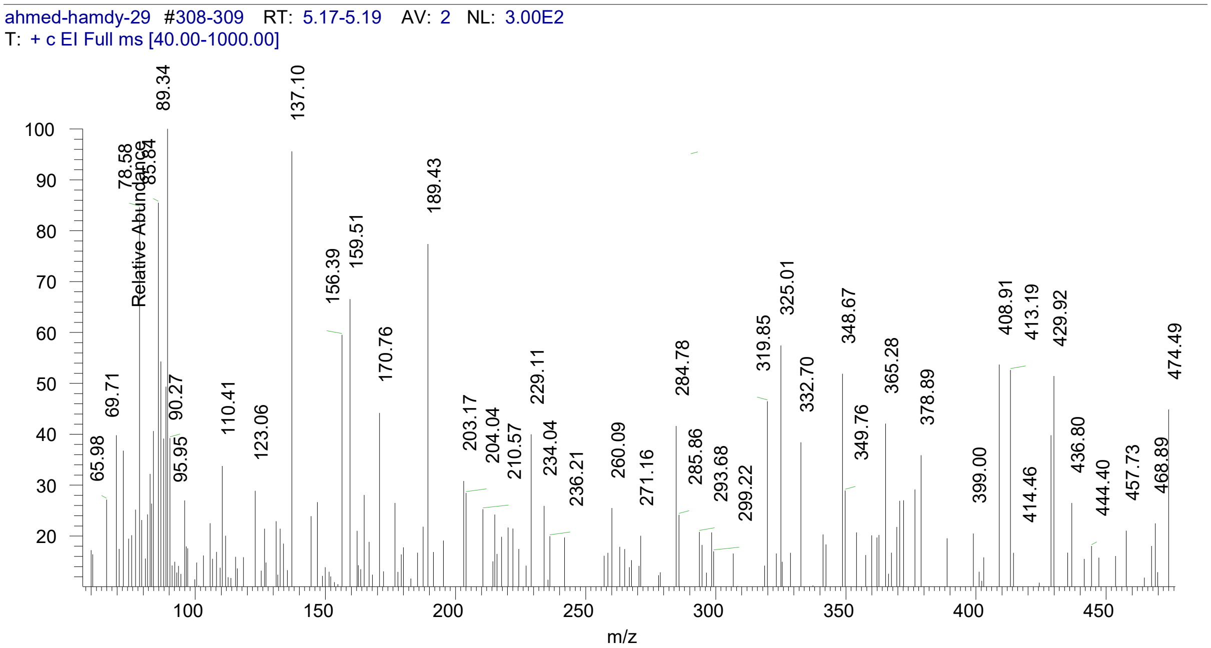

Fig S33. Mass spectra of 4k

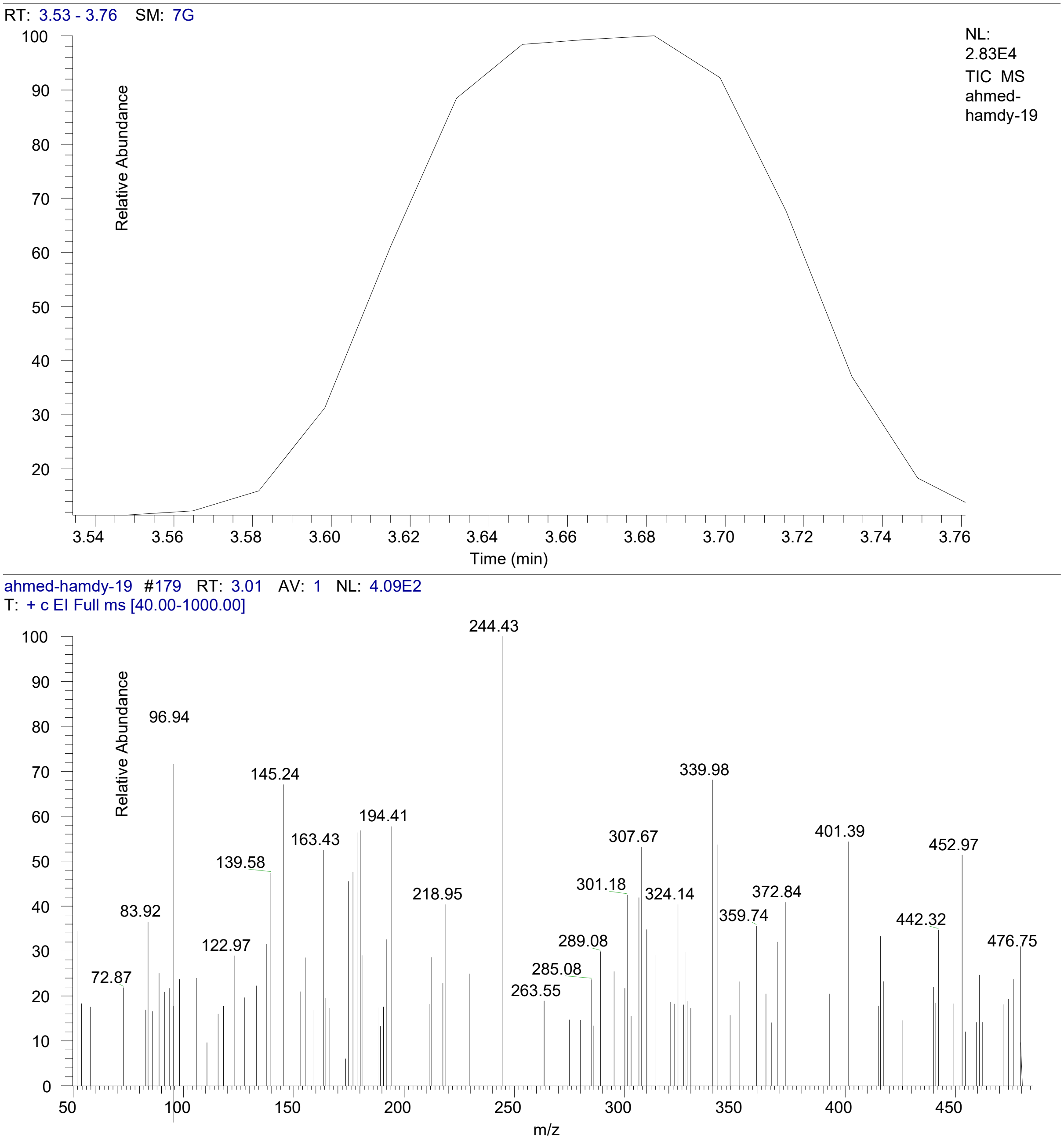

Fig S34. Mass spectra of 4m

IR

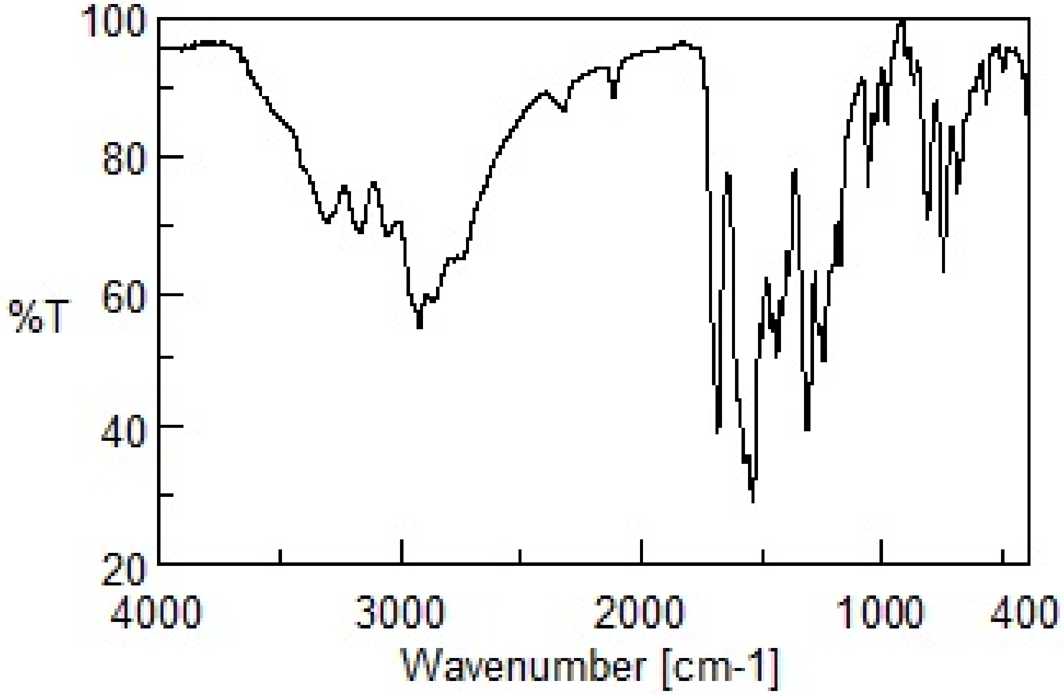

Fig S35. IR spectra of 4g

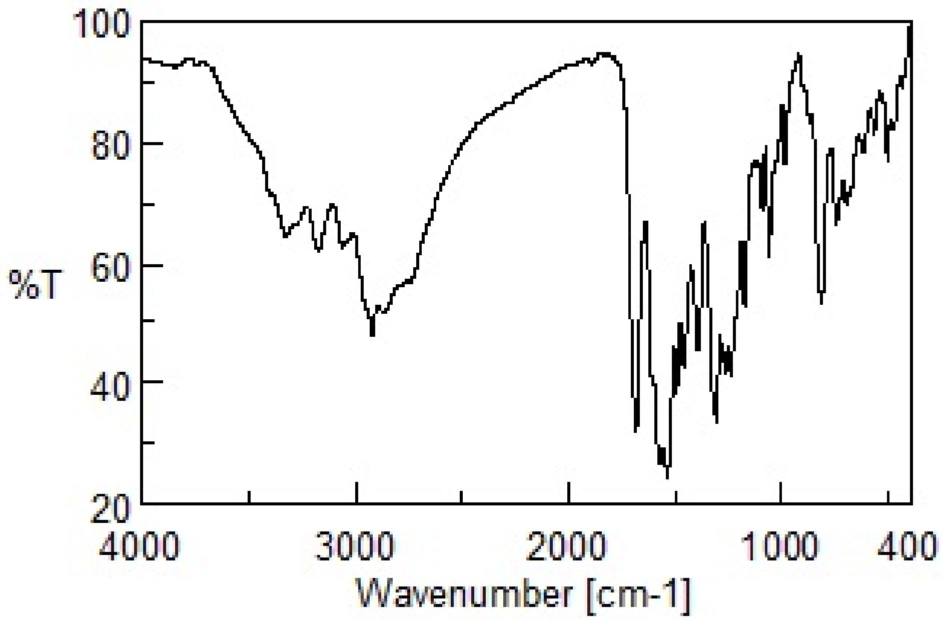

Fig S36. IR spectra of 4j

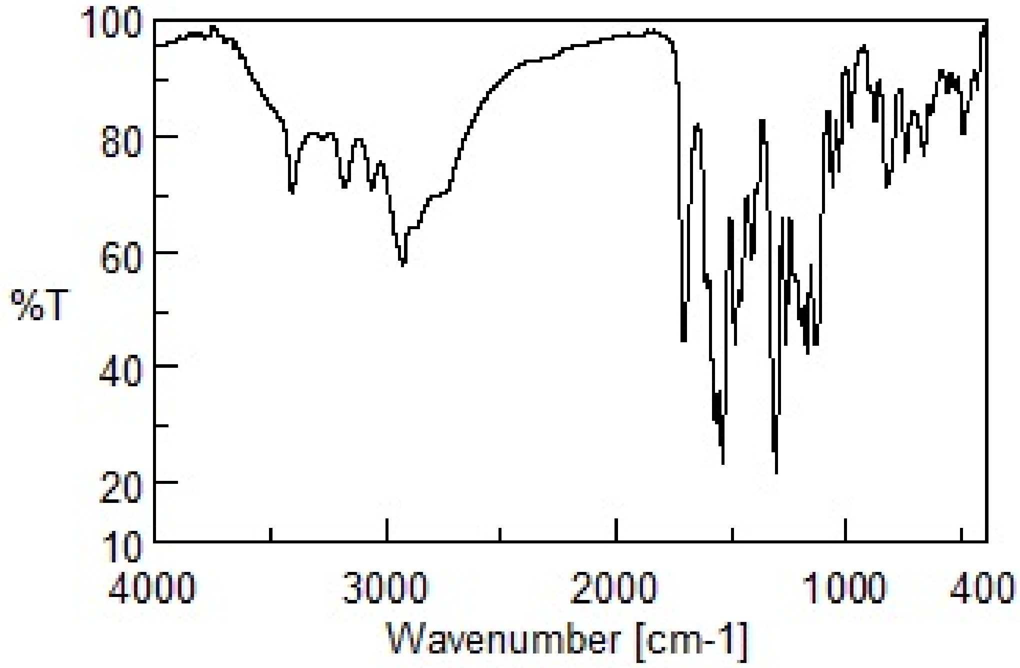

Fig S37. IR spectra of 4l

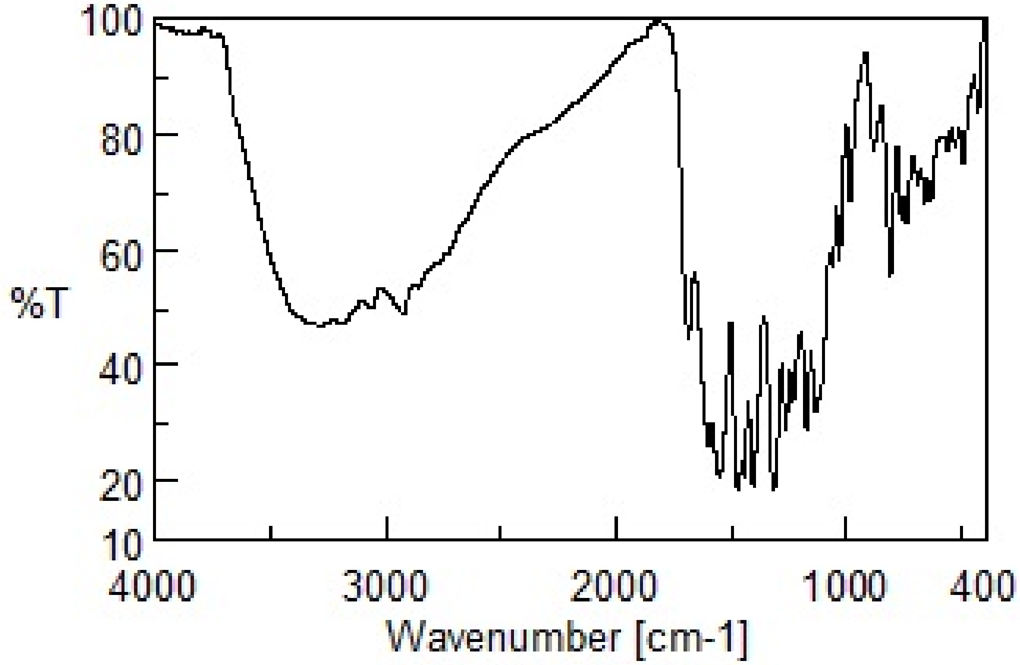

Fig S38. IR spectra of 4r

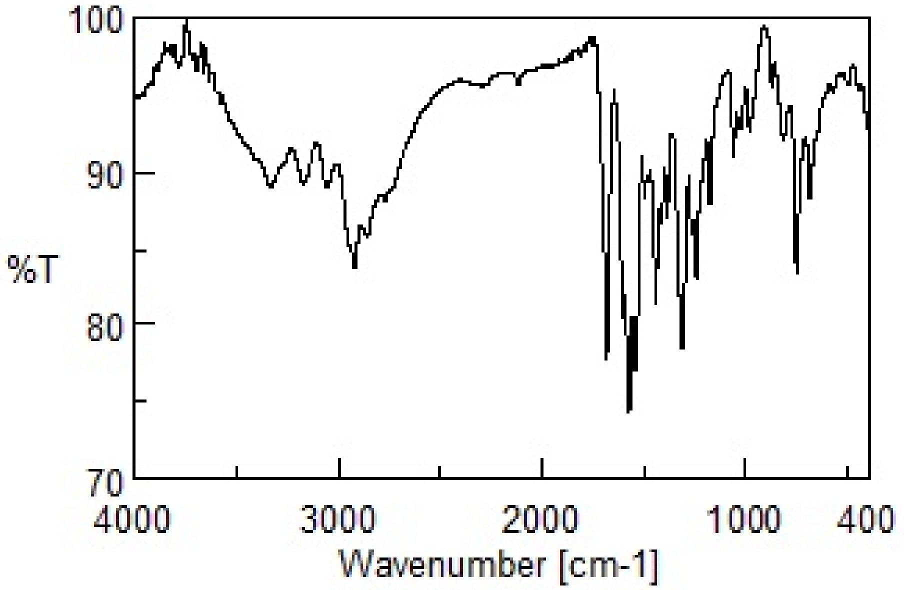

Fig S39. IR spectra of 4a

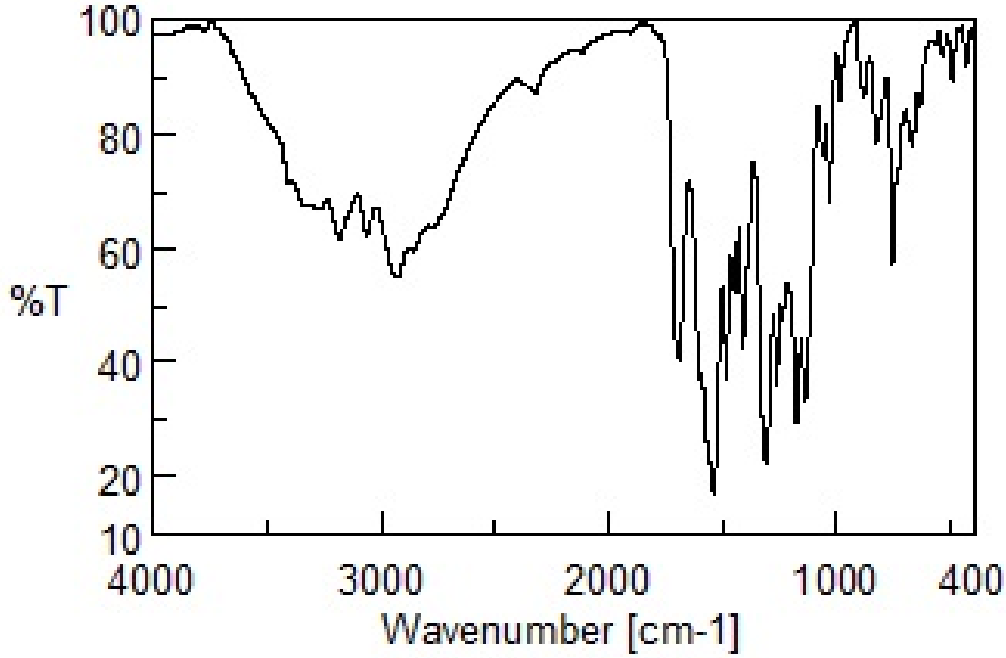

Fig S40. IR spectra of 4f
